# Mapping ovarian cellular and molecular landscape across the lifespan of women: a scoping review

**DOI:** 10.1093/humupd/dmag018

**Published:** 2026-07-17

**Authors:** Darina Obukhova, Marina Loid, Marieke Schor, Han G Brunner, Andres Salumets, Masoud Zamani Esteki

**Affiliations:** Department of Clinical Genetics, Maastricht University Medical Centre (MUMC+), Maastricht, The Netherlands; Department of Genetics and Cell Biology, GROW School for Oncology and Reproduction, Maastricht University, Maastricht, The Netherlands; Department of Obstetrics and Gynecology, Institute of Clinical Medicine, University of Tartu, Tartu, Estonia; Department of Obstetrics and Gynecology, Institute of Clinical Medicine, University of Tartu, Tartu, Estonia; Celvia CC, Tartu, Estonia; Department of Education and Support, University Library, Maastricht University, Maastricht, The Netherlands; Department of Clinical Genetics, Maastricht University Medical Centre (MUMC+), Maastricht, The Netherlands; Department of Human Genetics, Radboud University Medical Center, Nijmegen, The Netherlands; Department of Obstetrics and Gynecology, Institute of Clinical Medicine, University of Tartu, Tartu, Estonia; Celvia CC, Tartu, Estonia; Division of Obstetrics and Gynecology, Department of Clinical Science, Intervention and Technology (CLINTEC), Karolinska Institutet and Karolinka University Hospital, Stockholm, Sweden; Department of Clinical Genetics, Maastricht University Medical Centre (MUMC+), Maastricht, The Netherlands; Department of Genetics and Cell Biology, GROW School for Oncology and Reproduction, Maastricht University, Maastricht, The Netherlands; Division of Obstetrics and Gynecology, Department of Clinical Science, Intervention and Technology (CLINTEC), Karolinska Institutet and Karolinka University Hospital, Stockholm, Sweden

**Keywords:** follicle development, genome, methylome, oocyte maturation, ovarian aging, ovarian development, ovarian multi-omics studies, postmenopause, proteome, transcriptome

## Abstract

**BACKGROUND:**

With growing interest in ART, fertility preservation, and postmenopausal health of women, reproductive medicine is increasingly focused on characterizing oocytes and ovarian tissue composition, as well as understanding the molecular mechanisms that guide ovarian function throughout its lifecycle. High-throughput omics technologies have enabled the characterization of different molecular layers, leading to substantial advances in our understanding of their complex dynamics. However, not all molecular aspects are studied equally, and studies examining the same modalities often show inconsistencies, underscoring the need for data standardization and highlighting the potential for using transformative artificial intelligence and machine-learning (AI/ML) methods for ovary studies.

**OBJECTIVE AND RATIONALE:**

This study aims to evaluate how multi-omic studies have advanced our understanding of the ovarian lifecycle from fetal development to postmenopause. We systematically reviewed published studies that have investigated molecular/omic layers, including the genome, methylome, transcriptome, and proteome throughout ovarian development and aging. Our analysis identified key molecular and cellular patterns, highlighted inconsistencies across studies and addressed gaps in data analysis, interpretation, and reproducibility to guide future research.

**SEARCH METHODS:**

We conducted a systematic literature search of Medline (PubMed), Embase (Ovid), and Web of Science Core Collection (Clarivate) using a combination of controlled and free text terms for human ovary, oogenesis, folliculogenesis, ovary development and (epi)genome, transcriptome, proteome, and multi-omic mechanisms to find relevant articles published before August 2025. To focus the scope of the current review, studies of domesticated and farm animals, rodents and other model organisms, non-human primates, as well as those examining various human ovarian pathologies were excluded.

**OUTCOMES:**

The search identified 23 546 studies for screening, of which 637 full-text studies were assessed for eligibility. Subsequently, we extracted data from 121 studies. Most studies analyzed the transcriptome of oocytes, granulosa cells, and ovarian tissue from reproductive-age individuals (n = 91), with fewer studies examining samples from individuals of advanced reproductive age (n = 45) and fetal (n = 16) samples. Transcriptome analyses were most common (n = 103, 85%), followed by proteome (n = 19, 16%) and epigenome (n = 14, 12%) studies. We found substantial variation in how studies defined and reported participants’ groups as well as in their sequencing technologies and data analysis methods, with a lack of standardized reporting of background clinical information, data analysis methods, and pipeline details. The key findings underscore the prevailing consensus on genes defining major ovarian cell types and their roles throughout the ovarian lifespan, from prenatal development to postmenopausal transformation. This review highlighted the underrepresentation of certain patient groups, particularly prepubertal and peri-/postmenopausal individuals, among researched populations, due to obvious clinical and ethical reasons.

**WIDER IMPLICATIONS:**

This scoping review offers a comprehensive overview and benchmark of the current state of high-throughput omics-based research on ovarian cellular composition and molecular dynamics. To address these shortcomings, we propose general recommendations for multi-omics ovary studies and emphasize the necessity for more thorough multi-omic data integration by effectively applying novel AI/ML approaches. They can potentially improve the quality of multi-omics analyses at both single-cell and tissue levels despite limited sample sizes and enable integration of molecular profiling data with clinical and radiology datasets, enabling a more comprehensive understanding of ovarian biology. Such advancements can enhance reproducibility of research findings and guide future research to deepen our understanding of ovarian biology and ultimately support the development of medical technologies for better preserving fertility and alleviating infertility.

**REGISTRATION NUMBER:**

A protocol was published *a priori* on the Open Science Framework (https://osf.io/z38gb/).

## Graphical abstract

**Graphical Abstract:**
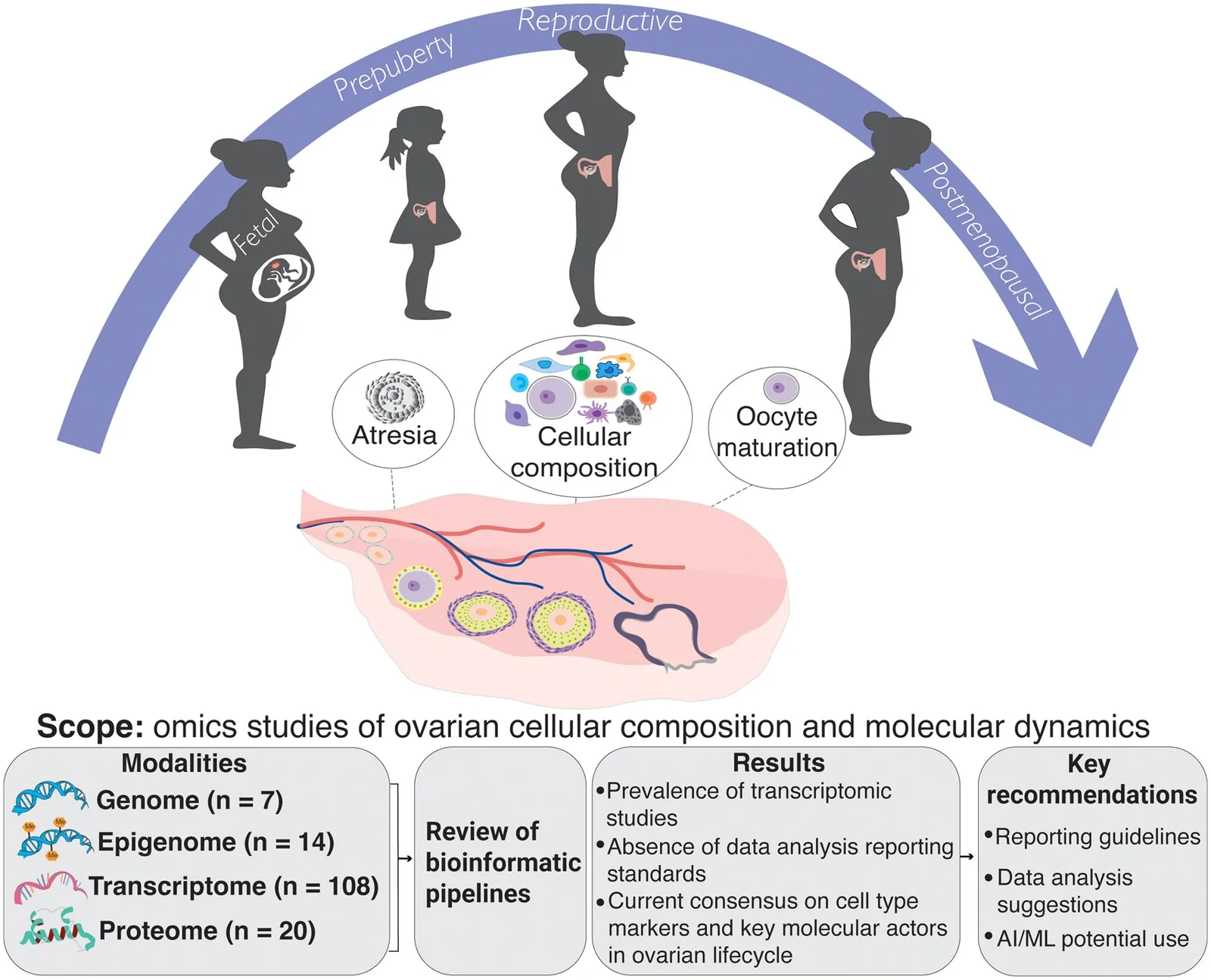
**Overview of the ovarian molecular landscape across the female lifespan assessed with omics methods.** Scoping review of omics studies (n = 121) characterizing ovarian cellular composition and molecular dynamics from fetal development to postmenopause.

## Introduction

The ovaries are glands in the female reproductive system that play a vital role in initiating life by providing mature, fertilization-ready oocytes and secreting essential hormones to prepare the reproductive tract for fertilization and to support early pregnancy. Furthermore, they regulate the reproductive timespan by serving as a pacemaker to orchestrate the onset of menarche and the transition to menopause (Wise *et al.*, 1996).

Human ovarian development begins when bipotential genital ridges form as a paired thickening of coelomic epithelium on the ventral-medial surface of the mesonephros around 5 postconceptional weeks (PCW) (Brennan and Capel, 2004; Oktem and Oktay, 2008). These ridges are then populated by primordial germ cells (PGCs), the embryonic precursors of adult gametes, most likely originating from *TFAP2A-*positive progenitors in the posterior proximal epiblast and migrating through the hindgut and dorsal mesentery (Czukiewska and Chuva de Sousa Lopes, 2022; Roelen and Chuva de Sousa Lopes, 2022; Castillo-Venzor *et al.*, 2023). After PGC colonization, gonadal sex determination begins, governed by Y-linked testis-determining factor (SRY) presence. In the absence of SRY, the bipotential gonad differentiates into the ovary at around 6 PCW, and supporting somatic cells become (pre)granulosa cells (Czukiewska and Chuva de Sousa Lopes, 2022). Upon settling in the gonad, PGCs differentiate into oogonia, which actively replicate to several million cells by 20 PCW and enter meiosis asynchronously. The resulting oocytes remain arrested in the final stage of prophase (diplotene) of the first division of meiosis (George and Wilson, 1978). By 17 PCW, flattened pre-granulosa cells encapsulate oocytes, forming primordial follicles, a functional unit of the ovarian reserve (Garcia-Alonso *et al.*, 2022). This process continues *in utero* until birth, yielding approximately one million follicles (Markström *et al.*, 2002). These quiescent follicles are stored in the ovarian cortex, i.e. the outer portion of ovary, and are continuously recruited into a growing pool of primary follicles at a rate of several hundred follicles per month, peaking at ∼15 years of age. However, folliculogenesis remains incomplete before puberty when cyclic gonadotropin stimulation and ovulation begin with hypothalamic-pituitary-gonadal axis activation (Bordini and Rosenfield, 2011). Subsequently, due to atresia, a hormonally regulated process of follicular degeneration (Hsueh *et al.*, 1994; Kaipia and Hsueh, 1997; Findlay *et al.*, 2015; Chu *et al.*, 2018), the ovarian reserve decreases to about 1000 follicles, marking the onset of menopause (∼50 years) (Wallace and Kelsey, 2010; Ruth *et al*., 2021). Only 400–500 follicles will ovulate throughout the reproductive lifespan (Wise *et al.*, 1996). Follicle development to the ovulatory stage requires nearly 1 year and depends on hormonal and paracrine factors, extracellular matrix reorganization, granulosa and theca cell division, and oocyte growth (Oktem and Oktay, 2008; Coticchio *et al.*, 2015). At ovulation, the mature oocyte, arrested in the metaphase stage of the second meiotic division, is released from the ovary and migrates into the fallopian tube, where it can be successfully fertilized within 24 h or undergo degradation, referred to as ‘postovulatory aging’ (Di Nisio *et al.*, 2022; Charalambous *et al.*, 2023). Follicular atresia occurs at every stage, typically allowing only one follicle to mature and ovulate each cycle. While its precise mechanism remains to be elucidated (Rolaki *et al.*, 2005; Chesnokov *et al.*, 2024), proposed explanations include prophase quality control (Hunt and Hassold, 2008), redundant endocrine, paracrine, and autocrine actions (Chun *et al.*, 1996), and increased granulosa cell apoptosis, possibly linked to impaired testosterone synthesis and loss of estrogen signaling (Wei *et al.*, 2023).

Understanding the interplay between molecular layers involved in ovarian development and folliculogenesis is crucial for addressing reproductive issues, understanding aging, and advancing *in vitro* gametogenesis, ultimately improving female fertility preservation programs and fertility treatment options for PCOS patients. High-throughput omics methods profile these layers (genome, transcriptome, epigenome, proteome, and metabolome) by quantifying and characterizing the molecules within each layer in biological samples (Kumar *et al.*, 2019). They have become standard tools, continuously advancing towards single-cell resolution and spatial mapping, enabling cell lineage tracing and generation of organ atlases that enhance our understanding of how molecular layers interact in health and disease (Baysoy *et al.*, 2023). Importantly, recent advancements in artificial intelligence and machine learning (AI/ML) models hold promise for elucidating molecular processes and understanding the complex cellular landscape of ovarian tissue and its regulation.

In this review, we systematically reviewed published studies that investigated the (multi)omic mechanisms regulating folliculogenesis, oogenesis, and other ovarian cell dynamics throughout the different stages of its lifecycle, from fetal development to postmenopause, identifying knowledge gaps and exploring possible future research directions in this field.

## Methods

This study follows the Preferred Reporting Items for Systematic Reviews and Meta-Analyses extension for Scoping Reviews (PRISMA-ScR). A protocol was published *a priori* on the Open Science Framework (https://osf.io/z38gb/).

### Search strategy

A comprehensive literature search of Medline (PubMed), Embase (Ovid), and Web of Science Core Collection (Clarivate) was conducted using a combination of controlled vocabulary and free text terms for human ovary, oogenesis, folliculogenesis, ovary development and (epi)genome, transcriptome, proteome, and multi-omic mechanisms to find relevant articles published between 2008, marking the emergence and wider adoption of next-generation sequencing (NGS) technologies, and August 2025. Full search details are described in Supplementary File S1. CitationChaser R package (v.0.0.4) was used to examine the reference lists of included studies and find additional relevant studies not retrieved by the database search (Haddaway *et al.*, 2022). Notably, a few omic ovarian studies have been published after this review’s cut-off date of August 2025 and were therefore beyond the scope of this review.

### Study selection and eligibility criteria

Titles of deduplicated citations from all databases were filtered to exclude those that did not include female gonad-related terms using a combination of the ‘filter’ function from the tidyverse R package (v.2.0.0), and the ‘grepl’ function from base R. Filtered citations were imported into Rayyan (v.1.4.3) (Rayyan Systems Inc., Cambridge, MA, USA). Two authors (D.O., M.L.) independently screened all titles and abstracts to determine which studies would undergo full-text review. Any disagreements between reviewers were resolved through discussion.

Only studies using human samples were considered. Studies using cryopreserved or cultured ovarian tissue were included; however, studies comparing ovarian cryopreservation protocols, *in vitro* culture protocols, ovarian tissue preparation methods, or ovarian stimulation protocols were excluded. Studies involving only non-human species, primary ovarian cell lines, targeted DNA sequencing limited to a small set of specific genes, or those without the use of high-throughput profiling (NGS or mass spectrometry) were also excluded. Additionally, studies focusing on ovarian hyperstimulation syndrome, spontaneous oocyte activation, polycystic ovary syndrome (PCOS), ovarian cancer, primary ovarian insufficiency (POI), the effects of hepatitis B on ovarian function, the relationship between oocyte physiology and blastocyst development, or ovarian response to viral or bacterial infections were excluded. Reviews, editorials, commentaries, meta-analyses, conference abstracts, and preprints were also excluded (Supplementary Table S1). Only studies published in English were considered.

### Data extraction and synthesis

Data extraction of eligible papers was done in Google Sheets independently by one author and checked by the other author. We extracted citation details, participant characteristics (age, group, treatment, exclusion criteria), descriptive details of study design (single-cell/bulk, cell types analyzed), omic(s) profiled (layers profiled, number of samples/cells, sample preparation method, sequencing specifications), computational analyses (methods, tools), data availability (repository identifiers), and key findings. Studies were grouped by reproductive life stage (fetal, (pre)pubertal, reproductive age, post(menopausal)), according to the categories used in the original publications.

## Results

### Systematic search and summary statistics

**Figure 1.**
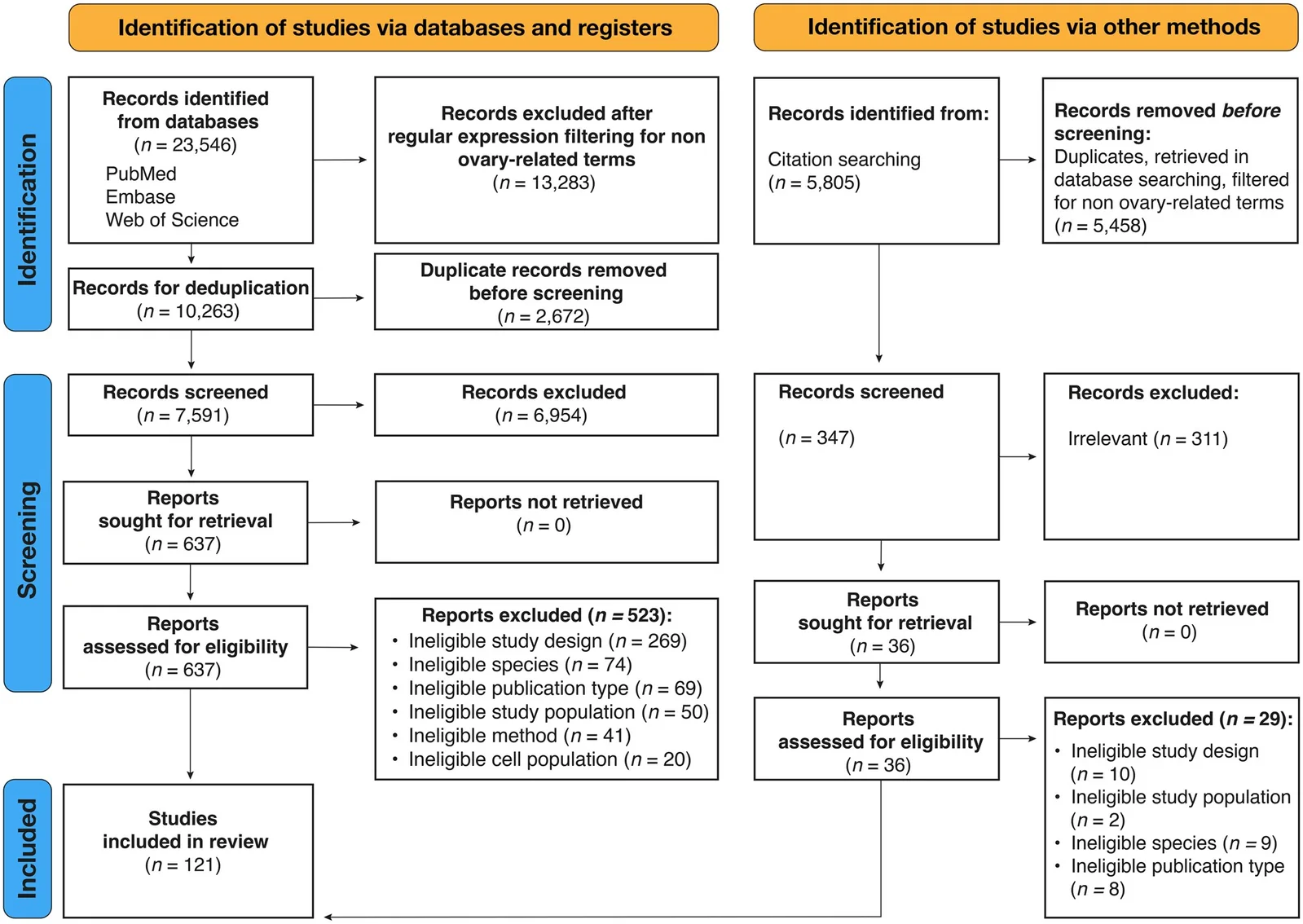
**PRISMA flowchart for the selection of studies for a scoping review of ovarian cellular and molecular dynamics across the female lifespan.** PRISMA, Preferred Reporting Items for Systematic Reviews and Meta-Analyses. For more information, visit: http://www.prisma-statement.org/.

Our search identified 23 546 studies. After filtering for ovary- and follicle-related terms in titles, 10 263 studies remained. Following deduplication and abstract screening, 637 full-text studies were assessed for eligibility, of which 523 were excluded (Fig. 1). The most common reasons for exclusion were ineligible study design (n = 273, 52%) and ineligible publication type (n = 72, 14%), followed by use of non-human samples (n = 69, 13%), ineligible study population (n = 54, 10%), absence of high-throughput profiling (n = 36, 7%), and ineligible cell population (n = 19, 4%). Ultimately, 114 studies met our inclusion criteria, with an additional 7 studies found through citation chasing, ending up with 121 studies in total.

**Figure 2.**
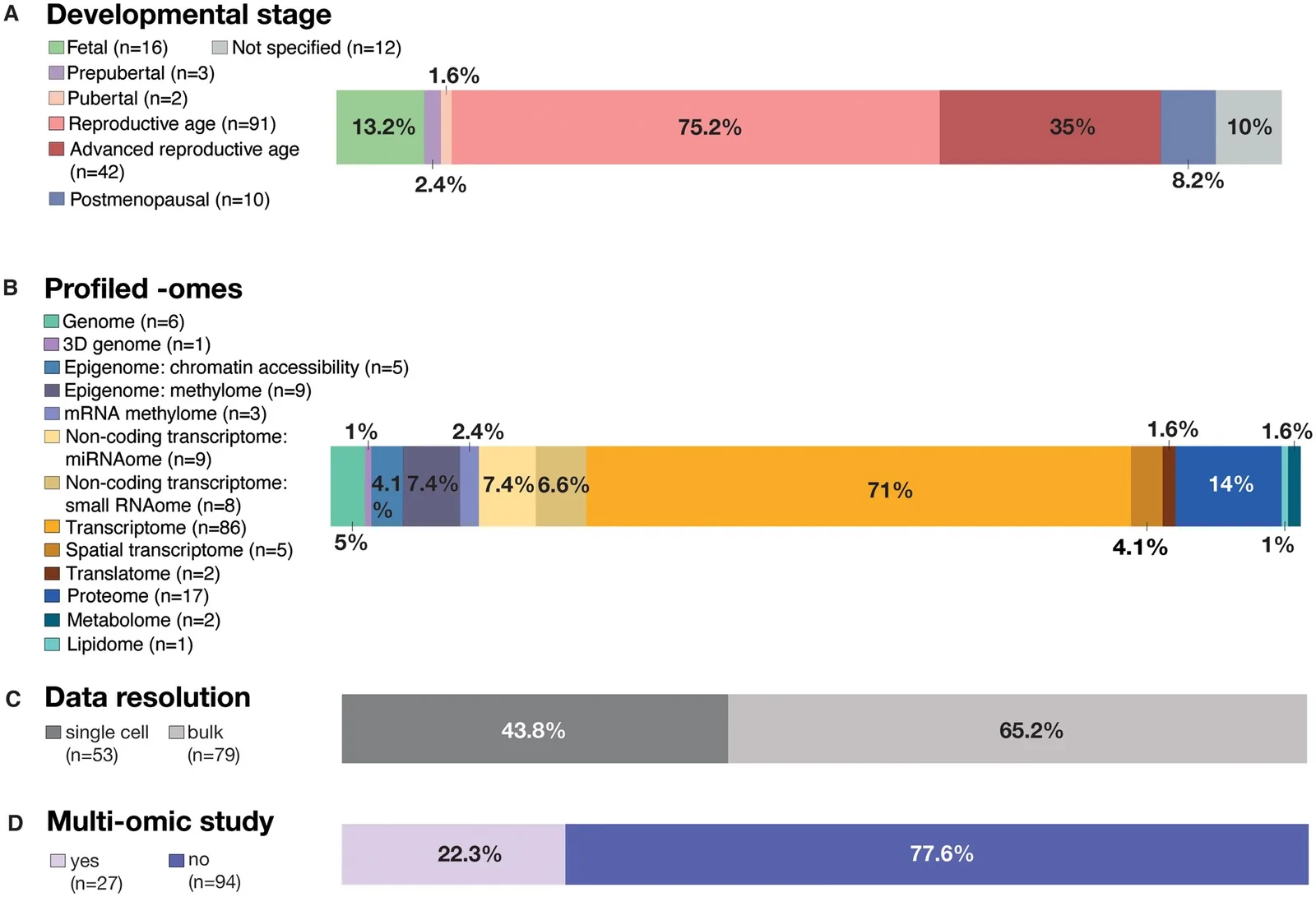
**Overview of studies included in high-throughput ovarian research review.** (**A**) The percentage of developmental stages reported in the studies. Some studies included multiple age groups. (**B**) The percentages of the profiled omics layers. Some studies profiled multiple omics layers. (**C**) The percentages of data resolution, used in the studies. (**D**) The percentages of studies that integrate multiple omics layers (e.g. genome, transcriptome) or focus on a single omics layer.

Most studies (n = 91, 75.2%) included participants of reproductive age, commonly defined as the period from menarche to menopause (Nabhan *et al.*, 2022) (Fig. 2A). An alternative concept is the effective reproductive span, reflecting both social and biological factors (Nabhan *et al.*, 2022). This spans from the typical onset of sexual activity at 18 years (Finer and Philbin, 2013) to the age when accelerated oocyte decline and follicle loss occur, an endpoint variably defined as happening between 35 and 38 years (Bozdag *et al.*, 2016; Faddy *et al*., 1992; Forman *et al*., 2013). Of these 91 reproductive age studies, 43 also included participants of advanced reproductive age, commonly defined as from age 38 until menopause, typically occurring around age 50–51 in high-income countries and age 49 in socioeconomically diverse regions (Schoenaker *et al.*, 2014). Twelve studies (10%) investigated adult participants without specifying their ages. Postmenopausal women were included in 10 studies (8.2%), while fetal development was the third most studied stage (n = 16, 13.2%). Prepubertal and pubertal girls were least represented, comprising only 2.4% and 1.6% of samples respectively, highlighting a crucial gap in the available study material for these groups.

### Omics studies of folliculogenesis and oogenesis

Among the 121 human ovary studies, transcriptomics dominated (n = 108, 89%), followed by proteomic (n = 17, 14%), epigenomic (n = 14, 11.6%) and genomic (n = 7, 6%) profiling (Fig. 2B, Supplementary Table S2).

For transcriptomics, expression microarrays were used in 23 studies (19%), declining from 86% (2009–2014) to 9% (2021–2025), superseded by RNA sequencing (RNA-seq). RNA-seq provides comprehensive coverage of absolute expression levels and more sensitive detection of RNA molecules, including the less abundant ones (Mortazavi *et al.*, 2008). While bulk RNA-seq has improved in throughput and handling of multi-mapped reads and amplification bias (Stark *et al*., 2019), reviewed studies used similar library preparation and sequencing protocols, suggesting that differences between them likely reflect variation in sample size, sequencing depth, and biological heterogeneity rather than technical factors. Recently, the number of ovarian studies using single-cell transcriptomic approaches increased. They range from full-length, low-input methods for detailed characterization of individual cell transcriptomes to high-throughput microfluidic platforms such as 10× Genomics, which provide a broader view of tissue-level cellular composition. These methods address different experimental questions and can complement each other to comprehensively characterize cellular transcriptomes and tissue composition. Additionally, capturing oocytes with 10× Genomics is challenging due to its 30 µm size limit and clogging risks, requiring alternatives like laser-capture microdissection (LCM) or direct cell lysis, sometimes coupled with cell sorting (Machlin and Shikanov, 2022). However, transcriptome data alone provide only a partial view as post-transcriptional processes, such as mRNA methylation, regulate translation and mRNA fate. Integrative approaches such as epitranscriptomic or translatomic profiling offer a fuller picture of ovarian gene regulation (Hu *et al.*, 2022; Huang *et al.*, 2023; Yao *et al.*, 2025). As mRNA-protein abundance correlation is moderate for most genes (Buccitelli and Selbach, 2020), integrating transcriptomic and proteomic data is essential, particularly at the single-cell resolution. Our analysis identified four single-cell oocyte proteomic studies showing a high overlap in detected proteins (Virant-Klun *et al.*, 2016; Guo *et al.*, 2022; Galatidou *et al.*, 2024; Zhang, Yang, *et al.*, 2025). They show bulk ovarian proteomes mainly reflect somatic cells as only 5–7% of the most abundant ovarian tissue proteins overlap with single oocyte proteins (Uhlén *et al*., 2015). Moreover, spatial mapping of ovarian gene expression helps to understand cell communication and dynamics of ovarian microenvironments (Jones *et al.*, 2024; Kavarthapu *et al.*, 2024; Pei *et al*., 2024; Wu *et al.*, 2024).

Genomic studies were fewer (n = 7, 6%) but provided insights into the chromosome landscape. One used single-cell triple omics sequencing sc-CHARM-seq to measure genome copy number variations, methylome, and transcriptome (Ye *et al.*, 2020), while two analyzed oocyte ploidy using array comparative genomic hybridization (aCGH) or NGS (Barone *et al.*, 2020; Ghevaria *et al.*, 2022). NGS outperforms aCGH in detecting mosaic and segmental aneuploidies (Lai *et al.*, 2017). Other studies interrogated oocyte crossovers and chromosome segregation (Hou *et al.*, 2013), characterized mitochondrial genome variation across oocyte maturation stages and age (Boucret *et al.*, 2017; Arbeithuber *et al.*, 2025), and investigated 3D genome organization in granulosa cells (Mao *et al.*, 2024).

Epigenome profiling encompassed single-cell chromatin accessibility (Yan *et al.*, 2021; Garcia-Alonso *et al.*, 2022; Lengyel *et al.*, 2022; Guahmich *et al.*, 2023; Jin *et al.*, 2025) and methylation studies. Most methylation analyses used whole-genome bisulfite sequencing (WGBS) (Gkountela *et al.*, 2015; Guo *et al.*, 2015; Yu *et al.*, 2017), while two employed methylation microarray (Olsen *et al.*, 2020; Jain *et al.*, 2024) or reduced representation bisulfite sequencing (RRBS) (Yu *et al.*, 2015; Ye *et al.*, 2020). WGBS provides broader genomic coverage than RRBS, although neither method captures the complete methylome (Beck *et al.*, 2022).

### Population characteristics

#### Age

Across 16 studies analyzing fetal female gonadal tissue, definitions of fetal age and methods to assess it varied substantially, indicating inconsistency between studies. Definitions such as gestational age, despite recommendations against its use due to ambiguity (O’Rahilly and Müller, 2010), developmental age, and developmental stage were used, without consensus on time units. These included ‘weeks post conception (PCW)’, ‘weeks post fertilization (WPF)’, ‘weeks of gestation’, ‘days post fertilization (DPF)’, and ‘developmental days’. Morphology-based age estimation was used in six studies and included Carnegie or Streeter’s staging, foot length, heel-to-knee length, and crown-rump length, sometimes in combination. Using multiple fetal parameters for age assessment is crucial, as it increases estimation accuracy (Wong, 2017), whereas reliance on a single variable, such as crown-rump length, can be suboptimal (MacGregor *et al.*, 1987). Two of three studies using the Carnegie staging failed to specify the reference chart, a crucial omission given the variations across datasets, particularly regarding embryonic length and age (Flierman *et al.*, 2023). Two studies used fetal ultrasound, the method recommended for gestational age estimation by the World Health Organization and the American College of Obstetricians and Gynecologists (ACOG) (Committee Opinion No 700: Methods for Estimating the Due Date, 2017). One study relied on the last menstrual period date, despite its known unreliability due to inaccurate last period recall, cycle variability, and ovulation timing (Savitz *et al.*, 2002; Wegienka and Baird, 2005). Four fetal studies (Fowler *et al.*, 2009; Houmard *et al.*, 2009; Guo *et al.*, 2015; Williams *et al.*, 2015) failed to describe their age determination method, despite its importance for organ development studies.

In 91 analyzed studies examining tissue from reproductive-age patients, there was no consensus regarding the definition of reproductive age or its upper threshold (Supplementary Table S2). Thirteen studies used ‘younger than 35’ a historical benchmark for female fertility associated with declining fecundity and increasing infertility risk (Leridon, 2004; Rothman *et al.*, 2013; Steiner and Jukic, 2016). Four studies raised this threshold to 36 or 39 years (Ouandaogo *et al.*, 2012; Shi *et al.*, 2022; Takeuchi *et al.*, 2022; Cadenas *et al.*, 2023).

Thirty-one studies compared reproductive age groups, primarily focusing on younger and older groups, with six studies also including intermediate age groups. Younger groups predominantly included participants up to age 30, likely because this age range is associated with higher oocyte quality (Navot *et al.*, 1991; Venetis *et al.*, 2019). Most older groups included participants aged 40 and above, with upper limits ranging from 42 to 49 years. Studies analyzing postmenopausal women included participants from 52 years of age, slightly above the reported median age of 51.4 years for menopause in high-income countries (Gold *et al.*, 2013; Zhu *et al.*, 2019).

#### Other reported characteristics

Most fetal ovary studies (n = 10, 62%) did not report the reasons for pregnancy termination or whether it was medically indicated. This information is crucial as termination due to conditions like maternal pre-eclampsia and infection or fetal aneuploidies can impact fetal organ systems, including the reproductive system (Megli and Coyne, 2022). Other maternal variables were also not reported, except for age, specified in three studies (Fowler *et al.*, 2009; Mamsen *et al.*, 2017; Lecluze *et al.*, 2020), and smoking and alcohol consumption status documented in one (Mamsen *et al.*, 2017). Suboptimal conditions during pregnancy, including smoking or exposure to cigarette smoke, can affect the fetal initial follicle count, oocyte atresia rate (Fowler *et al.*, 2014; Cheng *et al.*, 2018), and impair oocyte-follicle communication and regulation (Lutterodt *et al.*, 2009). Furthermore, reporting maternal exposure to chemical toxicants might be important, as studies in animal models have shown it affects fetal primordial follicle pool formation, oocyte growth, and sex steroid hormone production (Hunt *et al.*, 2012; Legoff *et al.*, 2019; Lim *et al.*, 2023).

**Figure 3.**
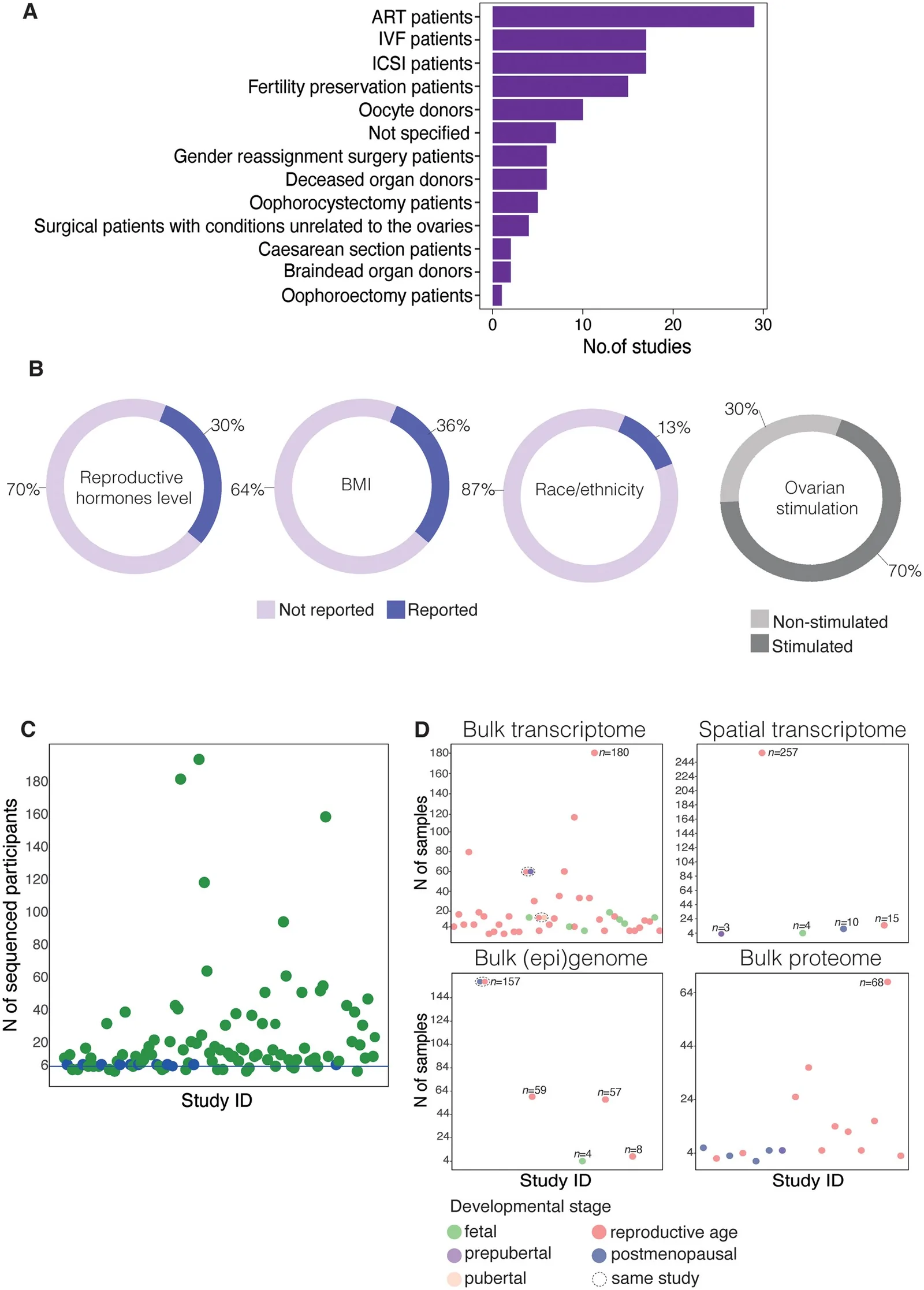
**Overview of patient characteristics and numbers from studies included in the review.** (**A**) A barplot displays the numbers of patients in different categories reported in the studies. (**B**) Donut plots present the percentage distribution of patients’ characteristics reported in the studies. (**C**) Scatterplot shows the number of sequenced participants per study. (**D**) Scatterplots demonstrate the number of samples in different bulk studies per omic level analyzed.

Our analysis identified 11 participant categories in the subset of ovarian studies, including samples from reproductive age to postmenopausal women (Fig. 3A). Among the adult study cohorts, most studies (n = 29; 28%) involved all ART patients irrespective of treatment received, while others distinguished between *in vitro* fertilization (IVF) (n = 14) and intracytoplasmic sperm injection (ICSI) (n = 15) patients. Reasons for ART were described in 28 studies, with patients grouped accordingly. These reasons included male infertility (n = 17), tubal or uterine factors (n = 12), preimplantation genetic diagnosis (n = 2), and advanced maternal age (n = 3).

Fertility preservation patients constituted the third most common category, appearing in 17 ovarian studies. Reasons for undergoing fertility preservation varied, with most studies (n = 11) including heterogeneous groups of oncological patients. Gynecological cancers were predominant, with cervical cancer appearing in four studies (n = 10 patients) and endometrial cancer in two studies (n = 10 patients). Among non-gynecological cancers, lymphoma patients were included in four studies (n = 12 patients), while breast cancer patients appeared in three studies (n = 25 patients). Six studies did not specify reasons for fertility preservation, broadly categorizing them as ‘oncology‘ or ‘malignant/benign diseases unrelated to the ovary’. Detailed reporting of patient diagnoses is crucial, since ovarian reserve, measured by antral follicle count and anti-Müllerian hormone (AMH) levels, can vary even before treatment. For instance, ovarian reserve prior to treatment in hematological cancers such as lymphoma has been reported to be reduced (Lawrenz *et al.*, 2012; Paradisi *et al.*, 2016), whereas breast cancer shows no such association (Wu *et al.*, 2023). Treatment was rarely reported: only one study specified chemotherapy exposure and the medications used (Rooda *et al.*, 2024). Chemotherapy prior to sample collection can disrupt follicular development, damage DNA in major ovarian cell types, alter the microenvironment, and trigger cellular senescence (Ding *et al.*, 2019; Guo *et al.*, 2024). Therefore, it should be clearly stated whether the ovarian samples were collected before, during or after oncological treatment.

Cadaveric ovaries were used in eight studies. Four of them utilized GTEx database bulk tissue samples from 180 individuals aged 20–79, collected within 24 h of cardiac cessation (GTEx Consortium, 2013). Of the remaining four, only two reported the cause of death (Guahmich *et al.*, 2023; Jin *et al.*, 2025).

Of 105 studies examining subjects from reproductive age to menopause, most lacked detailed clinical characterization. Body mass index (BMI) was the most frequently reported variable (36% of studies), followed by reproductive hormone levels, including follicle-stimulating hormone (FSH), AMH, and estradiol, reported in 30% of studies (Fig. 3B). Less commonly reported variables included live birth history (8%) and menstrual cycle regularity (3%). Although most studies (n = 75; 71%) included women who underwent ovarian stimulation, only 44% reported the exact protocols used, with others only mentioning using a standard ART protocol for IVF/ICSI (Supplementary Table S2). Reporting this is important because different ovarian stimulation treatments can affect granulosa and cumulus cells (Gatta *et al.*, 2013; Haas *et al.*, 2014). Studies with *in vitro* matured (IVM) oocytes did not specify the HRT protocol used before ovarian puncture. Despite systemic menopausal hormonal therapy being the first-line treatment for menopause symptoms (Mukherjee and Davis, 2025), only one study of seven postmenopausal studies documented steroid hormone drug use and treated it as an exclusion criterion (Yi *et al.*, 2018).

Notably, only 4% of the studies reported lifestyle factors such as smoking and alcohol consumption. However, these might be important variables to consider, as research shows they affect ovarian reserve size, cell development and quality, disrupt steroidogenesis by altering levels of estrogens and progesterone through changes in certain enzyme activities and hormone receptor expression (Sharara *et al.*, 1994; de Angelis *et al.*, 2020). Race was also underreported, noted in only 13% of the studies. Previous studies suggested links between race and both ovarian reserve and responsiveness to ovarian stimulation (Bleil *et al.*, 2014; Lee *et al.*, 2023).

### Population and sample size

Most studies (64%) profiled between 2 and 20 participants, with 6 participants being the most common sample size, appearing in 9% of studies (Fig. 3C). Only four studies, all using GTEx ovarian samples, included over 100 sequenced participants (Chen *et al.*, 2022; Liu *et al.*, 2023; Méar *et al.*, 2023; Jain *et al.*, 2024). Notably, 15% of studies reported only sample size without specifying participant numbers, obscuring how many individuals contributed to the data.

Sufficient sample size is critical for achieving adequate statistical power in omics studies and minimizing the risk of exaggerating effect sizes and drawing incorrect conclusions (Wagner and Kleiner, 2025). In bulk studies, sample sizes ranged from 4 to 180 (Fig. 3D, Supplementary Table S3). For bulk transcriptome studies, six to seven samples per group is considered the minimum threshold, with studies using fewer samples being underpowered (Ching *et al.*, 2014; Baccarella *et al.*, 2018). For bulk proteome analysis, six samples per group has been shown to be adequate (Zhou *et al.*, 2012). For differentially methylated region identification, at least two samples per group are required, provided per-sample coverage is within the recommended range (Ziller *et al.*, 2015).

**Figure 4.**
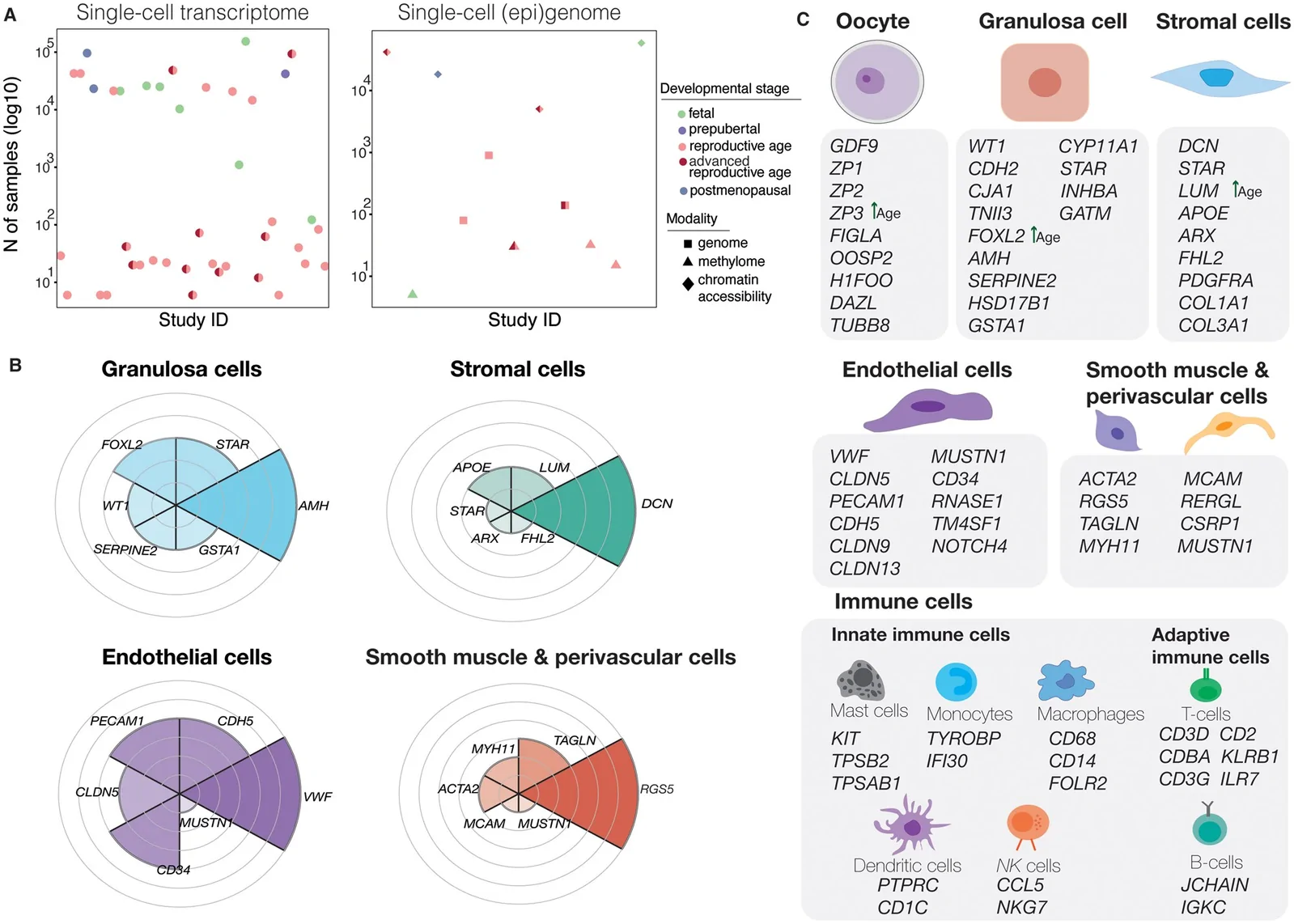
**Overview of single-cell studies included in the review.** (**A**) Scatterplots demonstrate the number of samples in different single-cell studies per omic level analyzed. The *y*-axis is displayed on log_10_ scale to visualize sample sizes spanning several orders of magnitude. (**B**) Radar plots illustrate the frequency distribution of cell type-specific markers across multiple single-cell transcriptomic studies. Radial distances indicate relative frequencies. (**C**) Summary of all markers used for identifying specific cell types across single-cell transcriptomic studies.

Single-cell oocyte studies contained between 6 and 412 samples, with the largest using scChaRM-seq (Yan *et al.*, 2021) (Fig. 4A). Two studies followed with 183 and 140 oocytes’ genomes analyzed (Hou *et al.*, 2013; Ghevaria *et al.*, 2022). Of 15 scRNA-seq studies profiling ovarian tissue cells using the 10X Genomics assay, the largest dataset contained 245 069 cells, collected between 6 and 21 PCW (Garcia-Alonso *et al.*, 2022) (Fig. 4A). The largest adult ovarian dataset comprised 95 979 cells from control and doxorubicin-treated cortex and medulla explants of postmenopausal patients (Devrukhkar *et al.*, 2025). The next largest dataset included 92 965 cells from participants aged 18–49 years, though it was unclear whether this number represented pre- or post-quality control (QC) count (Wu *et al.*, 2024). In 4 out of the 11 studies that reported both pre- and post-QC cell counts, the mean cell count reduction was 14.5% (±18.8 SD). Power analysis for single-cell transcriptome studies is target-dependent, with specific tools available depending on whether the goal is to detect changes in cell type proportions (Davis *et al.*, 2019; Liang *et al.*, 2022) or identify differentially expressed genes (Schmid *et al.*, 2021). For the latter, sequencing depth should be considered, as deeper sequencing provides greater benefit than increased cell numbers (Svensson *et al.*, 2019).

### Bioinformatic analysis

Thorough reporting of omics data analysis steps is crucial for interpreting results and enabling method reuse and secondary data analysis (Rehm *et al.*, 2021; Price *et al.*, 2024), especially given the growing number of computational analysis tools (Luecken *et al.*, 2025). For example, preprocessing strategies and QC criteria can substantially affect downstream results, including differential gene expression (Conesa *et al.*, 2016; Sheng *et al.*, 2017), clustering (Heumos *et al.*, 2023), functional gene enrichment (Paton *et al.*, 2024), principal component analysis (Van Lingen *et al.*, 2024), and differentially methylated region identification (Ziller *et al.*, 2015; Gong *et al.*, 2022). Among the bulk transcriptome studies analyzed, 48% reported their preprocessing methods (Supplementary Table S4). Of the scRNA-seq studies, 42% reported their cell filtering criteria (Supplementary Table S5). Only one of four genome studies reported its data analysis methodology, as did four out of eight methylome studies. In contrast, all chromatin accessibility studies reported their QC strategy (Supplementary Table S6).

Apart from preprocessing, downstream analysis strategies also influence the biological insights derived from data. For example, cell type annotation in single-cell transcriptomics is a core task whose accuracy is crucial for characterizing complex tissues, identifying regulatory patterns and potential therapeutic targets (Wijewardena *et al.*, 2025). All analyzed studies used a clustering-followed-by-annotation approach, in which cell clusters are annotated using marker genes. Immune cell markers varied highly between studies, suggesting the potential value of automated classifiers like CellTypist, which uses pre-trained models from large-scale immune datasets to standardize immune cell annotation (Domínguez Conde *et al.*, 2022). Annotation can also be performed using large language models, obviating the need for reference datasets and potentially improving annotation accuracy and the detection of new cell subtypes (Yang *et al.*, 2022; Hou and Ji, 2024; Wijewardena *et al.*, 2025; Ye *et al.*, 2025).

Spatially resolved omics is a rapidly evolving area, performed in five of the included studies. Each study focused analysis on one of three areas: transcription factor activity in spatially identified cell types, spatial cell-to-cell communication, or spatially variable gene identification (Supplementary Table S7). Despite its rapid development, spatial analysis presents methodological challenges. One of them is cell segmentation, particularly in dense regions or cells with irregular morphology (Alexandrov *et al.*, 2023; Si *et al.*, 2025). Segmentation errors can propagate to downstream analysis, confounding cell proximity and gene expression. Additional challenges arise in spatially aware cell-cell communication analysis, which is entirely data-driven and lacks ground truth validation, with its results depending on the ligand-receptor reference database used and its version (Cesaro *et al.*, 2025). These databases, such as CellPhoneDB, are constantly updated (Troulé *et al.*, 2025). This underscores the importance of reporting the database version used, similar to best practices in proteomics where analyzed studies commonly report the UniProt version, continuously updated every 8 weeks (UniProt Consortium, 2023) (Supplementary Table S8).

### Data availability across studies

Raw data, such as FASTQ files, were fully available without restrictions in 75 studies (62%), partially available in 5 (4%), unavailable in 34 studies (28%), and available upon request in 7 studies through restricted database access (n = 5) or by contacting the authors (n = 2).

### Molecular characterization of the ovary throughout its life cycle

#### Fetal

Sixteen studies analyzed fetal female gonadal tissue collected between 4 and 26 PCW. Five (31%) focused specifically on PGCs and fetal germ cells (FGCs), using these terms interchangeably. The rest examined the developing ovary more broadly (Supplementary Table S2).

Upon colonizing the gonadal ridges around 4–5 PCW, PGCs, initially broadly potent, become restricted to germline progenitors through coordinated action of niche signals that prevent somatic differentiation (Nicholls *et al.*, 2019). All studies agree that until 9.5–11 PCW, female FGCs are predominantly mitotic, expressing *POU5F1* and *NANOG.* Mitotic FGCs share similar transcriptional characteristics (Guo *et al.*, 2015), are confined to the ovarian outer cortex (Garcia-Alonso *et al.*, 2022), and rapidly proliferate to a population of 5–6 million cells (Motta *et al.*, 1997; Ford *et al.*, 2020). At 10 PCW, FGCs exhibit very low DNA methylation levels, indicating a hypomethylated genome, particularly in intergenic regions. By 11 PCW, methylation levels begin to increase, signaling the onset of global remethylation. Around the same period, later-stage FGCs emerge, characterized by decreased expression of naïve-like pluripotency genes and increased expression of *DAZL* and *DDX4*, factors identified in earlier targeted studies as crucial for human germ cell maturation, confirming earlier mouse-based findings (Anderson *et al.*, 2007). About 25% of studies categorize later-stage FGCs into three groups: pre-meiotic retinoic acid (RA)-responsive cells, expressing key mammalian factor *STRA8* for S phase entry and meiotic gene activation (Shimada *et al.*, 2023); meiotic cells, defined by the synaptonemal complex core constituent *SYCP1* (Dunce *et al.*, 2018); and folliculogenesis-phase FGCs, characterized by genes essential for primordial follicle formation, such as *FIGLA* and *ZP3* (Bayne *et al.*, 2004; Gook *et al.*, 2008). Later-stage FGCs migrate inward from the peripheral outer cortex towards the medulla (Li *et al.*, 2017; Garcia-Alonso *et al.*, 2022). The single proteomic study of the human fetal ovary in our review reports increased expression of meiosis-associated proteins (SYCP3, STAG3, REC8, MAEL, TEX11) and enrichment of pathways related to meiotic processes and chromosomal synapsis at 19.5 weeks of development (Bothun *et al.*, 2018), supporting meiotic FGCs predominance in the complex mixtures of germ cells at this stage (Wang *et al.*, 2022). One study also found that meiotic FGCs display an increased ratio of X-chromosome to autosome gene expression, reflecting upregulation of X-linked genes during meiosis entry, potentially essential for oocyte maturation (Chitiashvili *et al.*, 2020).

Supporting somatic cells, originating from the mesonephros and proliferating coelomic epithelium and beginning specification at week 7 of development, are essential for gonad development and folliculogenesis (Wartenberg, 1982; Wamaitha *et al.*, 2023). Granulosa cells are central to follicle formation, as each oocyte must be encapsulated by a sufficient number of them (Bayne *et al.*, 2004; Ford *et al.*, 2020). Evidence suggests that pre-granulosa cells form around 8 PCW in two waves, similar to mice (Niu and Spradling, 2020). One population originates from *GATA4*+ coelomic epithelium-derived early supporting gonadal cells expressing *DMRT1*, *CPA2*, *GRP37*, while another emerges from the same progenitor-derived ovarian surface epithelium (OSE) expressing *LHX2* (Garcia-Alonso *et al.*, 2022). Consistent with this, another study reported *LHX2* expression in both pre-granulosa cells and the OSE itself (McGlacken-Byrne *et al.*, 2025). Both populations express *FOXL2*, but the first additionally expresses steroidogenic factors *HSD17B6* and *CYP19A1*, important for estrogen production. The second population comprises two subpopulations differing in *FOXL2* levels and expression of RA pathway regulators, such as *CYP26B1* and *ALDH1A1*, and the bone morphogenetic protein *BMP2*. BMP2 expression has also been linked to mural granulosa cells around 10 PCW, implicating it in the mitosis-meiosis transition (Li *et al.*, 2017). Spatial transcriptomics analysis shows that these pre-granulosa cells are regionally confined: the first population inhabits the medulla, while the two subpopulations of the second group occupy outer and inner cortex (Garcia-Alonso *et al.*, 2022). Consistently, two molecularly distinct pre-granulosa cell populations have been described: an early population emerging between 6 and 10 PCW, and a later one appearing around 10 PCW (Taelman *et al.*, 2024). Additional candidate progenitors include *TAC1+* co-expressing *SERPINE2* and *GSTA1*, and potentially bipotent *KRT19*+ cells capable of differentiating into both granulosa and theca cells (Wang *et al.*, 2022). About 37% of studies (6 out of 16) found *FOXL2* expression marks pre-granulosa cell emergence, with onset between 6 and 12 PCW (Mamsen *et al.*, 2017; Lecluze *et al.*, 2020). Around 17 PCW, more differentiated flattened granulosa cells appear in the inner cortex, characterized by upregulated NOTCH receptors and their HES/HEY targets (Garcia-Alonso *et al.*, 2022). Additionally, Garcia-Alonso *et al.* (2022) and Li *et al.* (2017) mentioned that during this period, primordial follicles, consisting of oogenesis-phase FGCs enclosed by these granulosa cells, begin to emerge.

About 75% of the studies (12 out of 16) did not characterize other somatic cell types. Two studies described endothelial cells, characterized by *PECAM1* expression (Li *et al.*, 2017; Taelman *et al.*, 2024). One of these studies also identified *RGS5*-expressing smooth muscle cells, *CD53*-expressing immune cells, and *PDGFRA* and *GATA4*-expressing stromal cells (Taelman *et al.*, 2024). Additionally, OSE cells were reported to highly express neural and sympathetic development genes, suggesting a potential role for innervation in both ovarian development and puberty onset (McGlacken-Byrne *et al.*, 2025).

#### (Pre)puberty

We identified five studies (4%) that included (pre)pubertal participants: transcriptomic profiling of child and adult ovarian cortical follicles (Rooda *et al.*, 2024); snRNA-seq and spatial analysis of prepubertal ovarian tissue from fertility preservation patients used as controls in a galactosemia study (Kavarthapu *et al.*, 2024); proteome and matrisome analysis of prepubertal ovarian tissue (Ouni *et al.*, 2022); transcriptomic profiling of pooled oocytes from an 11- and a 21-year-old (Markholt *et al.*, 2012); and cumulus cell analysis comparing pubertal participants and adult oocyte donors (Gokyer *et al.*, 2024) (Supplementary Table S2).

Rooda *et al.* (2024) identified two distinct follicle subpopulations within the ovarian reserve: one with oocytes capable of completing folliculogenesis, and another lacking oocyte markers but potentially supporting the first subtype before degenerating. This pattern resembles the two follicle populations described in mice: the first wave that remodels to enhance androgen production during juvenile development rather than completing oogenesis, and the second wave that supports lifelong fertility (Niu and Spradling, 2020; Yin and Spradling, 2025). However, unlike mice, where these follicular populations are similar in size, humans show greater size disparity between the two groups. The study also uncovered age-related differences in extracellular matrix and theca cells. Kavarthapu *et al.* (2024) mapped major cell populations in prepubertal ovaries and described fibroblast-like, ECM-producing, steroidogenic, and perifollicular stromal cell subclusters, some of which were also described in a separate study of adult ovarian tissue across a wide age range (Wu *et al.*, 2024). The proteomic study revealed stage-specific proteome and matrisome profiles, highlighting proteins involved in elastic remodeling (FBLN5, POSTN, TNXB) and basement membrane assembly (COL4, NID1). These proteins likely relate to follicle growth, which occurs alongside primordial follicle quiescence and survival. The latter processes are linked to COL14A1 expression, anti-apoptotic factors HRNR and TGFBI, and OGN, which stimulates the Wnt/β-catenin pathway and inhibits follicle activation. OGN was also detected among the 10 most abundant proteins across ovarian samples from reproductive age to menopause (Ouni *et al.*, 2019). Follicular atresia, occurring throughout the prepubertal period, may be connected to the mitochondrial damage and aberrant RNA clearance processes, enriched in this study.

Finally, in one of the studies, the analysis of pubertal ovaries was limited to cumulus cells, which showed enrichment of pathways for cellular communication, intracellular transport, and organelle organization, suggesting suboptimal oocyte cytoplasmic maturation in adolescents (Gokyer *et al.*, 2024).

#### Reproductive age

We identified 91 studies involving participants across the reproductive age spectrum: from 18 years of age to the premenopausal period (ages 46–51). Some studies focused on younger women (n = 49, 54%), while (n = 42, 46%) others included wider age range. Notably, only one study (Wu *et al.*, 2024) used the term perimenopause to describe participants in the upper age range, but without applying standard perimenopausal markers such as hormonal fluctuations, menstrual cycle length variability, and vasomotor symptoms (Brinton *et al.*, 2015). Of the analyzed studies, 30% (27 out of 91) examined oocytes, 40% (36 out of 91) granulosa cells, and 36% (33 out of 91) investigated ovarian tissue cells and follicular fluid (Supplementary Table S3).

Over half of oocyte studies (53%) compared germinal vesicle (GV) and MII stage oocytes, with 30% also including MI oocytes. Among these, 55% (15/27) used *in vivo* matured MII oocytes, 26% described *in vitro* matured, and 19% included both (Supplementary Table S3). Three studies investigated DNA methylation dynamics (methylome) during oocyte maturation, while two analyzed both CpG and non-CpG methylation. One study reported a general increase in CHH and CHG methylation (where H represents nucleotide C, T, or A) (Yu *et al.*, 2017), while another found a genome-wide decrease in GCH methylation (at GCA, GCT, and GCC trinucleotides) near TSS (Yan *et al.*, 2021). In contrast, mouse oocytes show strong enrichment of non-CpG methylation in the mCA context, with CA sites preferentially methylated compared with other CHH and CHG contexts (Shirane *et al.*, 2013). Five transcriptomic studies consistently reported a global decrease in gene expression during oocyte maturation (Cornet-Bartolomé *et al.*, 2021; Ntostis *et al.*, 2021; Hu *et al.*, 2022; Takeuchi *et al.*, 2022; Liu *et al.*, 2024), a finding supported by proteomic data (Virant-Klun *et al.*, 2016). Liu *et al.* (2024) further described a lower number of expressed genes during *in vivo* maturation compared to higher transcriptome levels during *in vitro* maturation, likely due to impaired mRNA decay. Despite the agreement on a decrease in the number of expressed genes, the specific genes identified as critical for oocyte maturation varied between studies, likely due to methodological variation. Additionally, human and mouse oocytes differ in their transcriptional programs during maturation, limiting the direct translation between species (Zhao *et al.*, 2020). Three studies linked oocyte maturation to mitochondrial processes (Li *et al.*, 2020; Yu *et al.*, 2020; Zhang *et al.*, 2025). Similarly, Llonch *et al*., 2021 and Hu *et al.* (2022) identified mitochondrial function as a key characteristic of GV oocytes, with Hu *et al.* noting that oxidative phosphorylation (OXPHOS) pathway assembly occurs primarily during this stage. Low or absent levels of complex I subunits in the OXPHOS machinery may contribute to oocyte longevity and primordial follicle survival (Rodríguez-Nuevo *et al.*, 2022). Zhang *et al.* (2025) further reported downregulation of many mitochondria function-linked proteins in MI oocytes compared to GV oocytes, but upregulation of COX6B1, a factor implicated in normal oocyte maturation (Li *et al.*, 2020). Proteasome activity also appears important: it decreased from GV to MII in younger patients (Galatidou *et al.*, 2024) but was upregulated during GV chromatin remodeling, marked by increased expression of the ubiquitin-proteasome enzyme *UBE2C* (Zhang *et al.*, 2024). Two omics studies of cumulus cells highlighted the importance of EGF signaling in oocyte maturation (Cadenas *et al.*, 2025) and reported it to be altered in pubertal samples compared to the older cohort (Gokyer *et al.*, 2024). Despite variability in MII transcriptomes, *DNMT1* and *TUBB8* were consistently reported across four studies (Yu *et al.*, 2020; Yan *et al.*, 2021; Hu *et al.*, 2022; Caniçais *et al.*, 2025). In their study of the MII oocyte translatome, Hu *et al.* (2022) identified high translational efficiency of *Wee2*, consistent with proteomic data showing WEE2 as the predominant MII-stage protein (Virant-Klun *et al.*, 2018; Galatidou *et al.*, 2024).

Sixteen studies examined age-related oocyte changes. Two studies on GV oocytes (Smits *et al.*, 2021; Huang *et al.*, 2023) consistently reported downregulation of cell cycle regulation in older age groups, while seven studies on MII oocytes (Grøndahl *et al.*, 2010; Battaglia *et al.*, 2016; Barragán *et al.*, 2017; Zhang *et al.*, 2020; Yuan *et al.*, 2021; Wu *et al.*, 2022a; Zhang, Yang, *et al.*, 2025) showed no agreement on age-affected genes or pathways (Supplementary Table S3). Interestingly, studies of the oocyte mitochondrial genome demonstrated no increase in mutation frequency with advancing reproductive age (Boucret *et al.*, 2017; Arbeithuber *et al.*, 2025). Evidence from non-sequencing-based omic research, however, strongly supports age-related changes in the nuclear genome. Through chromosome imaging, Gruhn *et al.* (2019) showed that female fertility follows an inverse U-curve, with reduced fertility in teenagers and women over 35, matching aneuploidy patterns in oocytes and embryos. Their research revealed that maternal age primarily determines meiotic chromosome errors through different mechanisms, e.g. MI nondisjunction in young women and cohesion-related errors in advanced age (Gruhn *et al.*, 2019).

Among 36 omics studies of granulosa cells during maturation or aging, several consensus gene sets emerged, including a core set of 236 genes expressed across different developmental stages (Supplementary Table S3) (Dhori *et al.*, 2024). One study combining transcriptomics and epitranscriptomics (mRNA methylation) showed that downregulation of the m6A demethylase FTO increased m6A methylation in granulosa cells, slowing FOS-mRNA degradation in aged ovaries (Jiang *et al.*, 2021). Elevated FOS was also detected in theca cells of early atretic follicles (Fan *et al.*, 2019), and its role in ovarian aging was further confirmed by single-cell transcriptomic analysis (Wu *et al.*, 2024). Three studies (8.3%) examining cumulus cell miRNAomes identified let-7 family miRNAs, particularly let-7f-5p and let-7a-5p, as the most abundant (Velthut-Meikas *et al.*, 2013; Tong *et al.*, 2014; Andrei *et al.*, 2019), findings corroborated in ovarian tissue, suggesting let-7 miRNAs play a regulatory role in folliculogenesis and oogenesis (Xu *et al.*, 2016).

Of the 33 studies on ovarian tissue and follicular fluid, 17 (52%) examined whole ovarian tissue, while the remaining 16 (48%) focused on ovarian follicles or follicular fluid (Supplementary Table S2). The follicular fluid studies included two comparing follicular fluid with matched MII oocytes (Battaglia *et al.*, 2017; Pla *et al.*, 2021), two that analyzed changes in follicular fluid composition during ovulation (Poulsen *et al.*, 2019; Shi *et al.*, 2022), and three studies examining follicular fluid alongside paired granulosa cells (Yoo *et al.*, 2011; Wu *et al.*, 2022a; Cadenas *et al.*, 2023). Three additional studies specifically investigated exosomes, characterizing extracellular vesicle subtypes (Varik *et al.*, 2025), proteome (Liu *et al.*, 2025), and metabolome (Gu *et al.*, 2024). Notably, Liu *et al.* (2025) reported ENO1 deficiency in exosomes from older patients, potentially impairing follicular maturation, a finding consistent with transcriptomic data showing *ENO1* downregulation in aged granulosa cells, which may promote their apoptosis (Zhao *et al.*, 2024).

#### Major ovarian cell types

All studies identified stromal cells as the predominant ovarian cell type. Reported proportions varied: Guahmich *et al.* (2023) found that stromal cells comprise 49.2% of all cells, while Wagner *et al.* (2020) reported a substantially higher proportion (83%). Three studies grouped stroma and theca cells together as a single heterogeneous population (Fan *et al.*, 2019; Guahmich *et al.*, 2023; Wu *et al.*, 2024).

Granulosa cells were identified in four studies (80%), with *AMH* being the most frequently used marker (Fig. 4B). Among studies analyzing follicular single-cell transcriptomes, one characterized all developmental stages (Zhang *et al.*, 2018), while the other focused solely on antral follicles (Fan *et al.*, 2021). Both reported peak androgen receptor (*AR*) expression in granulosa cells during the antral stage, supporting its role as a stage-specific marker (Jeppesen *et al.*, 2012). Both studies also emphasized the importance of NOTCH ligand-receptor signaling during this stage. Zhang *et al.* (2018) reported protein ubiquitination enrichment in preovulatory follicles, a finding supported by the expression of protein ubiquitination markers in granulosa cell clusters at the same stage (Wu *et al.*, 2022b). Both studies confirmed elevated expression of steroidogenic enzyme genes *CYP11A1* and *HSD3B2* in preovulatory follicles.

Among 13 studies comparing age groups, 6 (46%) investigated ovarian aging. Two (33%) highlighted dysregulated glucose metabolism: abnormal glucose metabolism in aged follicles (Diez-Fraile *et al.*, 2014) and elevated glucose and pyruvate in GV and MI oocytes from older women (Smits *et al.*, 2023).

#### Other ovarian cell types

Besides the dominant cell types, all studies mentioned endothelial cells, most commonly marked by *VWF* (Fig. 4B). Other vascular cell types—smooth muscle cells and pericytes—appeared in four (Fan *et al.*, 2019; Guahmich *et al.*, 2023; Wu *et al.*, 2024; Jin *et al.*, 2025) and two studies (Wagner *et al.*, 2020; Jones *et al.*, 2024), respectively. Wagner *et al.* (2020) classified cells expressing both smooth muscle cell and pericyte markers as perivascular cells, while Jones *et al.* (2024) distinguished pericytes as a distinct population by *RGS5*, *NOTCH3*, *ACTA2*, and *MUSTN1.* Only *NOTCH3* overlapped with the perivascular markers reported by Wagner *et al.* (Fig. 4C), and in mouse studies this gene is used as a marker not only for pericytes (Morris *et al.*, 2022) but also stromal subpopulations (Isola *et al.*, 2024). Additionally, Wagner *et al.* (2020) classified *RGS5* and *ACTA2* as stromal markers, with *MUSTN1* absent from their clusters.

Immune cell populations were characterized in four studies (67%). All identified T cells, with three studies using *ILR7* expression alongside study-specific markers. Macrophages were reported in two studies, marked by *CD68* (Fan *et al.*, 2019; Jones *et al.*, 2024). Wagner *et al.* (2020) further categorized non-T cells into tissue-resident and antigen-presenting cells. Both categories potentially include macrophages, since these cells express non-specific receptors for antigenic proteins, HLA-DR, and are primary tissue residents (Gray and Farber, 2022). Fan *et al.* (2019) characterized innate immune cells, a group with both myeloid and lymphoid origin, including monocytes (Gray and Farber, 2022), by *IFI30* expression, which Wu *et al.* (2024) used as a monocyte marker. Natural killer (NK) cells were reported in three studies, identified by *CCL5* and *NKG7* expression in two studies (Fan *et al.*, 2019; Wu *et al.*, 2024), and by *KLRD1*, *TRCD*, and *GNLY* in Jones *et al.* (2024) (Fig. 4C). Wu *et al.* (2022b) noted variable NK presence in preovulatory follicles, identifying them in only two of six samples.

Our understanding of the ovarian single-cell landscape can be confounded by tissue dissociation protocols, which can differ in recovery of specific cell types and shift cellular proportions (Denisenko *et al.*, 2020; Slyper *et al.*, 2020; Jankelow *et al.*, 2025). Tissue dissociation also affects transcriptional cellular profiles by inducing expression of certain genes (van den Brink *et al.*, 2017; Machado *et al.*, 2021). Filtering for this factor, as performed by Fan *et al.* (2019), can yield a more accurate representation of *in vivo* cells. Additionally, current enzymatic dissociation methods may reduce viability and structural integrity, limiting capture of rarer or uncharacterized ovarian cell populations like hilus cells (Bai *et al.*, 2025).

#### Postmenopause

Ten studies (8.2% of all included) examined postmenopausal participants (Yi *et al.*, 2018; Ouni *et al.*, 2019; 2022; Chen *et al.*, 2022; Lengyel *et al.*, 2022; Liu *et al.*, 2023; Méar *et al.*, 2023; Jain *et al.*, 2024; Pei *et al.*, 2024; Devrukhkar *et al.*, 2025). Two studies, Chen *et al.* (2022) and Liu *et al.* (2023), analyzed ovarian bulk transcriptomes from the GTEx database, including individuals up to 79 years. Another eight studies generated original data: four focused on the postmenopausal ovarian proteome (Yi *et al.*, 2018; Ouni *et al.*, 2019, 2022; Méar *et al.*, 2023), one profiled the single-cell transcriptome and chromatin accessibility (Lengyel *et al.*, 2022), one combined single-cell transcriptome and proteome (Devrukhkar *et al.*, 2025), one analyzed the methylome (Jain *et al.*, 2024), and one examined spatial transcriptome (Pei *et al.*, 2024) (Supplementary Table S3). Lengyel *et al.* (2022) reported that postmenopausal ovaries are composed mainly of stromal and granulosa cells with abundant perivascular cells, and that cellular composition shows minor inter-donor variability. The increased proportion of perivascular cells aligns with enrichment of complement and coagulation cascade on the proteomic level (Yi *et al.*, 2018). Immune cells showed enriched transcription factor activity, suggesting active immunological processes. Immune activity is further supported by four studies: Chen *et al.* (2022) observed elevated number of *CD4+* T cells and B cells in individuals over 50; Liu *et al.* (2023) found upregulation of immunoglobulin-related genes such as *IGLV-51*, *IGHG1*, and *IGHV4-34* in women in their fifties; Yi *et al.* (2018) identified immunoglobulin-related proteins such as IGHG4 among the top differentially expressed proteins in postmenopausal ovary and immune response among the most enriched proteomic processes; and Devrukhkar *et al*. (2025) showed that aging stronger activates immune-related pathways in the ovarian cortex compared to the medulla. Taken together, these findings emphasize the prominent role of immune system involvement during ovarian aging.

Two studies discussed autophagy regulation: Liu et al. (2023) identified it among the most enriched pathways, while Ouni *et al.* (2022) suggested potential autophagy inhibition through TSPO and DAP upregulation, consistent with findings in mice showing decreased abundance of autophagy-related proteins in the aged ovary (Harasimov *et al.*, 2024). Regarding cell type specificity of cellular senescence markers, stromal cells showed increased expression of *SERPINE1*, *TIMP1/2*, *IGFBP2/3/4* (Lengyel *et al.*, 2022), while SOD2 and MYH9 were highly expressed in ovarian surface epithelium and endothelia, respectively (Devrukhar *et al.*, 2025). Finally, spatial transcriptomics revealed reduced intercellular interactions in postmenopausal ovaries, particularly between stromal and granulosa cells (Pei *et al.*, 2024).

## Discussion

### Gaps in knowledge of ovary studies

Most ovarian omics studies have focused on reproductive-age participants, with limited prepubertal data: one proteomics study and two analyzing follicular or ovarian tissue transcriptomes. The proteomics study revealed key tissue dynamics, particularly regulation of follicular atresia and survival (Ouni *et al.*, 2022). Follicular transcriptome analysis highlighted interfollicular heterogeneity in ovarian cortical follicles from children and adults (Rooda *et al.*, 2024). Although the biological significance of this heterogeneity remains unclear, these intriguing findings await further validation and detailed characterization of follicular subtypes and their impact on long-term fertility.

Research on ovarian tissue from childhood and pubertal girls is scarce due to extremely limited opportunities to obtain samples from healthy individuals. Reproductive-age tissue typically comes from either infertility treatment or ovarian cortex cryopreservation patients. The latter offers a unique opportunity: while most tissue is preserved for later clinical use, a small portion can be used for research to advance our understanding of age-specific biological processes involved in follicular maintenance, recruitment, and maturation. Increasing use of ovarian cortex cryopreservation in pediatric patients may enable more opportunities for studying ovarian biology from childhood through puberty to adulthood (Fabbri *et al.*, 2012). Future studies should be able to characterize the childhood and pubertal ovary from a multi-omic perspective while addressing ethical challenges in valuable tissue sample collection and consent from young patients and their parents.

Perimenopausal and postmenopausal patients are also largely understudied. Despite fertility decline 5–10 years before menopause, primordial follicles containing normal oocytes can exist in postmenopausal ovaries (Costoff and Mahesh, 1975). Findings from Rooda *et al.* (2024) showing the molecularly distinct follicular subpopulations suggest that interfollicular communication and support are essential for follicles to reach the preovulatory stage. This supports the hypothesis that sufficient follicle density is crucial for effective interfollicular communication. When follicular count drops below this critical threshold, often occurring a decade before menopause, maturation may become impaired. The resulting follicles likely stay dormant in postmenopausal ovaries without neighboring follicles support. Detailed profiling of individual postmenopausal follicles and oocytes is thus needed.

Around menopause and the preceding decade, the ovary loses its dual functions: serving as a follicle reservoir for maturation and ovulation and producing hormones. Understanding the cellular composition and molecular characteristics of the remaining cell types in postmenopausal ovaries becomes crucial to identify potential altered functions. This is particularly relevant because ovarian tumors can originate from various cell types, primarily epithelial cells, but also germ cells, stromal cells, and, rarely, granulosa cells (Li *et al.*, 2022). Given these implications, the cellular composition of postmenopausal ovaries demands more thorough attention. Although we identified seven studies with original data on postmenopausal patients, including two single-cell transcriptome analyses (Supplementary Table S3), additional research is needed to examine spatial cell-to-cell communication, cell type-specific aging patterns, and ovarian cancer risk factors.

Atresia is the predominant follicular fate, making its mechanisms crucial for understanding fertility and reproductive aging. However, studies examining follicular atresia across different molecular layers are scarce, highlighting a significant gap in our knowledge of ovarian biology. Two studies provided cell-type-specific insights: Fan *et al.* (2019) characterized atretic follicles by reduced *CJA1* and *CDH2* expression in granulosa cells and elevated *FOS* expression in theca cells, and Fan *et al.* (2021) linked small antral follicle atresia to CD86+ macrophages and a higher ratio of immune genes *CD68*, *FCER1G*, *C1QA* to global gene expression. Wei *et al.* (2023) classified atretic small follicles into three types: proposing morphological criteria based on granulosa cells, theca cells, and basement membrane. Accumulation of mitochondrial DNA damage may mark apoptotic oocytes targeted for elimination by atresia, potentially during puberty (Arbeithuber *et al.*, 2025). Further research should explore atresia across development and investigate its mechanisms beyond granulosa cell apoptosis (Xi *et al.*, 2025). Additional studies exploring other omic layers in atresia, particularly noncoding regulatory RNAs, could enhance our understanding of follicular cell communication and atresia mechanisms, as they influence follicular fate across species and can be effectively profiled through omics technologies (Zhang *et al.*, 2019).

#### AI/ML in ovary multi-omic studies and key recommendations for future

Previously, we discussed that much of the missing knowledge stems from difficulties obtaining rare and ethically sensitive samples. Fortunately, AI/ML approaches enable insights even from limited samples, important for ovarian research across developmental stages where sample access is limited. IVF has improved access to reproductive-age samples, including follicular fluid, GV and MII oocytes, and granulosa cells, while ovarian cortex cryopreservation in both pediatric and adult patients may provide a unique resource for multi-omics studies. Before such datasets can be used by AI/ML models, they should first be harmonized and standardized, including the uniform correction of batch effects and technical noise, as well as the adoption of consistent, semantically structured metadata and terminology (Russkikh *et al.*, 2020; Batista *et al.*, 2022; Chicco *et al.*, 2023). Integrating data across various omics layers at the single-cell and tissue levels, as well as combining large-scale omics datasets with radiology studies, primarily utilizing ultrasound scan of the ovaries, can enhance our understanding of folliculogenesis, follicular atresia, and ovarian function cessation. This integration can refine cell type definitions and infer novel relationships across data modalities, such as gene regulatory networks (GRNs), and the complex interactions between genes and proteins controlling gene expression in tissue.

**Figure 5.**
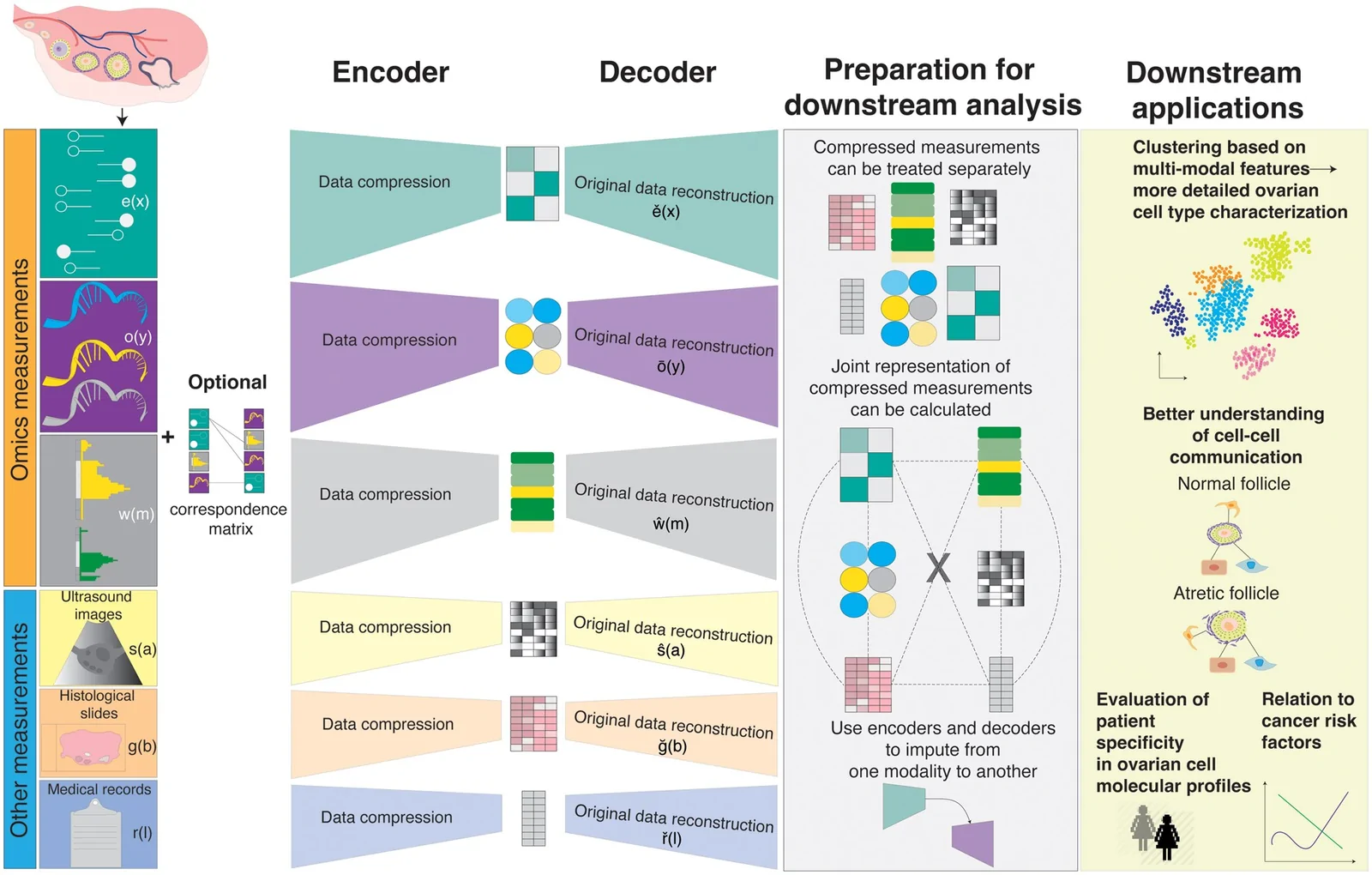
**Structure and potential applications of AI models for multi-omic analysis of ovarian tissue during development and aging.** The figure shows the structure of a general autoencoder model with encoder-decoder pairs. Encoders compress input omic and other measured data into a low-dimensional space. For corresponding samples where omics profiles are measured from the same cell, some autoencoders incorporate a correspondence matrix. Decoders then use this compressed representation to reconstruct the original inputs, accommodating its different distributions. These compressed representations are embedded in a common feature space that considers all modalities. This approach may improve cell-type prediction and cell-to-cell communication analysis, and identification of relationships between omics and phenotypes like age groups. Autoencoders can be also used for imputation of one modality to another.

Deep learning models, particularly autoencoders and transformers, offer powerful frameworks for such integration. Transformers are flexible, general-purpose models that analyze relationships between data elements through an ‘attention’ mechanism, weighing how closely elements relate to each other (Vaswani *et al.*, 2017; Lin *et al.*, 2022). Autoencoders learn to capture the most important characteristics of input data, creating simplified yet meaningful representations while filtering out noise (Chen and Guo, 2023; Berahmand *et al.*, 2024). Typical workflows begin with collecting multi-omics along with imaging data, each with its own scale and characteristics. Preprocessing steps are applied to each data type independently, and then datasets are prepared for integration by feeding them into autoencoder models. These models learn shared latent representations, i.e. abstract features of data, that capture complex relationships across different data types, facilitating tasks such as patient stratification, biomarker discovery, and pathway identification (Fig. 5). The unique properties of autoencoder-based approaches include the ability to jointly integrate heterogeneous information from different modalities and learn from samples with missing modalities (Li *et al.*, 2018). This flexibility allows for imputing missing values, taking properties of the observed data into consideration (Du *et al.*, 2022; He *et al.*, 2024; Baião *et al.*, 2025).

Currently, autoencoder-based approaches are promising because they can handle high-dimensional data from limited sample sizes, accommodate different data distributions, and preserve most of the original signal in low-dimensional representations (Miao *et al.*, 2021; Wen *et al.*, 2023). They enable effective integration of single-cell multi-omic data (Zuo and Chen, 2021; Jeong *et al.*, 2024) and have been applied to ovarian cancer sample classification by integrating various molecular layers (Guo *et al.*, 2020; Hira *et al.*, 2021; Wang *et al.*, 2023). Hatamikia *et al.* (2023) demonstrated that integrating AI models with genomic, proteomic, and metabolomic data improves ovarian cancer diagnostics and risk assessment (Hatamikia *et al.*, 2023). To the best of our knowledge, no studies like those published for ovarian cancer have applied autoencoder-based AI models to analyze multi-omic data from non-cancer and healthy ovaries.

Given the limited human ovarian data, with future multi-modal datasets likely coming from a small number of samples, deep generative decoders like multiDGD (Schuster *et al.*, 2024), offer an alternative for data integration, requiring less training data, making them particularly suitable for smaller datasets. Nonetheless, limited training data size can still compromise model robustness. Therefore, using synthetic data that preserves complex dependencies and interactions within real datasets can be combined with available biological samples to improve performance (Pezoulas *et al.*, 2024; Selvarajoo and Maurer-Stroh, 2024; Reza *et al.*, 2025; Burger, 2026). Additionally, hyperparameter tuning can further improve the accuracy of deep generative models (Brombacher *et al.*, 2022).

Although integrated multi-omics data shows potential for more accurate GRN inference, challenges include unpaired data characteristics and possible dependencies among cells from the same sample (Badia-I-Mompel *et al.*, 2023). Transformer-based foundation models, such as Geneformer, trained on millions of human single-cell transcriptomes across various organs, including the reproductive system (Theodoris *et al.*, 2023; Chen *et al.*, 2024), can improve our fundamental understanding of biological network dynamics. This can facilitate the inference of more accurate GRNs governing the processes of folliculogenesis and follicular atresia, improve annotation of ovarian cell types, as well as conduct *in silico* perturbations evaluating effect of certain genes and pathways on maturation.

A promising strategy for ovarian data integration involves merging multi-omic datasets derived from samples spanning fetal to postmenopausal stages. This approach can provide a comprehensive view of the mechanisms underlying ovarian changes throughout life, as well as responses to treatments and environmental exposures. An AI-based approach has also been applied to follicle identification and counting in ovarian histology sections, potentially helping to select tissue donors and grafts with the highest follicle density to enhance graft longevity for treating premature ovarian insufficiency (Blevins *et al.*, 2024). Thus, the combination of clinical data with AI-driven omics analyses could improve our understanding of treatment efficacy (Bote-Curiel *et al.*, 2022). Furthermore, integrating ovarian omics data with ultrasound scans of ovaries can help to robustly measure follicular density for estimating ovarian reserve. These advanced methods can create predictive frameworks for understanding ovarian development and aging.

However, current AI-based integration methods still have a ‘black-box’ nature. The interpretation of specific genes or regulatory elements deemed important by these models, determining which molecular/omic layers contribute most to the learned integrative features, and exploring how different explainability methods can be combined and domain knowledge incorporated to better understand the outcomes of the model remains an ongoing research area (Toussaint *et al.*, 2023; Wen *et al.*, 2023; Sharma *et al.*, 2024).

With the advancement and democratization of high-throughput technologies and the increasing number of omics datasets, adherence to the Findable, Accessible, Interoperable, Reusable (FAIR) principles (Wilkinson *et al.*, 2016) is crucial for enhancing scientific rigor (Jain *et al.*, 2023; Huang *et al.*, 2025). A key property of FAIR-compliant data resources is efficient metadata organization, which is a cornerstone of global initiatives such as the Human Cell Atlas (HCA) (Regev *et al.*, 2017) and the Human BioMolecular Atlas Program (HuBMAP) (HuBMAP Consortium *et al.*, 2019), as well as data platforms such as CZ CELLxGene Discover (CZI Cell Science Program *et al.*, 2025). Although the set of attributes constituting minimal metadata is subjective and field-specific (Musen *et al.*, 2022; Herr *et al.*, 2023), we propose using extensive metadata for the donor, sample, assay, and computational pipeline. For example, perimenopausal status should be defined using hormone measurements and symptoms following the latest guidelines (Lumsden *et al.*, 2025), and post-mortem interval should be reported for the samples from deceased donors due to its strong impact on ovarian RNA integrity, mitochondrial RNA abundance, and alternative splicing (Ferreira *et al.*, 2018). To enrich metadata and enable secondary analysis, we recommend following omics-specific reporting guidelines, including the HCA and HuBMAP-compliant standards for single-cell (Füllgrabe *et al.*, 2020) and spatial transcriptomics (Jackson and Pachter, 2023), and proteomics (Deutsch *et al.*, 2023). In line with the HCA, we emphasize the importance of sharing detailed sample processing protocols and pre-analysis quality metrics to improve protocol standardization and inform the selection of tailored approaches in future studies (Rozenblatt-Rosen *et al.*, 2021). As inconsistent downstream reporting and the lack of version control are major factors hindering secondary data use (Rehm *et al.*, 2021), we recommend detailed documentation of all analysis steps along with software versions. Data sharing is also crucial, and since ‘data available upon request’ model is often insufficient (Tedersoo *et al.*, 2021), we emphasize the need to explore data anonymization solutions to facilitate broader access. Key recommendations are summarized in Table 1 to guide reporting in future high-throughput ovarian studies. We envision that following them will enhance interpretability and reproducibility of study results, and contribute to the development of a robust knowledge base on ovarian biology across the lifespan of women.

**Table 1.** Key recommendations for reporting standards in future ovarian omics-based studies.

Category	Recommendation
**Study population size**	Studies should report: (a) total number of enrolled participants (b) number of participants in each predefined subgroup (c) number of sequenced participants and those included in the downstream analysis. If applicable, explain any discrepancies between them (e.g. library prep quality, passing quality control filters)
**Sample size**	Power analysis should be conducted *a priori* to determine the appropriate sample size for detecting differences between compared groups or conditions. Use of closely matching pilot data is recommended. (a) For bulk RNA-seq studies, R packages like PROPER and ssizeRNA are recommended, both of which account for sequencing depth. (b) For single-cell RNA-seq studies, in case if DEG analysis is the primary goal, scPS R package which accounts for cell-cell correlation within samples is recommended (Hsu *et al*., 2024). If cell annotation and detection of rare cell types is the primary goal, scPower R package is recommended (Schmid *et al.*, 2021). (c) For spatial transcriptomics studies, *in silico* tissue generation available as a Python command line tool can be used across different technologies to choose tissue sampling strategy and number of samples. Pilot or closely matching data is needed to estimate cell type abundance and adjacency (Baker *et al*., 2023). For 10X Visium technology, the R package PoweREST can be used without pilot data (Shui *et al*., 2025). (d) For multi-omic experiments, if study aims at differential features analysis, R package MultiPower can be used. For building classifiers of biological samples based on multi-omic data, its extension MultiML can be considered (Tarazona *et al*., 2020). Studies should clearly report sample sizes. For single-cell studies, exact cell numbers before and after quality control should be reported.
**Metadata**	Metadata collection should be planned during the study design phase, ensuring study participant confidentiality and protection in compliance with the GDPR, HIPAA, APPI, PIPL, or similar country-specific regulations. Report a minimal set of attributes for: (a) donor, (b) sample, (c) assay(s), (d) computational pipeline. (a) Donor For all donors report age, BMI, ethnicity, chronic diseases and relevant medical history, lifestyle factors affecting fecundity (tobacco smoking, alcohol consumption), and hormonal use (exogeneous hormones like ovarian stimulation drugs and contraceptives, HRT, MHT). For ART, fertility preservation, GRS, elective CS patients, patients undergoing oophorectomy, and living organ donors, additionally report reproductive hormone levels. For deceased organ donors, report cause and circumstance of death, using Hardy’s scale, as employed in the GTEx database. For ART patients, report the ovarian hyperstimulation protocol in detail. For fetal samples, report if the pregnancy termination was due to medical or social reasons, along with the relevant clinical circumstances. For adult study participants, report exact ages. When using the term ‘reproductive age’, clearly report its lower and upper thresholds. Use hormone levels (such as AMH) as criteria for assigning patients to ‘advanced reproductive age’ group. For pubertal study participants, define puberty by hormonal measures (estradiol, LH) and assessment on the PDS (Shirtcliff *et al*., 2009). Report the hormone levels. For perimenopausal study participants, assess and report menstrual cycle regularity, history of vasomotor symptoms, and FSH measurement following the STRAW criteria (Soules *et al*., 2001) and the latest European Society of Endocrinology Clinical Practice Guideline for evaluation and management of menopause and perimenopause (Lumsden *et al.*, 2025). For fetal samples, report age in week units calculated from the fertilization date. For women of reproductive age, include menstrual cycle phase data estimated by hormonal levels, ultrasound, or self-reported cycle day. (b) Sample Report the anatomical sampling site (ovarian cortex, medulla, hilum) or source of isolated follicles or oocytes. Deposit tissue collection and processing protocols (freezing, fixation, or maturation) at protocols.io and report associated DOIs. For deceased donors, report PMI. Report initial handling time, conditions before storage, temperature conditions, and storage duration before final processing. Report sample preparation for assay and single-cell dissociation protocol if applicable. For isolated follicles, report staging method and criteria. For oocytes, report collection and isolation methods. Provide histological and molecular QC metrics (integrity, fragmentation, concentration) for all samples used in omic assays. (c) Assay(s) For all used assays, deposit protocols at protocols.io and report DOIs. If using a sequencing-based assay, report library preparation method (amplification method and version) and sequencing specifics (details about instrument (manufacturer, model), read length, paired/single-end, indexing, barcode specification, read depth) and resultant coverage). For single cell RNA-seq, follow the minSCe guidelines (Füllgrabe *et al.*, 2020). (d) Computational pipeline Describe all processing steps with the respective software versions. Sharing of metadata in .csv/Excel format is recommended.
**Data analysis**	Studies should report comprehensive details of their data analysis pipelines, including all preprocessing steps, quality control criteria and downstream analysis steps. This should cover versions of genome and annotation, batch effect correction and normalization methods, and all other tools used with their versions and parameters (if default parameters were used, it should be noted). To enhance reproducibility and scalability, usage of workflow managers, like Nextflow and Snakemake, should be considered. Publish code in a Git repository, Zenodo or as a Code Ocean capsule.
**AI/ML use**	When integrating multi-omic datasets, researchers should carefully evaluate different integration approaches and consider that some omic layers may provide more relevant information than others. Additionally, following benchmark studies for ML models designed for multi-omic data is crucial to optimize model parameters and prevent overfitting. It is also recommended to use evaluation platforms like Open Problems allowing for new methods to be compared to existing ones.
**Data sharing**	Raw and processed data, along with accompanying metadata, should be shared in public repositories and thoroughly described to enable replication or secondary analysis. For sensitive data like scRNA-seq and scATAC-seq, where genetic variants are not the primary focus, methods such as BAMboozle (Ziegenhain and Sandberg, 2021) can transform the data by removing donor-related SNPs and indels while preserving gene expression signatures and kinetic splicing information. To facilitate transparency and reproducibility, scripts, workflow files, and software environments (e.g. Conda, Docker) should be shared alongside data.

DEG, differentially expressed genes; GDPR, General Data Protection Regulation; HIPAA, Health Insurance Portability and Accountability Act; APPI, Act on the Protection of Personal Information; PIPL, Personal Information Protection Law; BMI, body mass index; HRT, hormone replacement therapy; MHT, menopause hormone therapy; ART, assisted reproductive technologies; CS, caesarean section; GDR, gender reassignment surgery; GTEx, Genotype-Tissue Expression Portal; LH, luteinizing hormone; PDS, Pubertal Development Scale; FSH, follicle-stimulating hormone; DOI, digital object identifier; PMI, post mortem interval; minSCe, minimum information about a single-cell experiment; SNP, single-nucleotide polymorphism.

## Conclusions and future directions

In this review, we provide not only a state-of-the-art overview of ovarian omics research but also offer general recommendations and highlight the potential use of AI/ML approaches for future studies. Specifically, we systematically surveyed the current knowledge on folliculogenesis, oogenesis, and other ovarian cellular and molecular processes that have been characterized using (single-cell) omics assays throughout the lifespan of women.

While single-cell transcriptomics has established important consensus on cell type markers, cellular subtypes require further validation across different conditions through perturbation studies and multimodal datasets, particularly those profiling multiple omic levels from the same cell, which are currently rare in ovarian studies. We highlighted that integrative approaches making use of multi-omic datasets are crucial for better understanding folliculogenesis, follicle atresia, oogenesis, and ovarian aging; and that despite recent advances, most ovarian omics studies use a limited number of participants and samples, restricting their generalizability. Thus, a comprehensive understanding of ovarian biology from fertility through menopause requires access to samples spanning the entire female lifespan, from fetal development to postmenopausal stages. To address this important scarcity, ART and ovarian cortex tissue cryopreservation from pediatric and adult oncology patients have created a unique opportunity for ovarian tissue collection. These advances, nevertheless, require meticulous adherence to complex ethical regulations and careful navigation of challenging ethical dilemmas.

Finally, we demonstrated how the application of AI/ML approaches could (i) substantially improve the quality of (single-cell) multi-omics data analysis, and (ii) facilitate the integration of molecular profiling data with clinical and radiology datasets, enabling a more comprehensive understanding of ovarian biology.

## Supplementary Material

dmag018_Supplementary_Data

## Data Availability

No new data were generated or analyzed in support of this research. Detailed analysis and figure source codes are available at: https://github.com/CellularGenomicMedicine/ovarian_omics_review
