# Supplementary material for: Mapping ovarian cellular and molecular landscape across the lifespan of women: a scoping review
Source: Hum Reprod Update. 2026 Jul 17;32(5):637–62. doi: 10.1093/humupd/dmag018 (PMC13538718; doi:10.1093/humupd/dmag018)
Supplement: Supplementary file 1 [file dmag018_supplementary_data.zip › Supplementary_Data.pdf]

Mapping ovarian cellular and molecular landscape across the lifespan of women: a scoping review

Darina Obukhova[1,2,3], Marina Loid[3,4], Marieke Schor[5], Han G. Brunner[1,6], Andres Salumets[3,7,8*], Masoud Zamani Esteki[1,2,8*]

[1]Department of Clinical Genetics, Maastricht University Medical Centre (MUMC+), Maastricht, The Netherlands

[2]Department of Genetics and Cell Biology, GROW School for Oncology and Reproduction, Maastricht University, Maastricht, The Netherlands

[3]Department of Obstetrics and Gynecology, Institute of Clinical Medicine, University of Tartu, Tartu, Estonia

[4]Faculty of Science, University of Granada, Spain

[5]University Library, Department of Education and Support, Maastricht University, Maastricht, The Netherlands

[6]Department of Human Genetics, Radboud University Medical Center, Nijmegen, The Netherlands

[7]Celvia CC, Tartu, Estonia

[8]Division of Obstetrics and Gynecology, Department of Clinical Science, Intervention and Technology (CLINTEC), Karolinska Institutet, and Karolinka University Hospital, Stockholm, Sweden

*CORRESPONDING AUTHORS

Masoud Zamani Esteki

Department of Clinical Genetics, Maastricht University Medical Centre+

P. Debyelaan 25, 6229 HX Maastricht, The Netherlands

ORCID ID 0000-0003-3909-0050

E-mail: m.zamaniesteki@maastrichtuniversity.nl

Andres Salumets

Division of Obstetrics and Gynecology, Department of Clinical Science, Intervention and Technology, Karolinska Institutet and Karolinska University Hospital

Alfred Nobels Allé 8, SE-141 52 Huddinge, Stockholm, Sweden

ORCID ID 0000-0002-1251-8160

E-mail: andres.salumets@ki.se

Table of Contents

Supplementary Data File S1. Search strategy for used databases

Supplementary Table S1. Table describing excluded studies, along with the reasons for their exclusion.

Supplementary Table S2. Summary of the human ovarian omics-based studies across the female lifespan

Supplementary Table S3. Summary of the key findings from human ovarian omic studies across the female lifespan

Supplementary Table S4. Summary of analytical methods used to analyze bulk transcriptome ovarian data.

Supplementary Table S5. Summary of methods used to analyze single-cell transcriptome ovarian data.

Supplementary Table S6. Summary of methods used to analyze spatial transcriptome ovarian data.

Supplementary Table S7. Summary of methods used to analyze epigenomic ovarian data.

Supplementary Table S8. Summary of methods used to analyze proteome ovarian data

Supplementary Data File S1. Search strategy for used databases

Medline (PubMed)

#1 (“oogenesis” [MeSH Terms] OR "ovary"[MeSH Terms] OR "oocytes"[MeSH Terms] OR "ovarian reserve"[MeSH Terms] OR "oogenesis"[tiab] OR "ovary"[tiab] OR "oocytes"[tiab] OR "ovarian"[tiab])

AND

#2 ("transcriptome"[MeSH Terms] OR "Gene Expression Regulation"[MeSH Terms] OR "Gene Expression Profiling"[MeSH Terms] OR "Genome-Wide Association Study"[MeSH Terms] OR "Proteome"[MeSH Terms] OR "Proteomics"[MeSH Terms] OR "Multiomics"[MeSH Terms] OR "Multiomics"[Title/Abstract] OR "transcriptom*"[Title/Abstract] OR "gene expression"[Title/Abstract] OR "genome-wide"[Title/Abstract] OR "proteom*"[Title/Abstract] OR "aneuploidy"[Title/Abstract]))

NOT

#3 ((("animals"[MeSH Terms] OR "rat"[tiab] OR "rats"[tiab] OR "mouse"[Title/Abstract] OR "mice"[tiab] OR "pig"[tiab] OR "pigs"[tiab] OR “porcine”[tiab] OR "hamster*"[tiab] OR "rodent*"[tiab] OR "bovine"[tiab] OR "cow"[tiab] OR “zebrafish”[tiab] OR “zebrafish”[MeSH Terms] OR “Drosophila”[tiab] OR “sheep”[tiab] OR “horse”[tiab] OR “equine”[tiab]) NOT ("humans"[MeSH Terms] OR "human"[tiab])) OR ("review"[Title] OR "editorial"[Title] OR "review"[Publication Type] OR “systematic review”[Publication Type] OR “technical report”[Publication Type] OR “prospective studies” [MeSH Terms] OR “Meta-Analysis” [Publication Type]) OR ("ovarian neoplasms"[MeSH Major Topic] OR "polycystic ovary syndrome"[MeSH Major Topic] OR “polycystic ovary syndrome”[tiab] OR “endometriosis”[MeSH Major Topic] OR “endometriosis” [tiab] OR “chlamydia”[MeSH Terms] OR "cancer"[Title] OR "carcinoma"[Title] OR "tumor*"[Title] OR "tumour*"[Title]))

Embase (Ovid)

1 exp oocyte development/ or exp ovary/ or exp oocyte/ or exp ovarian reserve/ or (oogenesis or ovary or oocyte* or ovarian).ti,ab,kf.

2 exp transcriptome/ or exp gene expression regulation/ or exp gene expression profiling/ or exp genome-wide association study/ or exp proteome/ or exp proteomics/ or exp multiomics/ or (multiomics or transcriptom* or "gene expression" or "genome-wide" or proteom* or aneuploidy).ti,ab,kf

3 ((exp animal/ or (mouse or mice or rat or rats or pig or pigs or porcine or hamster or rodent or bovine or cow or zebrafish or drosophila or sheep or horse or equine).ti,ab,kf.) not (exp human/ or human.ti,ab,kf.)) or (exp *ovary tumor/ or exp *ovary polycystic disease/ or exp *endometriosis/ or exp *chlamydia/ or (endometriosis or chlamydia or cancer or carcinoma or tumor* ot tumour*).ti.) or ((review or editorial).ti. or (review or abstract).pt.)

4 1 and 2

5 4 not 3

6 limit 5 to dc="20080101-20250830"

Web of Science Core Collection (Clarivate)

1 **TS=(oogenesis OR ovary OR oocyte* OR ovarian)**

2 TS=(multiomics OR transcriptom* OR "gene expression" OR "genome-wide" OR proteom* OR aneuploidy)

3 #1 AND #2

4 (TS=(mouse OR mice OR rat OR rats OR pig OR pigs OR porcine OR hamster* OR rodent* OR murine OR bovine OR cow OR zebrafish OR drosophila OR sheep OR horse* OR equine OR ape OR monkey OR dog OR dogs OR cat OR cats OR bee OR bombyx OR fish* OR insect* OR plant* OR flower*) NOT TI=human) OR TI=(endometriosis OR chlamydia OR cancer OR carcinoma OR tumor* OR tumour*) OR TI=(review OR editorial) OR DT=Review

5 #3 NOT #4

Supplementary Table S1. Table describing excluded studies, along with the reasons for their exclusion.

First Author's Last Name	Publication year	Article Title	Source	Reason(s) for exclusion
Yamashiro	2018	Generation of human oogonia from induced pluripotent stem cells in vitro	Science	Ineligible cell population
Hu	2022	PRC1-mediated epigenetic programming is required to generate the ovarian reserve	Nat. Comm	Ineligible species (mouse)
Yang	2021	Melatonin: Multi-target mechanism against diminished ovarian reserve based on network pharmacology	Front. Endocrinol	Ineligible study design; no original data
Zhang	2020	Insufficiency of melatonin in follicular fluid is a reversible cause for advanced maternal age-related aneuploidy in oocytes	Redox Biol	Ineligible species (mouse)
Can	2020	Comparative analysis of single-cell transcriptomics in human and Zebrafish oocytes	BMC Genomics	Ineligible study design
Jansova	2018	Localization of RNA and translation in the mammalian oocyte and embryo	PLoS One	Ineligible species (mouse)
Zheng-Hui	2020	RNA-seq transcriptome reveals different molecular responses during human and mouse oocyte maturation and fertilization	BMC Genomics	Ineligible study design
Guo	2021	Single-cell transcriptome and cell-specific network analysis reveal the reparative effect of neurotrophin-4 in preantral follicles grown in vivo	Reprod Biol Endocrinol	Ineligible species (mouse)
Mantikou	2016	Evaluation of ribonucleic and amplification protocols for human oocyte transcriptome analysis	Fertil Steril	Ineligible study design (comparison of 5 commercially available mRNA amplification protocols)
Fang	2022	Comparative transcriptome analysis identifies important maternal molecules and associated biological pathways for pig and human mature oocytes	Reprod Domest Anim	Ineligible study design (focus on comparing pig and human oocytes)
Fukuda	2015	Imbalance between the expression dosages of X-chromosome and autosomal genes in mammalian oocytes	Sci Rep	Ineligible species (mouse)

Soleimani	2011	Mechanisms of chemotherapy-induced human ovarian aging: double strand DNA breaks and microvascular compromise	Aging (Albany NY)	Ineligible study design (no investigation of any of the -omes)
Hui-Ling	2016	Novel effects of the cyclooxygenase-2-selective inhibitor NS-398 on IL-1-beta-induced cyclooxygenase-2 and IL-8 expression in human ovarian granulosa cells	Innate Immun	Ineligible study design
Long	2017	SMAD1/5 mediates bone morphogenetic protein2-induced upregulation of BAMBI expression in human granulosa-lutein cells	Cell Signal	Ineligible study design (investigation of the molecular role of one protein, BMP2)
Pors	2020	Effect of sphingosine-1-phosphate on activation of dormant follicles in murine and human ovarian tissue	Mol Human Reprod	Ineligible study design (testing of one agent's effect on *in vitro* follicle activation)
Souček	2018	Presence of growth/differentiation factor-15 cytokine in human follicular fluid, granulosa cells, and oocytes	J Assist Reprod Genet	Ineligible study design
Zhao	2011	Gonadotropin-induced paracrine regulation of human oocyte maturation by BDNF and GDNF secreted by granulosa cells	Hum Reprod	Ineligible study design
Sena	2009	Human MATER localization in specific cell domains of oocytes and follicular cells	RBMO	Ineligible study design
Shen	2020	Versican expression level in cumulus cells is associated with human oocyte developmental competence	Syst Biol Reprod Med	Ineligible study design (relationship between 10 pre-selected genes and oocyte developmental competence)
Titus	2021	Individual-oocyte transcriptomic analysis shows that genotoxic chemotherapy depletes human primordial follicle reserve~in vivo by triggering proapoptotic pathways without growth activation	Sci Rep	Ineligible study design (acute follicle loss after gonadotoxic chemotherapy)
Adriaenssens	2011	The cumulus cell gene expression profile of oocytes with different nuclear maturity and potential for blastocyst formation	J Assist Reprod Genet	Ineligible study design
Zhang	2017	Effects of trehalose vitrification and artificial oocyte activation on the development competence of human immature oocytes	Cryobiology	Ineligible study design (comparison of two different vitrification protocols)
Fesahat	2018	Developmental competence and apoptotic gene expression patterns of mature and immature human oocytes retrieved from controlled ovarian stimulation cycles	Reprod Biol	Ineligible study design (focus on investigating oocytes from stimulated ICSI cycles)

Fragouli	2012	Transcriptomic analysis of follicular cells provides information on the chromosomal status and competence of unfertilized oocytes	Expert Rev Mol Diagn	Ineligible publication type (editorial)
Jääskeläinen	2010	WNT4 is expressed in human fetal and adult ovaries and its signaling contributes to ovarian cell survival	Mol Cel Endocrinol	Ineligible study design
Sylvestre	2013	Evolutionary conservation of the oocyte transcriptome among vertebrates and its implications for understanding human reproductive function	Mol Hum Reprod	Ineligible study design (investigation of cross-species differences from the evolutionary standpoint)
Bayasula	2013	A proteomic analysis of human follicular fluid: comparison between fertilized oocytes and non-fertilized oocytes in the same patient	J Assist Reprod Genet	Ineligible study design
Schall	2021	Essential shared and species-specific features of mammalian oocyte maturation-associated transcriptome changes impacting oocyte physiology	Am J Physiol Cell Physiol	Ineligible study design (investigation of cross-species differences from the evolutionary standpoint)
Bedenk	2022	Recombinant anti-Mullerian hormone in the maturation media improves in vitro maturation of human immature GV oocytes after controlled ovarian hormonal stimulation	Reprod Biol Endocrinol	Ineligible study design (comparison of different *in vitro* maturation protocols)
Cai	2021	Circular DDX10 is associated with ovarian function and assisted reproductive technology outcomes through modulating the proliferation and steroidogenesis of granulosa cells	Aging (Albany NY)	Ineligible study design (investigation of oocyte biomarker in relation to ART outcome)
MacDonald	2023	Characterization of oogonial stem cells in adult mouse ovaries with age and comparison to *in silico* data on human ovarian aging	Stem Cells Dev	Ineligible species (mouse); no original human data produced
de los Santos	2012	Hormonal and molecular characterization of follicular fluid, cumulus cells and oocytes from pre-ovulatory follicles in stimulated and unstimulated cycles	Hum Reprod	Ineligible population
Fuchs Weizman	2019	Triggering method in assisted reproduction alters the cumulus cell transcriptome	RBMO	Ineligible study design (comparison of single and double triggers of ovarian maturation)
Kawamura	2011	Pre-ovulatory LH/hCG surge decreases C-type natriuretic peptide secretion by ovarian granulosa cells to promote meiotic resumption of pre-ovulatory oocytes	Hum Reprod	Ineligible species (mouse)

Sneeringer	2011	High levels of mineralocorticoids in preovulatory follicular fluid could contribute to oocyte development	Fertil Steril	Ineligible study design
Deng	2023	Growth hormone promotes oocyte maturation in vitro by protecting mitochondrial function and reducing apoptosis	Reprod Sci	Ineligible study design (influence of one component on *in vitro* maturation)
Cui	2018	Endothelin-1 promotes human germinal vesicle-stage oocyte maturation by downregulating connexin-26 expression in cumulus cells	Mol Hum Reprod	Ineligible study design (influence of one component on *in vitro* maturation)
Lu	2013	Involvement of the serine protease inhibitor, SERPINE2, and the urokinase plasminogen activator in cumulus expansion and oocyte maturation	PLoS One	Ineligible species (mouse)
Zhao	2021	Single-cell RNA sequencing reveals species-specific time spans of cell cycle transitions in early oogenesis	Hum Mol Genet	Ineligible study design (cross-species comparison); no original human data
Xiao	2014	MiR-133b regulates the expression of the Actin protein TAGLN2 during oocyte growth and maturation: a potential target for infertility therapy	PLoS One	Ineligible species (mouse)
Liu	2017	Estrogen receptors in granulosa cells govern meiotic resumption of pre-ovulatory oocytes in mammals	Cell Death Dis	Ineligible species (mouse)
Liu	2021	Prediction of oocyte quality using mRNA transcripts screened by RNA sequencing of human granulosa cells	RBMO	Ineligible study design (link between oocyte physiology and blastocyst development)
Lee	2021	Single-cell RNA sequencing identifies molecular targets associated with poor in vitro maturation performance of oocytes collected from ovarian stimulation	Hum Reprod	Ineligible cell population (rescue IVM oocytes)
Capalbo	2022	Maternal exome analysis for the diagnosis of oocyte maturation defects and early embryonic developmental arrest	Reprod Biomed Online	Ineligible study population (women with recurrent oocyte maturation defects and/or preimplantation embryo lethality)
Di Pietro	2008	Expression analysis of TFIID in single human oocytes: new potential molecular markers of oocyte quality	Reprod Biomed Online	Ineligible study design
Xu	2020	Low KLOTHO level related to aging is associated with diminished ovarian reserve	Fertil Steril	Ineligible study design

Li	2015	Correlation of cumulus gene expression of GJA1, PRSS35, PTX3, and SERPINE2 with oocyte maturation, fertilization, and embryo development	Reprod Biol Endocrinol	Ineligible study design
Christou-Kent	2018	PATL2 is a key actor of oocyte maturation whose invalidation causes infertility in women and mice	EMBO Mol Med	Ineligible species (mouse)
Wei	2021	miR-221-3p regulates apoptosis of ovarian granulosa cells via targeting FOXO1 in older women with diminished ovarian reserve (DOR)	Mol Reprod Dev	Ineligible population (older women with DOR, younger women with NOR)
Margulis	2021	Bone morphogenetic protein 15 expression in human ovaries from fetuses, girls, and women	Fertil Steril	Ineligible study design
Fellmeth	2015	Expression and characterization of three Aurora kinase C splice variants found in human oocytes	Mol Hum Reprod	Ineligible species (mouse)
Shimizu	2019	Molecular mechanism of FSHR expression induced by BMP15 in human granulosa cells	J Assist Reprod Genet	Ineligible study design
Lv	2022	Identification and Validation of Autophagy-Related Genes in Primary Ovarian Insufficiency by Gene Expression Profile and Bioinformatic Analysis	Anal Cell Pathol (Amst)	Ineligible cell population (granulosa cell line SVOG)
Yung	2014	Localization of luteinizing hormone receptor protein in the human ovary	Mol Hum Reprod	Ineligible study design
Esf	2021	Oocyte-secreted factors strongly stimulate sFRP4 expression in human cumulus cells	Mol Hum Reprod	Ineligible study design
Yao	2019	Transcriptional profiling of long noncoding RNAs and their target transcripts in ovarian cortical tissues from women with normal menstrual cycles and primary ovarian insufficiency	Mol Reprod Dev	Ineligible population (POI patients)
Nguyen	2019	Effect of the neuropeptide phoenixin and its receptor GPR173 during folliculogenesis	Reproduction	Ineligible study design
Georgiou	2009	Retrotransposon RNA expression and evidence for retrotransposition events in human oocytes	Hum Mol Genet	Ineligible study design
MacDonald	2019	Extracellular matrix signaling activates differentiation of adult ovary-derived oogonial stem cells in a species-specific manner	Fertil Steril	Ineligible study design
Shi	2009	Bone morphogenetic protein-6 stimulates gene expression of follicle-stimulating hormone receptor, inhibin/activin beta subunits and anti-Mullerian hormone in human granulosa cells	Fertil Steril	Ineligible study design
Biase	2017	Oocyte Developmental Competence: Insights from Cross-species differential gene expression and human oocyte-specific functional gene networks	OMICS	Ineligible study design; no original human data

Bastian	2016	Regulation of fibrillins and modulators of TGF-beta in fetal bovine and human ovaries	Reproduction	Ineligible study design; ineligible cell population
Bar-Joseph	2016	Pigment epithelium-derived factor regulation by human chorionic gonadotropin in granulosa cells	Reproduction	Ineligible study design
Hannon	2018	Ovulatory induction of SCG2 in human, nonhuman primate and rodent granulosa cells stimulates ovarian angiogenesis	Endocrinology	Ineligible study design
Li	2014	Increased GDF9 and BMP15 mRNA levels in cumulus granulosa cells correlate with oocyte maturation, fertilization, and embryo quality in humans	Reprod Biol Endocrinol	Ineligible study design
Farhi	2011	Neurotrophin 4 enhances in vitro follicular assembly in human fetal ovaries	Fertil Steril	Ineligible study design
Prasad	2008	Mos 3' UTR regulatory differences underlie species-specific temporal patterns of Mos mRNA cytoplasmic polyadenylation and translational recruitment during oocyte maturation	Mol Reprod Dev	Ineligible study design
Magli	2012	Five chromosome segregation in polar bodies and the corresponding oocyte	RBMO	Ineligible method (no sequencing involved)
Rizzolio	2009	Epigenetic analysis of the critical region I for premature ovarian failure: demonstration of a highly heterochromatic domain on the long arm of the mammalian X chromosome	J Med Genet	Ineligible cell population (lymphoblastoid cell lines)
Koh	2009	STIM1 regulates store-operated Ca2+ entry in oocytes	Dev Biol	Ineligible species (pig)
Garcia-Cruz	2009	ATR, BRCA1 and gammaH2AX localize to synapse chromosomes at the pachytene stage in human oocytes	RBMO	Ineligible study design
Chen	2017	A recurrent missense mutation in ZP3 causes empty follicle syndrome and female infertility	Am J Hum Genet	Ineligible study population
Poulain	2014	Involvement of doublesex and mab-3-related transcription factors in human female germ cell development demonstrated by xenograft and interference RNA strategies	Mol Hum Reprod	Ineligible study design
Vos	2014	Immunohistochemical expression of MMP-14 and MMP-2 activity during human ovarian follicular development	Reprod Biol Endocrinol	Ineligible study design
Gabbay-Benziv	2012	Vasoactive intestinal peptide and its receptors in human ovarian cortical follicles	PLoS One	Ineligible study design
Abir	2009	Keratinocyte growth factor and its receptor in human ovaries from fetuses, girls and women	Mol Hum Reprod	Ineligible study design

Yang	2012	Differentially expressed plasma microRNAs in premature ovarian failure patients and the potential regulatory function of mir-23a in granulosa cell apoptosis	Reproduction	Ineligible population
Grondahl	2012	Specific genes are expressed between cumulus and granulosa cells from individual human pre-ovulatory follicles	Mol Hum Reprod	Ineligible study design
El-Khairi	2012	Analysis of Lin28A in early human ovary development as a candidate gene for primary ovarian insufficiency	Mol Cell Endocrinol	Ineligible study design
Akiyama	2012	The localization and regulation of proprotein convertase subtilisin/kexin PCSK6 in human ovary	Am J Reprod Immunol	Ineligible study design
Sun	2017	XCI-escaping gene KDM5C contributes to ovarian development via downregulating mir-320a	Hum Genet	Ineligible population
Dang	2018	MicroRNA-379-5p is associate with biochemical premature ovarian insufficiency through PARP1 and XRCC6	Cell Death Dis	Ineligible population
Fonseca	2014	BMP15 c.-9C>G promoter sequence variant may contribute to the cause of non-syndromic premature ovarian failure	RBMO	Ineligible population
Jeppesen	2012	LH-receptor gene expression in human granulosa and cumulus cells from antral and preovulatory follicles	J Clin Endocrinol Metab	Ineligible study design
Fu	2020	Anti-Mullerian hormone regulates stem cell factor via cAMP/PKA signaling pathway in human granulosa cells by inhibiting the phosphorylation of CREB	Reprod Sci	Ineligible study design
Zhang	2019	MicroRNA-181a promotes follicular granulosa cell apoptosis via sphingosine-1-phosphate receptor 1 expression downregulation	Biol Reprod	Ineligible study design
Detti	2018	Anti-Mullerian hormone AMH may stall ovarian cortex function through modulation of hormone receptors other than the AMH receptor	Reprod Sci	Ineligible study design
Li	2020	miR-21 and pellino-1 expression profiling in autoimmune premature ovarian insufficiency	J Immunol Res	Ineligible population
Escoffier	2016	Homozygous mutation of PLCZ1 leads to defective human oocyte activation and infertility that is not rescued by WW-binding protein PAWP	Hum Mol Genet	Ineligible population

Shaw	2015	Compensatory increase in ovarian aromatase in older regularly cycling women	J Clin Endocrinol Metab	Ineligible study design
Perrett	2008	The early human germ cell lineage does not express SOX2 during in vivo development or upon in vitro culture	Biol Reprod	Ineligible study design
Wang	2020	Long noncoding RNA HCP5 participates in premature ovarian insufficiency by transcriptionally regulating MSH5 and DNA damage repair via YB1	Nucleic Acids Res	Ineligible population
Li	2010	The orphan nuclear receptor NR4A1 regulates transcription of key steroidogenic enzymes in ovarian theca cells	Mol Cell Endocrinol	Ineligible study design
Chang	2015	Recombinant BMP4 and BMP7 increase activin A production by upregulating inhibin beta-A subunit and furin expression in human granulosa-lutein cells	J Clin Endocrinol Metab	Ineligible study design
Jaroudi	2013	Microarray-CGH for the assessment of aneuploidy in human polar bodies and oocytes	Methods Mol Biol	Ineligible publication type
Knauff	2009	Genome-wide association study in premature ovarian failure patients suggests ADAMTS19 as a possible candidate gene	Hum Reprod	Ineligible population
Banz	2010	Bridge-1 is expressed in human granulosa cells and is involved in the activin A signaling cascade	Fertil Steril	Ineligible study design
He	2013	FSH modulates the expression of inhibin-alpha and the secretion of inhibins via orphan nuclear receptor NUR77 in ovarian granulosa cells	Mol Reprod Dev	Ineligible study design
Pyun	2012	LAMC1 gene is associated with premature ovarian failure	Maturitas	Ineligible population
Wang	2021	Hypermethylation-mediated downregulation of lncRNA RVT1 promotes granulosa cell apoptosis in premature ovarian insufficiency via interacting with Foxo3a	J Cell Physiol	Ineligible population
Bertoldo	2019	Non-canonical AMP SMAD1/5/8 signaling in human granulosa cells	Mol Cell Endocrinol	Ineligible study design
Ben-Meir	2015	Coenzyme Q-dependent mitochondrial respiratory chain activity in granulosa cells is reduced with aging	Fertil Steril	Ineligible study design
Wang	2014	PCSK6 regulated by LH inhibits the apoptosis of human granulosa cells via activin A and TGF-beta-2	J Endocrinol	Ineligible study design

Mamsen	2020	Expression of the insulin-like growth factor system in first- and second-trimester human embryonic and fetal gonads	J Clin Endocrinol Metab	Ineligible study design
Martins	2022	The role of macrophages phenotypes in the activation of resolution pathways within human granulosa cells	Reprod Biol Endocrinol	Ineligible study design
Chen	2015	Transforming growth factor beta-1 upregulates connexin43 expression in human granulosa cells	Hum Reprod	Ineligible study design
Huang	2009	Effects of growth differentiation factor 9 on cell cycle regulators and ERK42/44 in human granulosa cell proliferation	Am J Physiol Endocrinol Metab	Ineligible study design
Chen	2020	ALK4-SMAD3/4 mediates the effects of activin A on the upregulation of PAI-1 in human granulosa lutein cells	Mol Cel Endocrinol	Ineligible study design
Ogura-Nose	2012	Anti-Mullerian hormone (AMH) is induced by bone morphogenetic protein (BMP) cytokines in human granulosa cells	Eur J Obstet Gynecol Reprod Biol	Ineligible study design
Cheng	2015	TGF-beta1 Upregulates connective tissue growth factor expression in human granulosa cells through Smad and ERK1/2 Signaling Pathways	PLoS One	Ineligible study design
Thanatsis	2019	Reduced Foxo3a, FoxL2, and p27 mRNA expression in human ovarian tissue in premature ovarian insufficiency	Hormones (Athens)	Ineligible population
Liu	2022	Identification of Biomarkers for Predicting Ovarian Reserve of Primordial Follicle via Transcriptomic Analysis	Front Genet	Ineligible study design
Yang	2022	FAM111A is a novel molecular marker for oocyte aging	Biomedicines	Ineligible study design
Li	2019	Growth Hormone Promotes in vitro maturation of Human Oocytes	Front Endocrinol (Lausanne)	Ineligible study design
Brkazert	2019	Human Ovarian Granulosa Cells Isolated during an IVF Procedure Exhibit Differential Expression of Genes Regulating Cell Division and Mitotic Spindle Formation	J Clin Med	Ineligible study design
Choi	2017	Coordinated Regulation Among Progesterone, Prostaglandins, and EGF-Like Factors in Human Ovulatory Follicles	J Clin Endocrinol Metab	Ineligible study design
Liu	2019	ALK4-SMAD2/3-SMAD4 signaling mediates the activin A-induced suppression of PTX3 in human granulosa-lutein cells	Mol Cell Endocrinol	Ineligible study design

Chang	2013	BMP15 suppresses progesterone production by down-regulating StAR via ALK3 in human granulosa cells	Mol Endocrinol	Ineligible study design
Mase	2012	MiR-21 is enriched in the RNA-induced silencing complex and targets COL4A1 in human granulosa cell lines	Reprod Sci	Ineligible cell population
Herr	2013	Human chorionic gonadotropin controls luteal vascular permeability via vascular endothelial growth factor by down-regulation of a cascade of adhesion proteins	Fertil Steril	Ineligible study design
Convissar	2017	Regulation of AMH by oocyte-specific growth factors in human primary cumulus cells	Reproduction	Ineligible study design
Eilso Nielsen	2010	Concentrations of anti-Mullerian hormone in fluid from small antral follicles show a negative correlation with CYP19 mRNA expression in the corresponding granulosa cells	Mol Hum Reprod	Ineligible study design
Fang	2015	Growth differentiation factor-8 decreases STAR expression through ALK5-mediated SMAD3 and ERK1/2 signaling pathways in luteinized human granulosa cells	Endocrinology	Ineligible study design
Tripodo	2023	Pseudotemporal ordering of spatial lymphoid tissue microenvironment profiles trails Unclassified DLBCL at the periphery of the follicle	Front. Immunol.	Ineligible population; ineligible cell population
Harry	2023	Maternal patterns of inheritance alter transcript expression in eggs	BMC Genomics	Ineligible species
Wei	2023	PGC7 regulates genome-wide DNA methylation by regulating ERK-mediated subcellular localization of DNMT1	IJMS	Ineligible cell population
Qin	2012	ESR1, HK3 and BRSK1 gene variants are associated with both age at natural menopause and premature ovarian failure	Orphanet J Rare Dis	Ineligible population
Di-Battista	2023	Premature ovarian insufficiency is associated with global alterations in the regulatory landscape and gene expression in balanced X-autosome translocations	Epigenetics & Chromatin	Ineligible population
Ferreira	2023	Aging and oocyte competence: A molecular cell perspective	WIREs Mechanisms of Disease	Ineligible publication type

Liu	2023	Detection of the effect of microvibrational stimulation on human discarded immature oocytes by single-cell transcriptome sequencing technology	J Assist Reprod Genet	Ineligible study design
Marton	2023	Comparative analysis of transcriptomic changes including mRNA and microRNA expression induced by the xenoestrogens zearalenone and bisphenol A in human ovarian cells	Toxins	Ineligible study design
Zhao	2023	Comprehensive analysis of untargeted metabolomics and lipidomics in girls with central precocious puberty	Front. Pediatr.	Ineligible cell population
Zgorecka	2022	Human ovarian follicular granulosa cells isolated during ART procedure reflect substantial changes in activation of hormonal signaling pathways, during long-term in vitro conditions	Medical Journal of Cell Biology	Ineligible study design
Tucker	2022	Premature Ovarian Insufficiency in CLPB Deficiency: Transcriptomic, Proteomic and Phenotypic Insights	The Journal of Clinical Endocrinology & Metabolism	Ineligible population
Omrizadeh	2022	Altered Expression of GDF9 and BMP15 Genes in Granulosa Cells of Diminished Ovarian Reserve Patients: A Case-Control Study	Cell J	Ineligible population
Ghezelayagh	2018	The Impact of Genetic Variation and Gene Expression Level of The Follicle-Stimulating Hormone Receptor on Ovarian Reserve	Cell J	Ineligible population
Li	2023	TGF-beta1 suppresses de novo cholesterol biosynthesis in granulosa-lutein cells by down-regulating DHCR24 expression via the GSK-3-beta/EZH2/H3K27me3 signaling pathway	Int.J.Biol. Macromol.	Ineligible study design
Devos	2023	Impact of first chemotherapy exposure on follicle activation and survival in human cryopreserved ovarian tissue	Hum Reprod	Ineligible study design
Devic	2019	Genes for anti-Mullerian hormone and androgen receptor are underexpressed in human cumulus cells surrounding morphologically highly graded oocytes	SAGE Open Medicine	Ineligible study design
Poretsky	2023	The effects of irisin and leptin on steroidogenic enzyme gene expression in human granulosa cells: In vitro studies	Metabolism Open	Ineligible study design

Zhang	2022	Study of differential proteomics in granulosa cells of premature ovarian insufficiency (POI) and the roles and mechanisms of RAC1 in granulosa cells	Molecular and Cellular Endocrinology	Ineligible population
Ramirez	2022	Changes in IL-16 Expression in the Ovary during Aging and Its Potential Consequences to Ovarian Pathology	Journal of Immunology Research	Ineligible study design
Adhikari	2022	Oocyte mitochondria – key regulators of oocyte function and potential therapeutic targets for improving fertility	Biology of Reproduction	Ineligible publication type
Babayev	2022	Age-associated changes in cumulus cells and follicular fluid: the local oocyte microenvironment as a determinant of gamete quality	Biology of Reproduction	Ineligible publication type
Buratini	2022	The putative roles of FSH and AMH in the regulation of oocyte developmental competence: from fertility prognosis to mechanisms underlying age-related subfertility	Hum Reprod Update	Ineligible publication type
Li	2021	An integrated RNA-Seq and network study reveals that valproate inhibited progesterone production in human granulosa cells	J. Steroid Biochem. Mol.Bio.	Ineligible study design
Carter	2021	Transcriptional heterogeneity of stemness phenotypes in the ovarian epithelium	Commun Biol	Ineligible species
Kawano	2021	Chromosomal analyses of human giant diploid oocytes by next-generation sequencing	Reprod Medicine & Biology	Ineligible cell population
Candi	2021	Mechanisms of quality control differ in male and female germ cells	Cell Death Differ	Ineligible publication type
Sperduti	2021	Quantification of hormone membrane receptor FSHR, GPER and LHCGR transcripts in human primary granulosa lutein cells by real-time quantitative PCR and digital droplet PCR	Gene Reports	Ineligible study design
Wan	2021	Effect of luteinizing hormone concentration on transcriptome and subcellular organelle phenotype of ovarian granulosa cells	J Assist Reprod Genet	Ineligible study design
La	2021	Insights from the Applications of Single-Cell Transcriptomic Analysis in Germ Cell Development and Reproductive Medicine	IJMS	Ineligible publication type
Wartosch	2021	Origins and mechanisms leading to aneuploidy in human eggs	Prenatal Diagnosis	Ineligible publication type

Klobučar	2020	Mass spectrometry-based glycomic profiling of the total IgG and total proteome N-glycomes isolated from follicular fluid	Biomolecular Concepts	Ineligible study design
Lunding	2020	Hippo signaling, actin polymerization, and follicle activation in fragmented human ovarian cortex	Molecular Reproduction Devel	Ineligible study design
Brązert	2020	Expression of genes involved in neurogenesis, and neuronal precursor cell proliferation and development: Novel pathways of human ovarian granulosa cell differentiation and transdifferentiation capability in vitro	Mol Med Report	Ineligible study design
Xiong	2020	Evaluation of CD4+CD25+FOXP3+ regulatory T cells and FOXP3 mRNA in premature ovarian insufficiency	Climacteric	Ineligible population
Zhou	2020	Expression profiles of circular RNA in granulosa cells from women with biochemical premature ovarian insufficiency	Epigenomics	Ineligible population
Brązert	2019	Genes involved in hormone metabolism and cellular response in human ovarian granulosa cells	J Biol Regul Homeost Agents	Ineligible study design
Conrad	2017	Single-Cell Expression Profiling and Proteomics of Primordial Germ Cells, Spermatogonial Stem Cells, Adult Germ Stem Cells, and Oocytes - Stem Cells: Biology and Engineering	Stem Cells: Biology and Engineering (book)	Ineligible publication type
Dalman	2018	Establishment and characterization of human theca stem cells and their differentiation into theca progenitor cells	J of Cellular Biochemistry	Ineligible study design
Stringer	2018	The capacity of oocytes for DNA repair	Cell. Mol. Life Sci.	Ineligible publication type
Clarke	2018	Regulation of germ cell development by intercellular signaling in the mammalian ovarian follicle	WIREs Developmental Biology	Ineligible publication type
Ruebel	2017	Obesity Modulates Inflammation and Lipid Metabolism Oocyte Gene Expression: A Single-Cell Transcriptome Perspective	The Journal of Clinical Endocrinology & Metabolism	Ineligible study design
Rosario	2017	RNA-binding proteins in human oogenesis: Balancing differentiation and self-renewal in the female fetal germline	Stem Cell Research	Ineligible publication type

Casarini	2017	Estrogen Modulates Specific Life and Death Signals Induced by LH and hCG in Human Primary Granulosa Cells In Vitro	Int J Mol Sci	Ineligible study design
Cho	2017	Single nucleotide polymorphisms at miR-146a/196a2 and their primary ovarian insufficiency-related target gene regulation in granulosa cells	PLoS One	Ineligible study design
Pasquariello	2017	Micro-RNA sequencing of individual human oocytes	Fertil Steril	Ineligible publication type
Baumgarten	2017	Folliclestimulating hormone (FSH) regulates cytoskeletal protein expression and cell structure during human granulosa cell differentiation	Fertil Steril	Ineligible publication type
Pertynska-Marczewska	2017	Aging ovary and the role for advanced glycation end products	Menopause	Ineligible publication type
Pashaiasi	2016	Identification of the key regulating genes of diminished ovarian reserve (DOR) by network and gene ontology analysis	Mol Biol Rep	Ineligible publication type
Sousa Martins	2016	DAZL and CPEB1 regulate mRNA translation synergistically during oocyte maturation	J Cell Sci	Ineligible species
Albizua	2015	Association between telomere length and chromosome 21 nondisjunction in the oocyte	Hum Genet	Ineligible study design
Merhi	2015	Adiposity Alters Genes Important in Inflammation and Cell Cycle Division in Human Cumulus Granulosa Cell	Reprod.Sci	Ineligible study design
Vassena	2015	The non-coding transcriptome of individual human oocytes is influenced by age and ovarian reserve	Fertil Steril	Ineligible publication type
Woo Seo	2015	Zwint-1 is required for spindle assembly checkpoint function and kinetochore-microtubule attachment during oocyte meiosis	Sci Rep	Ineligible species
Riris	2014	Digital multiplexed mRNA analysis of functionally important genes in single human oocytes and correlation of changes in transcript levels with oocyte protein expression	Fertil Steril	Ineligible study design
Stimpfel	2014	Putative mesenchymal stem cells isolated from adult human ovaries	J Assist Reprod Genet	Ineligible study design
Loreti	2013	The glycan structure in recombinant human FSH affects endocrine activity and global gene expression in human granulosa cells	Mol Cell Endorinol	Ineligible study design

Rossi	2013	Transcriptional control of KIT gene expression during germ cell development	Int. J. Dev. Biol.	Ineligible publication type
McGinnis	2013	Signaling Modalities During Oogenesis in Mammals	Curr Top Dev Biol.	Ineligible publication type
Jin	2012	Novel Functional MAR Elements of Double Minute Chromosomes in Human Ovarian Cells Capable of Enhancing Gene Expression	PLoS One	Ineligible cell population
Noggle	2011	Human oocytes reprogram somatic cells to a pluripotent state	Nature	Ineligible study design
Fragouli	2011	Chromosome Abnormalities in the Human Oocyte	Cytogenet Genome Res	Ineligible publication type
Martin	2008	Meiotic errors in human oogenesis and spermatogenesis	RBMO	Ineligible publication type
Van Den Driesche	2008	Expression and regulation of oestrogen receptors in the human corpus luteum	Reproduction	Ineligible study design
Bouazzi	2019	The transcriptional regulator CBX2 and ovarian function: A whole genome and whole transcriptome approach	Sci Rep	Ineligible cell population
Bukovsky	2009	Immune physiology and oogenesis in fetal and adult humans, ovarian infertility, and totipotency of adult ovarian stem cells	Birth Defects Research	Ineligible publication type
Saadeh	2014	Protection of CpG islands against de novo DNA methylation during oogenesis is associated with the recognition site of E2f1 and E2f2	Epigenetics Chromatin	Ineligible species
Wang	2014	Genome-wide DNA methylation and gene expression patterns provide insight into polycystic ovary syndrome development	Oncotarget	Ineligible population
Katz-Jaffe	2022	In vivo age-related changes in the ovary coincide with widespread repercussions on gene expression unique to the specific ovarian cell population	Fertil & Steril	Ineligible publication type
Duncan	2018	Mammalian oogenesis and female reproductive aging	Aging (Albany NY)	Ineligible publication type
Owens	2019	Gene Expression in Granulosa Cells From Small Antral Follicles From Women With or Without Polycystic Ovaries	J.Clin. Endocrinol. Metab.	Ineligible population
Hanna	2022	Loss of histone methyltransferase SETD1B in oogenesis results in the redistribution of genomic histone 3 lysine 4 trimethylation	Nucleic Acids Research	Ineligible species

Li	2018	Vitamin B2 enhances development of puberty ovaries via regulation of essential elements and plasma endocrine hormones	Trop. J. Pharm Res	Ineligible species; ineligible study design
Osman	2018	Oocyte and embryo manipulation and epigenetics	Semin Reprod Med	Ineligible publication type
Leahy	2021	Inferring simple but precise quantitative models of human oocyte and early embryo development	J.R.Soc. Interface	Ineligible study design
Cadenas	2022	Validating reference gene expression stability in human ovarian follicles, oocytes, cumulus cells, ovarian medulla and ovarian cortex tissue	IJMS	Ineligible study design
May-Panloup	2016	Ovarian ageing: the role of mitochondria in oocytes and follicles	Hum. Reprod. Update	Ineligible publication type
Zheng	2010	Prediciting Meiotic Pathways in Human Fetal Oogenesis	Biology of Reproduction	Ineligible study design
Zhu	2018	Role of RAB5A in FSHR-mediated signal transduction in human granulosa cells	Reproduction	Ineligible population
Chen	2016	Expression pattern of circadian genes and steroidogenesis-related genes after testosterone stimulation in the human ovary	J Ovarian Res	Ineligible study design
Rajkovic	2017	Ovary as a Biomarker of Health and Longevity: Insights from Genetics	Semin Reprod Med	Ineligible publication type
Nousch	2019	Stage-specific combinations of opposing poly(A) modifying enzymes guide gene expression during early oogenesis	Nucleic Acids Research	Ineligible species
Jeppesen	2013	Which follicles make the most anti-Mullerian hormone in humans? Evidence for an abrupt decline in AMH production at the time of follicle selection	Mol Hum Repr	Ineligible study design
García-Láez	2011	Identification of gene expression of MYH11 and PF4V1 in cumulus cells (CC) after ovarian stimulation by microarrays technology: possible implications on oocyte maturation	Fertil Steril	Ineligible publication type
Sreenivasulu	2009	A role for cytochrome P450 17α-hydroxylase/c17-20 lyase during shift in steroidogenesis occurring in ovarian follicles prior to oocyte maturation	J. Steroid Biochem. Mol. Biol	Ineligible species
Lefevre	2016	A Mixture Reflecting Polybrominated Diphenyl Ether (PBDE) Profiles Detected in Human Follicular Fluid Significantly Affects Steroidogenesis and Induces Oxidative Stress in a Female Human Granulosa Cell Line	Endocrinology	Ineligible study design

Levi	2013	Morphological and molecular markers are correlated with maturation-competence of human oocytes	Hum Rep	Ineligible study design
Wei	2022	Disrupted expression of long non-coding RNAs in the human oocyte: the possible epigenetic culprits leading to recurrent oocyte maturation arrest	J Assist Reprod Genet	Ineligible population
Swartz	2021	Selective dephosphorylation by PP2A-B55 directs the meiosis I-meiosis II transition in oocytes	eLife	Ineligible species
Pelisch	2016	A SUMO-dependent Protein Network Regulates Chromosome Congression during oocyte meiosis	Molecular Cell	Ineligible species
Berger	2013	Glycolytic metabolites are critical modulators of oocyte maturation and viability	PLoS One	Ineligible species
Reich	2012	Transcriptome variance in single oocytes within, and between, genotypes	Mol Reprod Dev	Ineligible species
Twigt	2012	Proteomic analysis of the microenvironment of developing oocytes	Proteomics	Ineligible study design
Berendt	2008	Oocyte maturation in vitro: Proteome alterations analyzed by ultra-sensitive fluorochrome labeling and nano-esi-MS	Reprod.Fertil. Dev.	Ineligible species
Buyuk	2010	Reduced neurotrophin receptor TrkA gene expression in human granulosa cells: a novel marker of diminishing ovarian reserve?	Fertil Steril	Ineligible study design
Rowley	2020	Low Molecular Weight Hyaluronan Induces an Inflammatory Response in Ovarian Stromal Cells and Impairs Gamete Development in Vitro	IJMS	Ineligible species
Binder	2013	The absence of ER-beta results in altered gene expression in ovarian granulosa cells isolated from in vitro preovulatory follicles	Endocrinology	Ineligible species
Krieg	2018	Histone demethylase KDM4A and KDM4B expression in granulosa cells from women undergoing in vitro fertilization	J Assist Reprod Genet	Ineligible study design
Christin-Maitre	2010	Genome-wide association study and premature ovarian failure	Annales d'Endocrinologie	Ineligible study design
Demiray	2019	Differential gene expression analysis of human cumulus cells	Clin Exp Reprod Med	Ineligible study design
Song	2013	The Rhox homeobox gene cluster is selectively expressed in human oocytes and male germ cells	Hum Reprod	Ineligible study design
Devos	2023	Gene Expression analyses in human follicles	JoVE	Ineligible study design

Buigues	2021	Pathways and factors regulated by bone marrow-derived stem cells in human ovarian tissue	Fertil Steril	Ineligible study design
Duncan	2017	Egg Quality during the Pubertal Transition – Is Youth All It's cracked up to be?	Front. Endocrinol.	Ineligible publication type
Deng	2022	Whole Genome Transcriptomic Analysis of Ovary Granulosa Cells Revealed an Anti-apoptosis regulatory gene DLGAP5 in polycystic ovary syndrome	Front. Endocrinol.	Ineligible study design
Binelli	2010	Coordinated regulation of follicle development by germ and somatic cells	Reprod.Fertil. Dev.	Ineligible publication type
Desai	2017	Genetics of Reproductive Aging from Gonadal Dysgenesis through Menopause	Semin Reprod Med	Ineligible publication type
Wood	2013	Genomic Markers of Ovarian Reserve	Semin Reprod Med	Ineligible publication type
Zeng	2020	Critical roles of mRNA m6A modification and YTHDC2 expression for meiotic initiation and progression in female germ cells	Gene	Ineligible species
Huang	2018	CAV1 regulates primordial follicle formation via the Notch2 signalling pathway and is associated with premature ovarian insufficiency in humans	Hum Rep	Ineligible study design
Li	2021	Urinary vitamin D-binding protein as a marker of ovarian reserve	Reprod Biol Endocrinol	Ineligible study design
Laisk	2018	Large-scale meta-analysis highlights the hypothalamic pituitary-gonadal axis in the genetic regulation of menstrual cycle length	Human Molecular Genetics	Ineligible publication type
Ottolini	2016	Generation of meiomaps of genome-wide recombination and chromosome segregation in human oocytes	Nat Protoc	Ineligible publication type
Bildik	2020	Terminal differentiation of human granulosa cells as luteinization is reversed by activin-A through silencing of Jnk pathway	Cell Death Discov.	Ineligible study design
Man	2021	TOX3 promotes ovarian estrogen synthesis: an RNA-sequencing and network study	Front. Endocrinol.	Ineligible study design
Hanna	2018	MLL2 conveys transcription-independent H3K4 trimethylation in oocytes	Nat Struct Mol Biol	Ineligible species
Roos	2022	Single-cell RNA-seq analysis and cell-cluster deconvolution of the human preovulatory follicular fluid cells provide insights into the pathophysiology of ovarian hyporesponse	Front. Endocrinol.	Ineligible study design

Luong	2020	Genome-wide analysis reveals a switch in the translational program upon oocyte meiotic resumption	Nucleic Acids Research	Ineligible species
Papacleovoulou	2009	Regulation of 3-hydroxysteroid dehydrogenase type 1 and type 2 gene expression and function in the human ovarian surface epithelium by cytokines	Mol Human Rep	Ineligible study design
Chermula	2021	Cellular processes in human ovarian follicles are regulated by expression profile of new gene markers–Clinical Approach	JCM	Ineligible study design
Kwon	2010	mRNA expression pattern of insulin-like growth factor components of granulosa cells and cumulus cells in women with and without polycystic ovary syndrome according to oocyte maturity	Fertil Steril	Ineligible population
Cari	2021	Integrated stress response control of granulosa cell translation and proliferation during normal ovarian follicle development	Molecular Human Reproduction	Ineligible species
Puttabyatappa	2014	Ovarian Membrane-Type Matrix Metalloproteinases: Induction of MMP14 and MMP16 during the periovulatory period in the rat, macaque and human	Biology of Reproduction	Ineligible study design
Kristensen	2018	Hallmarks of Human Small Antral Follicle Development: Implications for Regulation of Ovarian Steroidogenesis and Selection of the Dominant Follicle	Front. Endocrinol.	Ineligible study design
Li	2021	CDC26 is a key factor in human oocyte aging	Hum. Rep.	Ineligible species
Younis	2012	Ovarian aging and implications for fertility female health	Minerva Endocrinol	Ineligible publication type
Rae	2009	Glucocorticoid receptor-mediated regulation of MMP9 gene expression in human ovarian surface epithelial cells	Fertil Steril	Ineligible study design
Zhu	2022	Melatonin antagonizes ovarian aging via YTHDF2-MAPK-NF-κB pathway	Genes and Diseases	Ineligible study design
Huo	2021	Effects of vitrification and cryostorage duration on single-cell RNA-seq profiling of vitrified-thawed human metaphase III oocytes	Front.Med.	Ineligible study design
Zhang	2022	Retinoic acid induced meiosis initiation in female germline stem cells by remodelling three-dimensional chromatin structure	Cell Proliferation	Ineligible species

St.John	2019	The transgenerational effects of oocyte mitochondrial supplementation	Sci Rep	Ineligible study design
Nagao	2019	Interaction of ovarian steroidogenesis and clock gene expression modulated by bone morphogenetic protein-7 in human granulosa cells	Endocr J	Ineligible study design
Ouandaogo	2012	Differences in transcriptomic profiles of human cumulus cells isolated from oocytes at GV, MI and MII stages after in vivo and in vitro oocyte maturation	Hum Rep	Ineligible study design
Zuccotti	2023	Single-follicle spatial proteomics of the whole ovary by MALDI mass spectrometry imaging: towards the identification of protein-markers of the follicle's quality	Hum Rep	Ineligible species; ineligible publication type
Muhammad	2023	Regulation of germline proteostasis by HSF1 and insulin IGF-1 signaling	Biochem. Soc.Trans.	Ineligible publication type
Li	2023	TGF-b1 regulates the lncRNA transcriptome of ovarian granulosa cells in a transcription activity-dependent manner	Cell Proliferation	Ineligible species
Zhou	2022	Dissecting the fate of Foxl2-expressing cells in fetal ovary using lineage tracing and single-cell transcriptomics	Cell Discov	Ineligible species
Shen	2022	TGF-β1 induces type I collagen deposition in granulosa cells via the AKT/GSK-3β signaling pathway-mediated MMP1 down-regulation	Reproductive Biology	Ineligible study design
Lu	2022	Metabolic dysregulation in patients with premature ovarian insufficiency revealed by integrated transcriptomic, methylomic and metabolomic analyses	Clinical & Translational Medicine	Ineligible population
Yu	2022	Dissection of transcriptome dysregulation and immune characterization in women with germline BRCA1 mutation at single-cell resolution	BMC Med	Ineligible population; ineligible cell population (fallopian tube mucosae)
Lin	2022	NAT10 Maintains *OGA* mRNA Stability Through ac4C Modification in Regulating Oocyte Maturation	Front. Endocrinol	Ineligible species
Moura	2021	Folliculogenesis-related genes are differently expressed in secondary and tertiary ovarian follicles	Zygote	Ineligible species

Venturas	2021	Metabolic imaging of human cumulus cells reveals associations among metabolic profiles of cumulus cells, patient clinical factors, and oocyte maturity	Fertil Steril	Ineligible study design
Luo	2021	Bone morphogenetic protein 2 inhibits growth differentiation factor 8-induced cell signaling via upregulation of gremlin2 expression in human granulosa-lutein cells	Reprod Biol Endocrinol	Ineligible study design; ineligible cell population
Li	2021	TGF-β1 inhibits microvascular-like formation by decreasing VCAM1 and ICAM1 via the upregulation of SNAIL in human granulosa cells	Mol Cell Endocrinol	Ineligible cell population
Parks	2022	The interplay between nuclear meiotic errors and mitochondrial DNA content drive oocyte aneuploidy	Fertil Steril	Ineligible publication type
Haywood	2021	The dynamic ageing transcriptome architecture of the ovary, follicle, and oocyte	Fertil Steril	Ineligible publication type
Emidio	2021	Possible role of NAD+ metabolism in oocyte aging: insights from gene expression profiles in reproductive aged oocytes	Hum Rep	Ineligible species
McCallie	2021	Forecasting early onset diminished ovarian reserve for young reproductive age women	J Assist Reprod Genet	Ineligible cell population
Lim	2021	Dysregulation of bisphosphoglycerate mutase during in vitro maturation of oocytes	J Assist Reprod Genet	Ineligible study design
Suyama	2021	Roles of NR5A1 and NR5A2 in the regulation of steroidogenesis by Clock gene and bone morphogenetic proteins by human granulosa cells	Endocr J	Ineligible study design
Chen	2021	C28 induced autophagy of female germline stem cells in vitro with changes of H3K27 acetylation and transcriptomics	Gene	Ineligible species
Campi	2021	A Topological Data Analysis Mapper of the ovarian folliculogenesis based on MALDI Mass Spectometry Imaging Proteomics – Artificial Intelligence in Medicine	Lecture Notes in Computer Science	Ineligible publication type
Paradiso	2021	Sphingosine-1 phosphate induces cAMP/PKA-independent phosphorylation of the cAMP response element-binding protein CREB in granulosa cells	Mol Cell Endocrinol	Ineligible study design
Park	2020	TOPK inhibition accelerates oxidative stress-induced granulosa cell apoptosis via the p53/SIRT1 axis	Int J Mol Med	Ineligible study design

Rodgers	2020	Genetic relationships between early menopause and the behaviour of theca interna during follicular atresia	Hum Rep	Ineligible publication type
He	2020	Using bioinformatics and metabolomics to identify altered granulosa cells in patients with diminished ovarian reserve	PeerJ	Ineligible population
Battaglia	2020	Ovarian aging increases small extracellular vesicle CD81+ release in human follicular fluid and influences miRNA profiles	Aging	Ineligible study design
Lan	2020	The mitochondrial {DNA} copy number of cumulus granulosa cells may be related to the maturity of oocyte cytoplasm	Hum Rep	Ineligible study design
Zhang	2020	CDCA8 regulates meiotic spindle assembly and chromosome segregation during human oocyte meiosis	Gene	Ineligible study design
Lara-Molina	2020	Cumulus cells have longer telomeres than leukocytes in reproductive-age women	Fertil Steril	Ineligible study design
Takahashi	2019	Accumulation of advanced glycation end products in follicles is associated with poor oocyte developmental competence	Mol Hum Reprod	Ineligible study design
Harpelunde Poulsen	2019	Dysregulation of FGFR signaling by a selective inhibitor reduces germ cell survival in human fetal gonads of both sexes and alters the somatic niche in fetal testes	Hum Rep	Ineligible study design
Roeles	2019	Actin-microtubule interplay coordinates spindle assembly in human oocytes	Nat Commun	Ineligible study design
Kim	2019	Gas6 is a reciprocal regulator of mitophagy during mammalian oocyte maturation	Sci Rep	Ineligible species
Casalechi	2019	C-type natriuretic peptide signaling in human follicular environment and its relation with oocyte maturation	Molecular and Cellular Endocrinology	Ineligible study design
Miao	2019	BRCA2 deficiency is a potential driver for human primary ovarian insufficiency	Cell Death Dis	Ineligible population; ineligible species
Huang	2019	Developmental features of DNA methylation in CpG islands of human gametes and preimplantation embryos	Exp Ther Med	Ineligible study design
Poulsen	2019	Human granulosa cells function as innate immune cells executing an inflammatory reaction during ovulation: a microarray analysis	Molecular and Cellular Endocrinology	Ineligible study design

Grosbois	2018	Dynamics of PI3K and Hippo signaling pathways during in vitro human follicle activation	Hum Rep	Ineligible study design
Zhang	2018	BMP6-downregulated GDNF expression through SMAD1/5 and ERK1/2 signaling pathways in human granulosa-lutein cells	Endocrinology	Ineligible study design
Cui	2018	GDNF-induced downregulation of miR-145-5p enhances human oocyte maturation and cumulus cell viability	J Clin Endocrinol Metab	Ineligible study design
Eslami	2018	Epigenetic aberration of FMR1 gene in infertile women with diminished ovarian reserve	Cell J	Ineligible population
Canosa	2017	Zona pellucida gene mRNA expression in human oocytes is related to oocyte maturity, zona inner layer retardance and fertilization competence	Mol.Hum. Reprod.	Ineligible study design
Yanez	2016	Human oocyte developmental potential is predicted by mechanical properties within hours after fertilization	Nat Commun	Ineligible study design
Agirregoitia	2015	Dynamics of expression and localization of the cannabinoid system in granulosa cells during oocyte nuclear maturation	Fertil Steril	Ineligible study design
Baumgarten	2015	FSH regulates IGF-2 expression in human granulosa cells in an AKT-Dependent Manner	J Clin Endocrinol Metab	Ineligible study design
Dumesic	2015	Oocyte environment: follicular fluid and cumulus cells are critical for oocyte health	Fertil Steril	Ineligible publication type
Gianaroli	2015	Mitochondrial DNA analysis and numerical chromosome condition in human oocytes and polar bodies	Mol Hum Reprod	Ineligible study design
Garcia-Ortega	2014	Expression of neurokinin B/NK3 receptor and kisspeptin KISS1 receptor in human granulosa cells	Hum Rep	Ineligible study design
Barzilay	2014	Differential expression of poliovirus receptor, regulator of G-protein signaling 11 and erythrocyte protein band 4.1-like 3 in human granulosa cells during follicular growth and maturation	Gynecological Endocrinology	Ineligible study design
Diez-Fraile	2014	Age-associated differential microRNA levels in human follicular fluid reveal pathways potentially determining fertility and success of in vitro fertilization	Hum Fert	Ineligible study design

Otsuki	2014	Aggregated chromosomes transfer in human oocytes	RBMO	Ineligible study design
Khouiery	2008	Dynamic CpG methylation of the KCNQ1OT1 gene during maturation of human oocytes	J Med Genet	Ineligible study design
Sun	2010	Expression of GDF9, BMP15 and their receptors in mammalian ovary follicles	J Mol histol	Ineligible study design
Jeppesen	2012	Concentration of activin A and follistanin in follicular fluid from small antral follicles associated to gene expression of the corresponding granulosa cells	Mol Cel Endocrinol	Ineligible study design
Sirotkin	2009	Protein kinases controlling PCNA and p53 expression in human ovarian cells	Funct Integr Genomics	Ineligible study design
Matsuda	2008	cFIP regulates death receptor-mediated apoptosis in an ovarian granulosa cell line by inhibiting procaspase-8 cleavage	J Reprod Dev	Ineligible study design
Abir	2010	Vascular endothelial growth factor A and its two receptors in human preantral follicles from fetuses, girls and women	Fertil Steril	Ineligible study design
Ito	2011	GSTT1 is upregulated by oxidative stress through p38-MK2 signaling pathway in human granulosa cells: possible association with mitochondrial activity	Aging (Albany NY)	Ineligible study design
Garcia-Cruz	2010	Dynamics of cohesin proteins REC8, STAG3, SMC1 and SMC3 are consistent with a role in sister chromatid cohesion during meiosis in human oocytes	Hum Rep	Ineligible study design
Lobb	2009	Expression and actions of transforming growth factors during human follicular development	Fertil Steril	Ineligible study design
Cheng	2009	Meiotic recombination in human oocytes	PLoS Genet	Ineligible study design
Chiang	2010	Evidence that Weakened Centromere Cohesion Is a Leading Cause of Age-Related Aneuploidy in Oocytes	Current Biol	Ineligible species
Myers	2008	Activin A reduces luteinisation of human luteinised granulosa cells and has opposing effects to human chorionic gonadotropin in vitro	Journal of Endocrinology	Ineligible study design
Munne	2012	Intra-age, intercenter, and intercycle differences in chromosome abnormalities in oocytes	Fertil and Steril	Ineligible method (no sequencing and MS involved)

Daina	2014	Non-meiotic chromosome instability in human immature oocytes	Eur J Hum Genet	Ineligible method (no sequencing and MS involved)
Tesfaye	2009	Gene expression profile of cumulus cells derived from cumulus - oocyte complexes matured either in vivo or in vitro	Reprod.Fertil. Dev	Ineligible study design
Gasca	2008	Total fertilization failure and molecular abnormalities in metaphase II oocytes	RBMO	Ineligible study design
Li	2008	Revisiting oocyte-somatic cell interactions: in search of novel intrafollicular predictors and regulators of oocyte developmental competence	Mol Human Rep	Ineligible publication type
Assou	2009	A gene expression signature shared by human mature oocytes and embryonic stem cells	BMC Genomics	Ineligible study design
Tuck	2015	Expression and localisation of c-kit and KITL in the adult human ovary	J Ovarian Res	Ineligible study design
McGlacken-Byrne	2022	ZSWIM7 is Associated With Human Female Meiosis and Familial Primary Ovarian Insufficiency	J Clin Endocrinol Metab	Ineligible study design
He	2013	A developmental stage-specific switch from DAZL to BOLL occurs during fetal oogenesis in humans, but not mice	PLoS One	Ineligible study design
Ernst	2018	Transcripts encoding free radical scavengers in human granulosa cells from primordial and primary ovarian follicles	J Assist Reprod Genet	Ineligible study design
Bayne	2015	GDF9 is transiently expressed in oocytes before follicle formation in the human fetal ovary and is regulated by a novel NOBOX transcript	PLoS One	Ineligible study design
Li	2021	Abnormal PIWI-interacting RNA profile and its association with the deformed extracellular matrix of oocytes from recurrent oocyte maturation arrest patients	Fertil Steril	Ineligible population
Yang	2021	A homozygous variant in TBPL2 was identified in women with oocyte maturation defects and infertility	Hum Reprod	Ineligible population
Albamonte	2013	The infant and pubertal human ovary: Balbiani's body-associated VASA expression, immunohistochemical detection of apoptosis-related BCL2 and BAX proteins, and DNA fragmentation	Hum Reprod	Ineligible study design

Törmälä	2008	Zona pellucida components are present in human fetal ovary before follicle formation	Mol Cell Endocrinol	Ineligible study design
Bøtkjær	2019	Transcription profile of the insulin-like growth factor signaling pathway during human ovarian follicular development	J Assist Reprod Genet	Ineligible study design
Albamonte	2020	PTEN and FOXO3 expression in the prenatal and postnatal human ovary	J Assist Reprod Genet	Ineligible study design
Duffin	2009	The forkhead transcription factor FOXL2 is expressed in somatic cells of the human ovary prior to follicle formation	Mol Hum Reprod	Ineligible study design
Kristensen	2014	Expression of TGF-beta superfamily growth factors, their receptors, the associated SMADs and antagonists in five isolated size-matched populations of pre-antral follicles from normal human ovaries	Mol Hum Reprod	Ineligible study design
Virant-Klun	2018	Human oocyte maturation in vitro is improved by co-culture with cumulus cells from mature oocytes	Reprod Biomed Online	Ineligible study design
Yang	2022	Changes in the mitochondria-related nuclear gene expression profile during human oocyte maturation by the IVM technique	Cells	Ineligible study design
Fragouli	2012	Alteration of gene expression in human cumulus cells as a potential indicator of oocyte aneuploidy	Hum Reprod	Ineligible study design
Grive	2016	TAF4b regulates oocyte-specific genes essential for meiosis	PLoS Genet	Ineligible study design
Huntriss	2017	Isolation and expression of the human gametocyte-specific factor 1 gene (GTSF1) in fetal ovary, oocytes, and preimplantation embryos	J Assist Reprod Genet	Ineligible study design
Guzeloglu-Kayisli	2014	Human embryonic poly(A)-binding protein (EPAB) alternative splicing is differentially regulated in human oocytes and embryos	Mol Hum Reprod	Ineligible study design
Bayne	2016	BMP signalling in human fetal ovary somatic cells is modulated in a gene-specific fashion by GREM1 and GREM2	Mol Hum Reprod	Ineligible study design
Regan	2016	Dysregulation of granulosal bone morphogenetic protein receptor 1B density is associated with reduced ovarian reserve and the age-related decline in human fertility	Mol Cell Endocrinol	Ineligible study design
Haverfield	2017	Tri-directional anaphases as a novel chromosome segregation defect in human oocytes	Hum Reprod	Ineligible study design

Oron	2012	Fibroblast growth factor 10 in human ovaries	RBMO	Ineligible study design
Guglielmino	2011	TAp73 is downregulated in oocytes from women of advanced reproductive age	Cell Cycle	Ineligible study design
O'Shea	2017	ATRX is a novel progesterone-regulated protein and biomarker of low developmental potential in mammalian oocytes	Reproduction	Ineligible species
Rosario	2017	RNA immunoprecipitation identifies novel targets of DAZL in human foetal ovary	Mol Hum Reprod	Ineligible study design
Adam	2012	Decorin is a part of the ovarian extracellular matrix in primates and may act as a signaling molecule	Hum Reprod	Ineligible study design
Ribeiro	2017	Age-related expression of TGF beta family receptors in human cumulus oophorus cells	J Assist Reprod Genet	Ineligible study design
Cakmak	2016	Dynamic secretion during meiotic reentry integrates the function of the oocyte and cumulus cells	PNAS	Ineligible study design
Jiajie	2017	Conserved miR-10 family represses proliferation and induces apoptosis in ovarian granulosa cells	Sci Rep	Ineligible study design
Jarkovska	2010	Proteome mining of human follicular fluid reveals a crucial role of complement cascade and key biological pathways in women undergoing in vitro fertilization	J Proteome Res	Ineligible study design
Childs	2009	Activin A selectively represses expression of the membrane-bound isoform of Kit ligand in human fetal ovary	Fertil Steril	Ineligible study design
Liu	2016	ECAT1 is essential for human oocyte maturation and pre-implantation development of the resulting embryos	Sci Rep	Ineligible study design
Eddie	2012	Developmentally regulated IL6-type cytokines signal to germ cells in the human fetal ovary	Mol Hum Reprod	Ineligible study design
Ottolini	2015	Genome-wide maps of recombination and chromosome segregation in human oocytes and embryos show selection for maternal recombination rates	Nat Genet	Ineligible study design
Luddi	2018	Matrix metalloproteinases and their inhibitors in human cumulus and granulosa cells as biomarkers for oocyte quality estimation	Fertil Steril	Ineligible study design
Rehnitz	2021	FMR1 expression in human granulosa cells and variable ovarian response: control by epigenetic mechanisms	Mol Hum Reprod	Ineligible study design

Koks	2010	The differential transcriptome and ontology profiles of floating and cumulus granulosa cells in stimulated human antral follicles	Mol Hum Reprod	Ineligible cell population
Coutts	2008	Activin signals via SMAD2/3 between germ and somatic cells in the human fetal ovary and regulates kit ligand expression	Dev Biol	Ineligible study design
Tsutsumi	2014	Age-related decrease of meiotic cohesins in human oocytes	PLoS One	Ineligible method (no sequencing and MS involved)
Childs	2010	Differential expression and regulation by activin of the neurotrophins BDNF and NT4 during human and mouse ovarian development	Dev Dyn	Ineligible study design
Tomaselli	2011	Human RSPO1/R-spondin1 is expressed during early ovary development and augments beta-catenin signaling	PLoS One	Ineligible study design
Uysal	2021	Decreased expression of TERT and telomeric proteins as human ovaries age may cause telomere shortening	J Assist Reprod Genet	Ineligible study design
Ota	2008	HOX cofactors expression and regulation in the human ovary	Reprod Biol Endocrinol	Ineligible study design
Rooda	2020	Target prediction and validation of microRNAs expressed from FSHR and aromatase genes in human ovarian granulosa cells	Sci Rep	Ineligible study design
Chang	2016	Activin A-induced increase in LOX activity in human granulosa-lutein cells is mediated by CTGF	Reproduction	Ineligible study design
Fainaru	2011	Maturation of human ovarian follicles is accompanied by a decrease in the CD56+CD16+ natural killer cell population	Fertil Steril	Ineligible publication type
Childs	2012	LIN28 is selectively expressed by primordial and pre-meiotic germ cells in the human fetal ovary	Stem Cells Dev	Ineligible study design
Valerio	2018	SA1/SA2 cohesion proteins and SIRT1-NAD+ deacetylase modulate telomere homeostasis in cumulus cells and are eligible biomarkers of ovarian aging	Hum Reprod	Ineligible study design
Park	2021	Role of the SIRT1/p53 regulatory axis in oxidative stress-mediated granulosa cell apoptosis	Mol Med Rep	Ineligible method (no sequencing and MS involved)
Garcia-Cruz	2010	Dynamics of cohesin proteins REC8, STAG3, SMC1 beta and SMC3 are consistent with a role	Hum Reprod	Ineligible method (no sequencing and MS involved)

		in sister chromatid cohesion during meiosis in human oocytes		
Jin	2017	EGR2 is a gonadotropin-induced survival factor that controls the expression of IER3 in ovarian granulosa cells	Biochem Biophys Res Commun	Ineligible study design
Chang	2016	Growth differentiation factor 8 suppresses cell proliferation by up-regulating CTGF expression in human granulosa cells	Mol Cell Endocrinol	Ineligible study design
Santonocito	2014	Molecular characterization of exosomes and their microRNA cargo in human follicular fluid: bioinformatic analysis reveals that exosomal microRNAs control pathways involved in follicular maturation	Fertil Steril	Ineligible study design
So	2022	Mechanism of spindle pole organization and instability in human oocytes	Science	Ineligible method (no sequencing and MS involved)
Wang	2017	Inefficient Crossover Maturation Underlies Elevated Aneuploidy in Human Female Meiosis	Cell	Ineligible method (no sequencing and MS involved)
Zielinska	2015	Sister kinetochore splitting and precocious disintegration of bivalents could explain the maternal age effect	eLife	Ineligible method (no sequencing and MS involved)
Holubcova	2015	Human oocytes. Error-prone chromosome-mediated spindle assembly favors chromosome segregation defects in human oocytes	Science	Ineligible method (no sequencing and MS involved)
Hirano	2017	The emerging role of FOXL2 in regulating the transcriptiponal activation function of estrogen receptor beta: An insight into ovarian folliculogenesis	Reprod.Sci.	Ineligible study design
Wells	2008	Gene expression profiling of human oocytes at different maturational stages and after in vitro maturation	AJOG	Ineligible publication type
Fowler	2011	Development of Steroid Signaling Pathways During Primordial Follicle Formation in the Human Fetal Ovary	J.Clin. Endocrinol. Metab.	Ineligible study design
Jin	2016	FOXL2 is an essential activator of SF1-induced transcriptional regulation of Anti-Mullerian Hormone in Human Granulosa Cells	PLoS One	Ineligible study design
Patel	2015	Unique geometry of sister kinetochores in human oocytes during meiosis I	Biol Open	Ineligible method (no sequencing and MS involved)

		may explain maternal age-associated increases in chromosomal abnormalities		
Sacchi	2017	The Anti-Mullerian hormone (AMH) includes forkhead box L2 (FOXL2) expression in primary culture of human granulosa cells in vitro	J Assist Reprod Genet	Ineligible study design
Fan	2024	TP63 truncating mutation causes increased cell apoptosis and premature ovarian insufficiency by enhanced transcriptional activation of CLCA2	J Ovarian Res	Ineligible population
Childs	2011	Retinoic Acid Signaling and the Control of Meiotic Entry in the Human Fetal Gonad	PLoS One	Ineligible study design
Gokyer	2024	The oocyte microenvironment is altered in adolescents compared to oocyte donors	bioaRxiV	Ineligible publication type
Rezaei-Tazangi	2024	Effects of kisspeptin on the maturation of human ovarian primordial follicles in vitro	Zygote	Ineligible study design
Akiyama	2014	Bone morphogenetic protein 7 increased vascular endothelial growth factor (VEGF)-a expression in human granulosa cells and VEGF receptor expression in endothelial cells	Reprod Sci	Ineligible study design
Chang	2016	Connective tissue growth factor mediates growth differentiation factor 8-induced increase of lysyl oxidase activity in human granulosa-lutein cells	Mol Cell Endocrinol	Ineligible study design
Qian	2016	Implication of differential peroxiredoxin 4 expression with age in ovaries of mouse and human for ovarian aging	Curr Mol Med	Ineligible study design
Nie	2015	miR-23a and miR-27a promote human granulosa cell apoptosis by targeting SMAD5	Biol Reprod	Ineligible study design
Barragana	2023	The expression levels of NOS2, HMOX1, and VEGFC in cumulus cells are markers of oocyte maturation and fertilization rate	Mol Reprod Dev	Ineligible study design
Reich	2011	The transcriptome of a human polar body accurately reflects its sibling oocyte	J Biol Chem	Ineligible study design
Mizuta	2022	Ex vivo reconstruction of fetal oocyte development in humans and cynomolgus monkeys	EMBO J	Ineligible study design
Hong	2018	miR-106a increases granulosa cell viability and is downregulated in women with diminished ovarian reserve	J Clin Endocrinol Metab	Ineligible population
Nikalayevich	2024	Abberant cortex contractions impact mammalian oocyte quality	Dev Cell	Ineligible method

Lagir	2017	Loss of Centromere Cohesion in Aneuploid Human Oocytes Correlates with Decreased Kinetochore Localization of the Sac Proteins Bub1 and Bubr1	Sci Rep	Ineligible method (no sequencing and MS involved)
Skiadas	2012	Ovarian reserve status in young women is associated with altered gene expression in membrana granulosa cells	Mol Hum Reprod	Ineligible population
Lin	2020	Combining Bioinformatics and Experiments to Identify CREB1 as a Key Regulator in Senescent Granulosa Cells	Diagnostics	Ineligible study design
Wu	2019	Bone morphogenetic protein 6 affects cell-cell communication by altering the expression of connexin43 in human granulosa-lutein cells	Mol Cell Endocrinol	Ineligible study design
Kawamura	2024	BMAL1 positively correlated with genes regulating steroidogenesis in human lutenized granulosa cells	Reproduction	Ineligible study design
Lv	2024	Molecular mechanisms of cadmium-induced cytotoxicity in human ovarian granulosa cells identified using integrated omics	Ecotoxicol Environ Saf	Ineligible study design
Bothun	2019	Dynamics of WNT signaling components in the human ovary from development to adulthood	Histochem Cell Biol	Ineligible study design
Mihalas	2024	Age-dependent loss of cohesion protection in human oocytes	Curr Biol	Ineligible method
Gook	2007	Detection of zona pellucida proteins during human folliculogenesis	Hum Repr	Ineligible study design
Dobrynin	2020	Human pericentromeric tandemly repeated DNA is transcribed at the end of oocyte maturation and is associated with membraneless mitochondria-associated structures	Sci Rep	Ineligible study design
Ernst	2020	Distinct expression patterns of TLR transcripts in human oocytes and granulosa cells from primordial and primary follicles	Journal of Reproductive Immunology	Ineligible study design
Alberico	2022	Workflow Optimization for Identification of Female Germline or Oogonial Stem Cells in Human Ovarian Cortex Using Single-Cell RNA Sequence Analysis	Stem Cells	Ineligible study design (no new data)
Chitiashvili	2022	FGFR3 is expressed by human primordial germ cells and is repressed after meiotic initiation to form primordial oocytes	Stem Cell Rep	Ineligible study design (no new data)

Ducreux	2023	Overview of Gene Expression Dynamics during Human Oogenesis/Folliculogenesis	Int J Mol Sci	Ineligible publication type
Caponnetto	2024	A Circular RNA Derived from the Pumilio 1 Gene Could Regulate PTEN in Human Cumulus Cells	Genes	Ineligible method (no sequencing and MS involved)
Zhang	2014	Identification of differentially expressed proteins in the ovaries of menopausal women	Arch Gynecol Obstet	Ineligible method (no sequencing and MS involved)
Zielinska	2019	Meiotic Kinetochores Fragment into Multiple Lobes upon Cohesin Loss in Aging Eggs	Curr Biol	Ineligible method (no sequencing and MS involved)
van den Berg	2011	Defective deacetylation of histone 4 K12 in human oocytes is associated with advanced maternal age and chromosome misalignment	Hum Reprod	Ineligible method (no sequencing and MS involved)
Jin	2022	The regulatory landscapes of human ovarian ageing	bioRxiv	Ineligible publication type
Xu	2021	Integrated analyses of the single-cell ATAC-seq and RNA-seq reveal the epigenetic landscape of human ovarian aging	bioRxiv	Ineligible publication type
Roovers	2015	Piwi proteins and piRNAs in mammalian oocytes and early embryos	Cell Rep	Ineligible study design
Ernst	2018	Transcriptome analysis of long non-coding RNAs and genes encoding paraspeckle proteins during human ovarian follicle development	Front. Cell Dev. Biol.,	Ineligible study design
Steffensen	2018	Transcripts encoding the androgen receptor and IGF-related molecules are differentially expressed in human granulosa cells from Primordial and Primary Follicles	Front. Cell Dev. Biol.,	Ineligible study design
Wang	2022	A Single-Cell Omics Network Model of Cell Crosstalk during the Formation of Primordial Follicles	Cells	Ineligible study design

Grøndahl	2013	The dormant and the fully competent oocyte: comparing the transcriptome of human oocytes from primordial follicles and in metaphase II	Mol Hum Reprod.	Ineligible study design
Wang	2021	Cell-specific network analysis of human folliculogenesis reveals network rewiring in antral stage oocytes	J Cell Mol Med	Ineligible study design
Brazert	2019	Transcriptomic profile of cell cycle progression genes in human ovarian granulosa cells	J Biol Regul Homeost Agents	Ineligible study design
Pasquariello	2018	Alterations in oocyte mitochondrial number and function are related to spindle defects and occur with maternal aging in mice and humans	Biol Reprod.	Ineligible method (no sequencing and MS involved)
Lu	2024	Aging hallmarks of the primate ovary revealed by spatiotemporal transcriptomics	Protein Cell.	Ineligible species
Wang	2024	Association of serum uric acid with women's ovarian reserve: observational study and Mendelian randomization analyses	Fertil. Steril.	Ineligible study design
Zhang	2024	HDAC6-dependent deacetylation of NGF dictates its ubiquitination and maintains primordial follicle dormancy	Theranostics	Ineligible study design
Giaccari	2024	A maternal effect *Padi6* variant causes nuclear and cytoplasmic abnormalities in oocytes, as well as failure of epigenetic reprogramming and zygotic genome activation in embryos	Genes Dev.	Ineligible species
Wang	2025	Core fucosylation regulates the ovarian response via FSH receptor during follicular development	J Adv Res.	Ineligible method (no sequencing and MS involved)
Li	2024	ARHGAP26 deficiency drives the oocyte aneuploidy and early embryonic development failure	Cell Death Differ.	Ineligible species

Lu	2024	Integrative bioinformatics analysis for identifying the mitochondrial-related gene signature associated with immune infiltration in premature ovarian insufficiency	BMC Med.	Ineligible study population
Nikalayevich	2024	Aberrant cortex contractions impact mammalian oocyte quality	Dev Cell	Ineligible method (no sequencing and MS involved)
Yahya	2024	Differential regulation of STARD1, STARD4 and STARD6 in the human ovary	J Endocrinol.	Ineligible study design
Liu	2024	Proteomics profiling reveals lipid metabolism abnormalities during oogenesis in unexplained pregnancy loss	Front Immunol	Ineligible study population
Bellou	2024	Chromosome architecture and low cohesion bias acrocentric chromosomes towards aneuploidy during mammalian meiosis	Nat Comm	Ineligible method (no sequencing and MS involved)
Cao	2024	METTL3 deficiency leads to ovarian insufficiency due to IL-1beta overexpression in theca cells	Free Radic Biol Med	Ineligible study population
Hu	2025	CKAP5 deficiency induces premature ovarian insufficiency	EBioMedicine	Ineligible study population
Zhang	2025	Revisiting the rete ovarii	Elife	Ineligible publication type
Du	2024	Identifying therapeutic targets for primary ovarian insufficiency through integrated genomic analyses	J Ovarian Res	Ineligible study population
Mihalas	2024	Age-dependent loss of cohesion protection in human oocytes	Curr Biology	Ineligible method (no sequencing and MS involved)
Zhu	2025	Ovarian remodeling and aging-related chronic inflammation and fibrosis in the mammalian ovary	J Ovarian Res	Ineligible species
Zhou	2025	A Microtubule-associated protein functions in preventing oocytes from evading the spindle	Adv Sci	Ineligible method

		assembly checkpoint		(no sequencing and MS involved)
Xie	2024	Effects of multisuperovulation on the transcription and genomic methylation of oocytes and offspring	Clin Epigenetics	Ineligible study design
Biswas	2024	Maternal genetic variants in kinesin motor domains prematurely increase egg aneuploidy	PNAS	Ineligible tissue
Hu	2025	Ovarian aging-associated downregulation of GPX4 expression regulates ovarian follicular development by affecting granulosa cell functions and oocyte quality	FASEB J	Ineligible method (no sequencing and MS involved)
Sperduti	2025	Gene expression pattern predictive of human ovarian follicle development and maturation	Reproduction	Ineligible study design
N/A	2025	Multiomics atlas reveals molecular and genetic drivers of human ovarian aging	Nat Aging	Ineligible publication type
Daugelaite	2025	Granulosa cell transcription is similarly impacted by superovulation and aging and predicts early embryonic trajectories	Nat Comm	Ineligible study design
Zhou	2025	Comparatively profiling the transcriptome of human, porcine and mouse oocytes undergoing meiotic maturation	BMC Genomics	Ineligible study design
Rong	2025	ZAR1/2-Regulated Epigenetic Modifications are Essential for Age-Associated Oocyte Quality Maintenance and Zygotic Activation	Adv Sci	Ineligible species
Pietroforte	2024	Age-associated increased stiffness of the ovarian microenvironment impairs follicle development and oocyte quality and rapidly alters follicle gene expression	bioRxiv	Ineligible publication type
Kawamura	2024	BMAL1 positively correlates with genes regulating steroidogenesis in human luteinized granulosa cells	Reproduction	Ineligible study design

Zhang	2024	Single-cell RNA sequencing of human oocytes reveals a differential transcriptomic profile associated with agar-like zona pellucida	J Ovarian Res	Ineligible study design
Danti	2025	*FOXL2* drives the differentiation of supporting gonadal cells in early ovarian development	Reprod Biol Endocrinol	Ineligible cell population
He	2025	P300 maintains primordial follicle activation by repressing *VEGFA* transcription	Am J Physiol Cell Physiol	Ineligible species
Qin	2025	Decoding the molecular landscape of prepubertal oocyte maturation: GTPB4 as a Key Driver of in vitro developmental competence	Cell Prolif	Ineligible species
Sananmuang	2024	Differential transcript usage across mammalian oocytes at the germinal vesicle and metaphase II stage	Theriogenology	Ineligible study design
Shan	2024	miR-184, a downregulated ovary-elevated miRNA transcriptionally activated by SREBF2, exerts anti-apoptotic properties in ovarian granulosa cells through inducing *SMAD3* expression	Cell Death Dis	Ineligible species
Cao & Grow	2024	TCF3, TCF12 and distinct enhancers regulate oocyte transcription	Nat Cell Bio	Ineligible publication type
Nguyen	2025	AMH protects the ovary from doxorubicin by regulating cell fate and the response to the DNA damage	PNAS	Ineligible species
Anbarci	2025	Rediscovering the *rete ovarii*, a secreting auxiliary structure to the ovary	eLife	Ineligible species
Jensen	2025	Distinct luteinization profiles of cultured human granulosa cells from small antral and preovulatory follicles	J Clin Endocrinol Metab	Ineligible method
Cooper	2024	Oocyte mitochondria link maternal environment to offspring phenotype	Res Sq	Ineligible publication type, Ineligible species

Li	2025	Intermediate filament protein BFSP2 controls spindle formation via HSC70-mediated stabilization of CLTC during oocyte meiosis	Adv Sci	Ineligible species
Soldatkina	2025	Multimodal data analysis reveals asynchronous aging dynamics across female reproductive organs	bioRxiv	Ineligible publication type
Panagitou	2025	Single-cell transcriptomic profiling reveals mehp-induced disruption of core cellular processes across all cell types in human ovarian cortex	SSRN	Ineligible publication type
Surmacki	2024	The role of cytochrome c in mitochondrial metabolism of human oocytes	bioRxiv	Ineligible publication type
Ozturk	2025	The dynamics of DNA methylation, histone methylation and acetylation during oocyte aging in mammalian species and possible interventions to regulate them	JARG	Ineligible publication type
Hao	2025	Blood-detected mitochondrial biomarker NSUN4: a potential indicator of ovarian aging	Exp Gerontol	Ineligible publication type
Rix & Mogessie	2025	In preprints: rethinking the foundations of the ovarian reserve	Development	Ineligible publication type
Cai	2025	SOX3 facilitates granulosa cell proliferation and suppresses cell apoptosis through modulating PI3K/AKT pathway by targeting SPP1	Cell Mol Life Sci	Ineligible method (no sequencing and MS involved)
Wang	2025	Single-cell metabolomics reveals that bisphosphoglycerate mutase influences oocyte maturation through glucose metabolism	Mol Hum Rep	Ineligible species
Wang	2025	Revitalising Aging Oocytes: Echinacoside restores mitochondrial Function and cellular homeostasis through targeting GJA1/SIRT1 pathway	Cell Prolif	Ineligible species, ineligible study design
Machlin	2025	Single-cell analysis comparing early-stage oocytes from fresh and slow-frozen/thawed	Hum Rep	Ineligible study design

		human ovarian cortex reveals impact of cryopreservation on the oocyte transcriptome		
Gao	2024	Age-associated accumulation of RAB9 disrupts oocyte meiosis	Aging Cell	Ineligible species, ineligible method (no sequencing and MS involved)
Baddela	2025	Comparative analysis of PI3K-AKT and MEK-ERK1/2 signaling-driven molecular changes in granulosa cells	Reproduction	Ineligible species
Wu	2024	Activation of interleukin 33-NFkB axis in granulosa cells during atresia and its role in disposal of atretic follicles	Biol Reprod	Ineligible species
Liu	2024	Zinc deficiency deteriorates ovarian follicle development and function by inhibiting mitochondrial function	J Ovarian Res	Ineligible species, ineligible method (no sequencing and MS involved)
Nagyova	2024	Unique hyaluronan structure of expanded oocyte-cumulus extracellular matrix in ovarian follicles	Endocr Regul	Ineligible publication type
Zhang	2024	The evolution of ovarian somatic cells characterized by transcriptome and chromatin accessibility across rodents, monkeys, and humans	Life Medicine	Ineligible study design
Ren	2024	$CRL4^{DCAF13}$ E3 ubiquitin ligase targets MeCP2 for degradation to prevent DNA hypermethylation and ensure normal transcription in growing oocytes	Cell Mol Life Sci	Ineligible species
Rautela	2024	A non-canonical role of somatic Cyclin D/CYD-1 in oogenesis and in maintenance of reproductive fidelity, dependent on the FOXO/DAF-16 activation state	PLoS Genet	Ineligible species
Ozturk	2024	The close relationship between oocyte aging and telomere shortening, and possible interventions for telomere protection	Mech Ageing Dev.	Ineligible publication type
Anderson	2025	Oocyte development: it’s all about quality	RBMO	Ineligible publication type

Spector	2024	Anti-Mullerian hormone signaling in the ovary involves stromal fibroblasts: a study in humans and mice provides novel insights into the role of ovarian stroma	Hum Reprod	Ineligible method (no sequencing and MS involved)
Peshkin	2025	Decoding protein phosphorylation during oocyte meiotic divisions using phosphoproteomics	eLife	Ineligible species
Peresico	2025	Transcriptomic profile of early antral follicles: predictive somatic gene markers of oocyte maturation outcome	Cells	Ineligible species
Zhu	2024	LSD1 promotes the FSH responsive follicle formation by regulating autophagy and repressing *Wt1* in the granulosa cells	Sci Bull (Beijing)	Ineligible species
Sidell	2024	Retinoic acid action in cumulus cells: implications for oocyte development and in vitro fertilization	Int J Mol Sci	Ineligible publication type
Arakelyan	2025	The identification of genetic factors contributing to human oocyte maturation arrest using whole-genome sequencing	Russian Journal of Genetics	Ineligible cell population
Aydin	2025	Transcriptomic landscape of human oocytes and trophectoderm cells following ultra-rapid or standard vitrification	Hum Rep	Ineligible publication type, ineligible study design
Zhang	2025	Single-cell proteomic analysis of protein alterations in human oocytes undergoing in Vitro aging	Hum Rep	Ineligible publication type
Erdmann	2025	ADR-2 regulates the germline transcriptome and affects fertility and oocyte fate	JBC	Ineligible publication type, ineligible species
Zhang	2025	The cohesin-associated protein Pds5A governs the meiotic spindle assembly via deubiquitination of Kif5B in oocytes	Sci Adv	Ineligible species

Lee	2025	Novel BRCA1-PLK1-CIP2A axis orchestrates homologous recombination-mediated DNA repair to maintain chromosome integrity during oocyte meiosis	NAR	Ineligible species, ineligible method (no sequencing and MS involved)
Liu	2024	Chemically induces proximity reveals a Piezo-dependent meiotic checkpoint at the oocyte nuclear envelope	Science	Ineligible species, ineligible method (no sequencing and MS involved)
Esbert	2024	Oocyte rescue in-vitro maturation does not adversely affect chromosome segregation during the first meiotic division	RBMO	Ineligible study design

Supplementary Table S2. Summary of the human ovarian omic-based studies across the female lifespan

	**Reference**	**Sample information**					Number of samples/cells	Method	Data availability
	(Diedrichs *et al.*, 2012)	Age (number of samples)	Sample type	Characteristics (hormonal treatment)	Omic(s) profiled	Resolution			
		10 WG	FGC	Pregnancy termination reason U	transcriptome	bulk	500 cells	microarray	N/A
	(Williams *et al.*, 2015)	16 WG (1)<br>19 WG (2)<br>21 WG (1)<br>23 WG (1)	ovarian tissue	Pregnancy termination for non-medical reasons	miRNAome	bulk	13 samples	RNA-seq	BioProject: PRJNA292450
	(Del Valle *et al.*, 2017)	6 PCW (1)<br>7 PCW (1)<br>8 PCW (1)<br>8.5-9 PCW (1)<br>9-10 PCW (1)	ovarian tissue	Pregnancy termination reason U	transcriptome	bulk	10 samples	microarray	Array Express: E-MTAB-5525
Fetal	(Wang *et al.*, 2022)	7 WG (2)<br>13 WG (1)<br>16 WG (1)<br>18 WG (1)<br>22 WG (2)	ovarian tissue	Pregnancy termination reason U	transcriptome	single-cell	26,000 cells	10X Genomics Chromium	GSA: HRA000344
	(Li *et al.*, 2017)	5 WG (1)<br>7 WG (1)<br>8 WG (1)<br>10 WG (1)<br>11 WG (1)<br>12 WG (1)<br>14 WG (1)<br>18 WG (2)<br>20 WG (2)<br>23 WG (2)<br>24 WG (2)<br>26 WG (1)	FGCs, somatic cells	Pregnancy termination for medical reasons (e.g., CI, infection, PE)	transcriptome	single-cell	1,099 cells:<br><br>FGC (732)<br><br>Somatic cells (367)	Modified Smart-seq2 (UMI incorporated)	GEO: GSE86146
	(Bothun *et al.*, 2018)	47 dd (1)<br>108 dd (1)<br>122 dd (1)<br>137 dd (1)	ovarian tissue	Pregnancy termination reasons U	proteome	bulk	5 samples	LC-MS/MS	N/A

	(Fowler *et al.*, 2009)	18 WG (2)<br>20 WG (1)	ovarian tissue	Pregnancy termination reasons U	transcriptome	bulk	6 samples	microarray	N/A
	(Lecluze *et al.*, 2020)	8-14 WG (U)<br>13-14 PCW (U)<br>17 PCW (U)	ovarian tissue	Pregnancy termination for non-medical reasons	transcriptome	bulk	24 samples	RNA-seq	GEO: GSE116278
	(Mamsen *et al.*, 2017)	5-6 PCW (7)<br>7 PCW (5)<br>8 PCW (6)<br>9 PCW (1)	ovarian tissue	Pregnancy termination for non-medical reasons	transcriptome	bulk	19 samples	microarray	N/A
	(Taelman *et al.*, 2024)	6-18 WPF (11)	ovarian tissue	Pregnancy termination for non-medical reasons	transcriptome	single-cell	21,049 cells:<br><br>10,284 cells/first trimester<br>10,765 cells/second trimester	10X Genomics Chromium	GEO: GSE181558
	(Guo *et al.*, 2015)	4 WG (1)<br>7 WG (3)<br>10 WG (1)<br>11 WG (2)<br>19 WG (2)	FGC, somatic cells	Pregnancy termination for non-medical reasons	transcriptome, methylome	single-cell	<u>scRNA-seq</u>:<br><br>FGC (84)<br>Somatic cells (38)<br><br><u>Methylome:</u><br>FGC (4)<br>Somatic cells (1)	<u>Methylome:</u><br><br>WGBS<br><br><u>scRNA-seq</u>	GEO: GSE63818
	(Chitiashvili *et al.*, 2020)	4-16 WPF	ovarian tissue	Pregnancy termination reasons U	transcriptome	single-cell	25,000 cells/5 donors	10X Genomics Chromium	GEO: GSE143380
	(Hourmand *et al.*, 2009)	9-20 WG	ovarian tissue	Pregnancy termination for non-medical reasons	transcriptome	bulk	17 samples	microarray	GEO: GSM38700, GSM387038
	(Gkountela *et al.*, 2015)	53 dpf (2)<br>57 dpf (1)<br>67 dpf (1)<br>93 dpf (1)<br>94 dpf (1)<br>108 dpf (1)<br>110 dpf (1)<br>113 dpf (1)	FGC	Pregnancy termination for non-medical reasons	transcriptome	bulk	<u>RNA-seq:</u><br>9 samples<br><br><u>Methylome:</u><br>4 samples	RNA-seq, WGBS	GEO: GSE63392 (only RNA-seq data)

	(Garcia-Alonso *et al.*, 2022)	6-21 (PCW):<br><br>Week 6 (1)<br>Week 7.5 (1)<br>Week 8.4 (1)<br>Week 8.6 (4)<br>Week 8.8 (1)<br>Week 9 (3)<br>Week 10 (1)<br>Week 11 (3)<br>Week 12 (1)<br>Week 13 (3)<br>Week 14 (4)<br>Week 16 (2)<br>Week 17 (2)<br>Week 18 (1)<br>Week 19 (1)<br>Week 20 (1)<br>Week 21 (1)	Ovarian tissue	Pregnancy termination reasons U	transcriptome spatial transcriptome chromatin accessibility	single-cell	scRNA-seq: (cells/individ.):<br><br>153350/28:<br>Week 6: 1598/1<br>Week 7.5: 4213/1<br>Week 8.4: 1192/1<br>Week 8.6: 19901/4<br>Week 8.8: 9242/1<br>Week 9: 15837/3<br>Week 10: 10948/1<br>Week 11: 8582/3<br>Week 12: 10372/1<br>Week 13: 19609/3<br>Week 14: 15434/4<br>Week 16: 15274/2<br>Week 17: 19131/2<br>Week 18: 2017/1<br><br>scATAC-seq: (cells/individ.)<br>58684/8<br>Week 8.6: 3458/1<br>Week 8.8: 8668/1<br>Week 9: 4878/1	scRNA-seq: 10X Genomics Chromium<br><br>scATAC-seq and multimodal snRNA-seq/scATAC-seq: Chromium Single Cell ATAC; Chromium Single Cell Multiome ATAC + Gene Expression<br><br>Spatial transcriptomics: 10X Genomics Visium	Array Express: E-MTAB-10551

							Week 13: 8901/1<br>Week 14: 22479/1<br>Week 18: 5326/1<br>Week 21: 4974/1<br><br><u>Multiome:</u><br>(cells/individ.)<br>33062/5<br>Week 8.6: 12651/1<br>Week 13: 4882/1<br>Week 14: 1645/1<br>Week 20: 7584/1<br>Week 21: 6300/1<br><br><u>Spatial transcriptomics:</u><br>4 samples<br>(Week 11, 14, 17, 19)		
	(McGlacken-Byrne *et al.*, 2025)	7.5-8 PCW (5)<br>9-10 PCW (5)<br>11-12 PCW (5)<br>12 PCW (1)<br>13 PCW (1)<br>15-16 PCW (4)	ovarian tissue	Pregnancy termination reason U	transcriptome	bulk, single-cell	Bulk transcriptome: 19 samples<br><br>snRNA-seq: 10,291 cells/2	RNA-seq, 10X Genomics Chromium	ArrayExpress/Biostudies:<br>S-BSST693 (bulk RNA-seq)<br>S-BSST1194 (snRNA-seq)

Prepuberty	(Ouni *et al.*, 2022)	Prepubertal:<br>7±3 y.o.,<br><br>Reproductive age:<br>26±5 y.o.,<br><br>Menopausal:<br>59±8 y.o.,	ovarian tissue	surgery for benign gynaecological conditions not affecting the ovaries	proteome	bulk	15 samples	LC-MS/MS	PRIDE:<br><br>username:<br>reviewer_pxd025940@ebi.ac.uk;<br>Password:<br>0K9w3nwz
	(Rooda *et al.*, 2024)	Prepubertal:<br>1-11 y.o., (5)<br>Reproductive age:<br>19-29 y.o. (4)<br><br>Validation set (sequencing):<br>Prepubertal (1)<br>Reproductive age (4)<br><br>RNA FISH:<br>Reproductive age (7)<br>Prepubertal (5)	Follicles isolated from cryopreserved cortex tissue	Prepubertal:<br>FP patients<br><br>Reproductive age:<br>GRP patients (androgen treatment)	transcriptome, miRNAome	single follicle	RNA-seq:<br>120 follicles (60/group) → 109 follicles post-QC<br><br>miRNA-seq:<br>120 follicles (60/group) → 113 follicles post-QC	RNA-seq, miRNA-seq	GEO:<br>GSE241981, GSE241982 (RNA-seq)<br>GSE241983 (miRNA-seq)
	(Kavarthapu *et al.*, 2024)	Prepubertal:<br>CG patients (5)<br>Controls (5)	flash frozen ovarian tissue	FP patients	transcriptome, spatial transcriptome	single-cell	snRNA-seq:<br>81,603 nuclei post-QC (39,733 nuclei/CG patients; 41,807 nuclei/controls)<br><br>Spatial transcriptome:<br>6 samples	snRNA-seq:<br>10X Genomics Chromium (v.3.1)<br><br>Spatial transcriptome:<br>10X Genomics Visium	GEO:<br>GSE267932 (snRNA-seq; 9 patients: 5 CG, 4 controls)<br>GSE268523 (spatial transcriptome; 6 patients: 3 CG, 3 controls)

Reproductive age	(Hu *et al.*, 2022)	< 35 y.o.	GV oocytes, MII oocytes, IVM MII oocytes	ART patients (COS, agonist and antagonist)	transcriptome, translatome	mini-bulk, single-cell	10-GV oocytes mini-bulks (20); single GV oocytes (3);<br><br>10-MII oocytes mini-bulks (20); single GV oocytes (3); single IVM oocytes (14)	T&T-seq	GEO: GSE197578
	(Yu *et al.*, 2022)	younger group 25-34 y.o. (42) older group 35-44 y.o. (47)	GC	ART patients (COS antagonist)	transcriptome	bulk	10 samples (5/group)	RNA-seq	Available upon request
	(Li *et al.*, 2020)	27 y.o. (1) 29 y.o. (2) 31 y.o (1) 33 y.o. (2)	Maturation-normal oocytes (3) Maturation-deficient oocytes (3)	IVF patients (COS, agonist)	transcriptome	single-cell	6 oocytes (3/group)	SMARTer-seq	N/A
	(Battaglia *et al.*, 2016)	younger group 28-35 y.o. (3) older group 38-40 y.o. (3)	MII oocytes	ICSI patients (COS, agonist)	miRNAome	bulk	12 samples (6/group: 2/patient)	microarray	N/A
	(Ernst *et al.*, 2018)	26 y.o. (1) 34 y.o. (2)	GC from primordial and primary follicles	FP patients with ovary-unrelated malignant diseases (no HRT)	transcriptome	bulk	12 samples (4/patient)	RNA-seq	N/A
	(Xu *et al.*, 2016)	22 y.o. (1) 27 y.o. (1) 31 y.o. (1)	ovarian tissue	cadavers (no information on HRT)	microRNAome	bulk	3 samples	small RNA-seq	N/A
	(Assou *et al.*, 2013, 201)	U	MII oocytes, CC	ART patients (no information on HRT)	microRNAome	bulk	MII oocytes (10) CC (10)	microarray	N/A
	(Al-Edani *et al.*, 2014)	< 30 y.o., (U) 31-34 y.o., 35-36 y.o. (U) > 37 y.o. (U)	CC	patients with unexplained or male-related infertility	transcriptome	bulk	28 samples	microarray	N/A

				(COS, antagonist)					
	(Tong *et al.*, 2014)	29.1±2.7	corona radiata cells, CC	patients<br>with male-related infertility<br>(COS, agonist + FSH)	miRNAome	bulk	U	RNA-seq	Array Express: E-MTAB-2264
	(Velthut-Meikas *et al.*, 2013)	Study group:<br>31.3±3.1 (3)<br><br>Control group:<br>31.0±6.2 (8)	MGC, CC	ICSI patients with male-related infertility or tubal infertility<br>(COS, antagonist)	transcriptome, miRNAome	bulk	Transcriptome:<br>6 samples<br>(3/MGC, 3/CC)<br>miRNAome:<br>12 samples<br>(6/MGC, 6/CC)	RNA-seq, small RNA-seq	GEO: GSE46490
	(Reyes *et al.*, 2017)	Younger group<br><30 y.o.<br>20 y.o. (1)<br>27 y.o. (1)<br>29 y.o. (3)<br><br>Older group<br>≥40 y.o.<br>40 y.o. (2)<br>42 y.o. (1)<br>43 y.o. (2)	GV oocytes, IVM MII oocytes	IVF patients<br>(COS with FSH and hCG)	transcriptome	single-cell	GV oocytes<br>(10; 1/patient)<br><br>IVM MII oocytes<br>(10; 1/patient)	RNA-seq	N/A
	(Yan *et al.*, 2021)	19 y.o. (1)<br>27 y.o. (1)<br>28 y.o. (1)<br>34 y.o. (1)<br>38 y.o. (1)	Growing oocytes, Mature oocytes, Somatic cells	Oophorocystectomy patients for ovarian cyst removal<br>(no HRT)	transcriptome, methylome, chromatin accessibility	single-cell	DNA<br><br>Oocytes:<br>Growing (86)<br>Fully grown (81)<br>MI (155)<br>MII (90)<br><br>GC (93)<br>Stroma cells (374)<br>Immune cells (20)<br><br>RNA<br>MII oocytes (42)	sc-CHARMseq	GEO: GSE154762

	(Xu *et al.*, 2011)	GV oocyte donors<br>30.65 ±3.87<br><br>MII oocyte donors<br>32.15 ±4.63	GV oocytes, MII oocytes, IVM MII oocytes	ICSI patients<br>(COS, FSH + hCG)	miRNAome	bulk	GV oocytes (60)<br><br>MII oocytes (60)	microarray	N/A
	(Yerushalmi *et al.*, 2014)	34.9 ± 1 (5)	Expanded CC from MII oocytes<br><br>Compact CC from GV oocytes	ART patients<br>(COS, long agonist or short antagonist)	transcriptome	bulk	Expanded CC from MII oocytes<br>(9/3 patients)<br><br>Compact CC from GV oocytes<br>(7/2 patients)	RNA-seq	GEO: GSE50174
	(Guahmich *et al.*, 2023)	21-31 y.o.	ovarian tissue	braindead organ donors,<br>FP patients<br>(no information on HRT)	transcriptome, chromatin accessibility	single-cell	scRNA-seq:<br>48,147 cells post-QC<br><br>scATAC-seq:<br>5,024 cells post-QC	scRNA-seq:<br>10x Genomics Chromium<br>scATAC-seq:<br>10x Genomics Chromium Next GEM Single Cell Multiome	GEO: GSE192722
	(Andrei *et al.*, 2019)	24-38 y.o.	CG,<br>CC	IVF patients for male infertility<br>(COS, GnRH + hCG)	miRNAome	bulk	6 samples<br>(2/individual)	RNA-seq	GEO: GSE106776
	(Jiang *et al.*, 2021)	18-50 y.o.	GC	ART patients<br>(COS)	transcriptome, mRNA methylome	bulk	U	RNA-seq,<br>MeRIP-seq	N/A
	(Dong *et al.*, 2019)	26 y.o. (1)<br>28 y.o. (1)	GC	ART patients<br>(COS, antagonist +hCG)	transcriptome	single-cell	~700 cells<br>(exact number U)	10X Genomics Chromium Drop-seq	N/A
	(Wu, Li, *et al.*, 2022)	< 30 y.o. (U)<br>30-35 y.o. (U)<br>36-39 y.o. (U)<br>≥40 y.o. (U)	GV oocytes, MII oocytes	U<br>(COS, FSH + hCG)	transcriptome, mRNA degradome	single-cell	GV oocytes<br>(12; 3/group)<br><br>MII oocytes<br>(12; 3/group)	Smart-seq2	GEO: GSE173598
	(Hurwitz *et al.*, 2010)	Younger	GC	IVF patients for male factor, tubal factor	transcriptome	bulk	6 samples<br>(3/group)	microarray	N/A

		group<br>≤30 y.o. (3)<br>Older<br>Group<br>≥40 y.o. (3)		(COS, agonist or antagonist)					
	(Caponnetto *et al.*, 2022)	Younger group<br>29.3 ± 2.2 (3)<br>Older group<br>42.2 ± 1.5 (3)	GC from MII oocytes	ART Patients<br>(COS, agonist + FSH)	miRNAome	bulk	12 samples (6/group)	RNA-seq	N/A
	(Smits *et al.*, 2021)	25-30 y.o (10)<br>31-35 y.o. (11)<br>36-38 y.o. (8)<br>39-43 y.o. (9)	GV oocytes	ICSI patients<br>(COS)	transcriptome	bulk	38 samples	microarray	N/A
	(Huang *et al.*, 2023)	Younger group<br>≤31 y.o. (U)<br><br>Older group<br>≥38 y.o. (U)	GV oocytes	ICSI patients for male factor, tubal factor<br>(COS, agonist + hCG)	transcriptome, translatome, mRNA methylome	single-cell	6 oocytes (3/group)	T&T-seq	N/A
	(Ruth *et al.*, 2021)	U	MII oocytes	patients with infertility (no information on HRT)	transcriptome	single-cell	22 oocytes	SMART-seq	EGA: EGAS00001004947
	(Chen, Li, *et al.*, 2022)	Younger group<br><35 y.o. (6)<br><br>Older group<br>>40 y.o. (3)	GC	IVF/ICSI patients (no information on HRT)	transcriptome	bulk	9 samples	RNA-seq	CNCB: PRJCA010954
	(Guo *et al.*, 2022)	U	GV oocytes, MII oocytes, IVM MII oocytes	ART patients<br>(COS, agonist or antagonist)	proteome	single-cell	GV oocytes (12/6 donors)<br>MII oocytes (12/8 donors)<br>IVM MII oocytes (12/2 donors)	LC-MS/MS	PRIDE: PXD023366
	(Battaglia *et al.*, 2017)	≤38 y.o.	MII oocytes<br><br>FF	ICSI patients (COS)	miRNAome	bulk	MII oocytes (6)<br><br>FF (6)	RNA-seq	GEO: GSM2587255

	(Burnik Papler *et al.*, 2015)	< 35 y.o.	CG,<br>CC	IVF patients for tubal factor (COS)	transcriptome	bulk	GC (34)<br>CC (30)	microarray	GEO: GSE55654
	(Diez-Fraile *et al.*, 2014)	Younger group<br><31 y.o. (3)<br>Older group<br>>38 y.o. (3)	FF	ART patients for male factor (COS, long or short, agonist or antagonist)	transcriptome	bulk	6 samples	targeted sequencing	N/A
	(Fan *et al.*, 2021)	U	Oocytes, somatic cells from small AF	FP patients for oncology (2)<br>sickle cell disease (2)<br>beta-thalassemia (1)<br>(no information on HRT)	transcriptome	single-cell	Oocytes after QC (9)<br><br>Somatic cells (113)	Smart-seq2	GEO: GSE186504
	(Wagner *et al.*, 2020)	GRP patients: 20-38 y.o.<br><br>C-sec patients: 28-37 y.o.	ovarian cortex tissue	GRP patients (1) (androgen treatment)<br><br>C-sec patients (3) (no information on HRT)	transcriptome	single-cell	25,131 cells (24,329 after QC)	10X Genomics Chromium<br><br>Smart-seq2	10X data:<br><br>ArrayExpress: E-MTAB-8381<br><br>Smart-seq2 data:<br><br>ArrayExpress: E-MTAB-8403
	(Barone *et al.*, 2020)	Younger group<br><35 y.o. (9)<br><br>Older Group<br>≥35 y.o. (11)	Oocytes,<br>CC	IVF patients for male factor, tubal factor (COS, agonist)	genome, transcriptome	DNA:<br><br>single-cell<br><br>RNA:<br><br>bulk	RNA: 40 samples<br><br>DNA: U	acGHarray, RNA-seq	RNA<br><br>NCBI: SRP156824<br><br>DNA<br>N/A
	(Fan *et al.*, 2019)	U	ovarian medulla tissue	Cancer patients with elective ovariectomy (no information on HRT)	transcriptome	single-cell	56,206 cells/31 samples (20,676 after QC)	10X Genomics Chromium	GEO: GSE118127

	(Ghevaria *et al.*, 2022)	25-44 y.o.	GV oocytes, MI oocytes, MII oocytes + 1st polar body	ART patients: PGT patients with reasons U (no information on HRT)	genome	single-cell	GV oocytes (73) MI oocytes (35) MI oocytes (4) MII oocytes+1st polar body (28)	Repro-seq<br><br>Veriseq	N/A
	(Yu *et al.*, 2015)	Younger group: 26 ± 2.2 Older group: 40 ± 2.3	GC	oocyte donors, ART patients (COS, standard)	transcriptome, methylome	bulk	RNA: 12 samples<br><br>Methylome: 8 samples	RNA-seq, MethylCap-seq, RRBS	RNA: GEO: GSE62093<br><br>Methylome: GEO: GSE63470
	(Chen, Ding, *et al.*, 2022)	20-29 y.o. (22) 30-39 y.o. (14) 40-49 y.o. (37) 50-59 y.o. (61) 60-69 y.o. (42) 70-79 y.o. (4)	ovarian tissue	GTEx database individuals (cadaveric ovaries, collected within 24h of cardiac cessation) (no information on HRT)	transcriptome	bulk	180 samples	RNA-seq	GTEx V8 dataset
	(Grøndahl *et al.*, 2010)	Younger group: ≤35 y.o. (10) Older Group: 37-39 y.o. (11)	MII oocytes	ART Patients (COS)	transcriptome	bulk	15 samples (10/younger group; 5/older group)	microarray	MIAMIexpress: E-MEXP-2347
	(Ouni *et al.*, 2019)	30 y.o. (1) 49 y.o. (1) 59 y.o. (1)	ovarian tissue	patients with gynaecological disease unrelated to ovary (no information on HRT)	proteome	bulk	6 samples	LC-MS/MS	PRIDE: PXD008183
	(Yi *et al.*, 2018)	24 y.o. (1) 25 y.o. (1) 26 y.o. (2) 28 y.o. (1) 31 y.o. (1) 34 y.o. (1)	oocytes, GC	GRP patients (1) Fertility preservation patients (6) (no HRT)	transcriptome	GC: mini-bulk<br><br>oocytes: single-cell	Oocytes (83)<br><br>GC (92 samples; 10 cells/follicle/ mini-bulk)	RNA-seq	GEO: GSE107746

	(Shi *et al.*, 2022)	Younger group <36 y.o. (17) Older Group >36 y.o. (3)	FF	IVF patients (COS, hCG)	transcriptome	bulk	U	SMARTer-seq	NCBI: PRJNA879096
	(Wu, Zhu, *et al.*, 2022)	29.67±4	FF, CC from matched pre-ovulatory follicle	ICSI patients for male factor (COS, agonist or antagonist)	transcriptome	single-cell	15,865 cells (14,592 after QC)	RNA-seq	GEO: GSE189960
	(Méar *et al.*, 2023)	Tissue microrray 21 y.o. (1) 22 y.o. (1) 30 y.o. (1) 35-47 y.o. (9) 55-86 y.o. (7) Transciptomic public data	ovarian tissue	GRP patients Tissue donors (no information on HRT)	transcriptome, proteome	single-cell, bulk	Transcriptome: 42,509 integrated cells Proteome: U	tissue microarray	N/A
	(Liu *et al.*, 2023)	30-39 y.o. (14) 40-49 y.o. (37) 50-59 y.o. (61) Transciptome: public data	ovarian tissue	GTEx database individuals (Cadaveric ovaries, collected within 24h of cardiac cessation) (no information on HRT)	transcriptome	bulk, single-cell	112 bulk samples *N* of single cells used in analysis= U	RNA-seq	GTEx V8 dataset, GEO: GSE118127
	(Olsen *et al.*, 2020)	25-29 y.o. (U) 30-34 y.o. (U) 35-39 y.o. (U) 40-44 y.o. (U)	mural GC	ART patients (COS)	methylome	bulk	63 samples (59 after QC)	EPIC array	N/A
	(Ye *et al.*, 2020)	U	MII oocytes, IVM MII oocytes	IVF patients (COS, antagonist), C-sec patients	transcriptome, methylome, genome	single-cell	MII oocytes (8) IVM oocytes (7)	scTrio-seq	N/A
	(Kristensen *et al.*, 2015)	24-34 y.o.	Preantral follicles	FP patients for cancer (12) other reasons (3)	transcriptome	bulk	109 follicles/17 pools/8 different patients	microarray	ArrayExpress: E-MEXP-3783

				(no information on HRT)					
	(Yu *et al.*, 2020)	27 y.o. (1)<br>28 y.o. (2)<br>31 y.o. (2)<br>32 y.o. (1)<br>33 y.o. (1)<br>34 y.o. (1)<br>35 y.o. (1)<br>36 y.o. (1)<br>37 y.o. (1)<br>38 y.o. (2)<br>39 y.o. (1)<br>40 y.o. (1)<br>41 y.o. (2)	GV oocytes, MI oocytes, MII oocytes	oocyte donors (no information on HRT)	transcriptome, methylome	bulk	<u>Methylome:</u><br>GV oocytes (12)<br>MI oocytes (10)<br>MII oocytes (10)<br><br><u>Transcriptome:</u><br>GV oocytes (7)<br>MI oocytes (7)<br>MII oocytes (7)	WGBS, RNA-seq	WGBS:<br><br>GEO: GSE71985<br><br>RNA-seq:<br><br>N/A
	(Ernst *et al.*, 2017)	U	Oocytes from primordial follicles, oocytes from primary follicles	FP patients (no HRT)	transcriptome	bulk	6 samples (pooled data from primordial oocytes (436), primary oocytes (182)	RNA-seq	N/A
	(Yoo *et al.*, 2011)	18-29 y.o.	FF,<br>GC	IVF patients for male infertility,<br>oocyte donors (COS)	transcriptome, proteome	bulk	U	LC-MS/MS, microarray	N/A
	(Zhao *et al.*, 2019)	U	MII oocytes, IVM MII oocytes	Oocyte donors (no information on HRT)	transcriptome	single-cell	6 oocytes (3/group)	RNA-seq	GEO: GSE110798
	(Barragán *et al.*, 2017)	20-35 y.o.	MII oocytes	IVF patients (COS, hMG+antagonist)	transcriptome	bulk	36 samples	microarray	GEO: GSE87201
	(Yu *et al.*, 2017)	28 y.o. (1)<br>29 y.o. (1)<br>34 y.o. (1)<br>35 y.o. (1)<br>36 y.o. (2)<br>37 y.o. (1)<br>38 y.o. (2)<br>40 y.o. (2)<br>41 y.o. (1)<br>42 y.o. (1)	GV oocytes, MI oocytes, MII oocytes	ART patients (COS, antagonist or agonist flare, luteal lupron)	methylome	single-cell	GV oocytes (10/7 patients)<br><br>MI oocytes (10/4 patients)<br><br>MI oocytes (10/4 patients)	sc-WGBS	GEO: GSE71985

	(Wyse *et al.*, 2020)	33.9 ±1.6	CC from GV oocytes,<br>CC from MII oocytes	ART Patients<br>(COS, antagonist)	transcriptome	bulk	CC from GV oocytes (11)<br>CC from MII oocytes (11)	SMART-seq v4	N/A
	(Takeuchi *et al.*, 2022)	30 y.o. (1)<br>31 y.o. (3)<br>33 y.o. (1)<br>34 y.o. (2)<br>35 y.o. (1)<br>36 y.o. (1)<br>37 y.o. (1)<br>39 y.o. (1)	IVM-normal:<br>GV oocytes,<br>MI oocytes,<br>MII oocytes<br><br>IVM-delayed:<br>GV oocytes,<br>MI oocytes,<br>MII oocytes	ART patients<br>(COS)	transcriptome	single-cell	IVM-normal:<br>GV oocytes (4)<br>MI oocytes (3)<br>MII oocytes (3)<br><br>IVM-delayed:<br>GV oocytes (3)<br>MI oocytes (3)<br>MII oocytes (3)	Smart-seq2	GEO: GSE166533
	(Llonch *et al.*, 2021)	18 y.o. (1)<br>19 y.o. (1)<br>20 y.o. (5)<br>22 y.o. (1)<br>23 y.o. (2)<br>24 y.o. (3)<br>25 y.o. (2)<br>27 y.o. (1)<br>28 y.o. (3)<br>31 y.o. (1)<br>32 y.o. (3)<br>33 y.o. (3)<br>34 y.o. (2)<br>36 y.o. (2)<br>37 y.o. (1)<br>38 y.o. (1)<br>40 y.o. (2)<br>42 y.o. (1)<br>43 y.o. (2)	GV oocytes,<br>IVM MII oocytes	ART patients (12),<br>oocyte donors (25)<br>(COS, antagonist + hCG)	transcriptome	single-cell	GV oocytes<br>(40; 35 after QC)<br><br>IVM MI oocytes<br>(32; 31 after QC)	Smart-seq2	N/A
	(Hou *et al.*, 2013)	25-35 y.o.	polar bodies, pronucleus from fertilized oocytes	oocyte donors<br>(COS, hGC)	genome	single cell	183 cells	WGS	NCBI: SRA091188

	(Ouandaogo *et al.*, 2012)	< 36 y.o.	CC from GV oocytes,<br>CC from MI oocytes,<br>CC from MII oocytes	ART patients (COS, agonist or antagonist)	transcriptome	bulk	24 samples	microarray	GEO: GSE31681
	(Molinari *et al.*, 2016)	Younger group<br>≤30y.o. (U)<br>Older group<br>>40y.o. (U)	CC from MII oocytes	ART patients (COS, agonist or antagonist)	transcriptome	bulk	20 samples (10/group)	RNA-seq	N/A
	(Markholt *et al.*, 2012)	11 y.o. (1)<br>21 y.o. (1)	Primordial, intermediate, primary follicles	FP patients<br>(no information on HRT)	transcriptome	bulk	3 samples	microarray	MIAMIexpress: E-MEXP-3454
	(Pietroforte *et al.*, 2023)	18-34 y.o.	GV oocytes,<br>FTM GV oocytes,<br>MII oocytes,<br>IVM MII oocytes	oocyte donors<br>(COS, antagonist + agonist)	transcriptome	single-cell	GV oocytes (10)<br>FTM GV oocytes (10)<br>MII oocytes (10)<br>IVM MII oocytes (10)	RNA-seq	GEO: GSE213267
	(Yuan *et al.*, 2021)	Younger group<br><30y.o. (U)<br>Older group<br>≥40y.o. (U)	MII oocytes	ICSI patients<br>(COS, antagonist)	transcriptome	single-cell	12 oocytes (6/group)	Smart-seq2	GEO: GSE155179
	(Virant-Klun *et al.*, 2016)	U	GV oocytes,<br>MI oocytes,<br>MII oocytes	IVF patients<br>(COS)	proteome	single-cell	Immature oocytes (100)<br>MII oocytes (100)	LC-MS/MS	PRIDE: PXD004142
	(Pla *et al.*, 2021)	Younger group:<br>25.6±6.1<br><br>Older group:<br>31.4±1.8	FF from hSAF,<br>FF from MII oocytes,<br>FF from maturation-deficient IVM oocytes	FP patients for<br>cancer (24)<br>lupus (1)<br>multiple sclerosis (1)<br>(no information on HRT)	proteome	bulk	36 samples	LC-MS/MS	PRIDE: PXD013344, PXD021338

	(Cadenas *et al.*, 2023)	< 36 y.o.	FF, GC prior to oocyte pick-up<br><br>FF, GC at oocyte pick-up	ART Patients<br>(COS, FSH+agonist)	transcriptome, proteome	bulk	U	microarray<br><br>LC-MS/MS	RNA:<br>GEO:<br>GSE133868<br><br>Proteome:<br>N/A
	(Yang *et al.*, 2019)	U	GV oocytes, MII oocytes	U<br>(COS, agonist + hCG)	miRNAome	single-cell	U	CAS-seq	GEO:<br>GSE95218
	(Ntostis *et al.*, 2021)	younger group<br><30y.o.<br><br>older group<br>>40 y.o.	GV oocytes, MII oocytes	IVF patients<br>(COS, agonist + FSH)	transcriptome	single-cell	GV oocytes<br>(10; 5/group)<br><br>MII oocytes<br>(11; 5/younger group; 6/older group)	SMART-seq v4	GEO:<br>GSE164371
	(Smits *et al.*, 2023)	23-42 y.o.	GV oocytes, CC,<br>MII oocytes	ICSI patients for male factor, tubal factor<br><br>FP for social reasons<br>(no information on HRT)	metabolome, lipidome	bulk	Oocytes<br>(150; 10 oocytes/group in triplicate)	LC-MS/MS	N/A
	(Jia *et al.,* 2023)	≥35 y.o.<br>≤42 y.o.	GC	ART<br>patients (COS, antagonist or agonist)	transcriptome	bulk	U	RNA-seq	GEO:<br>GSE232306
	(Wu *et al.*, 2024)	Young group:<br>18 y.o. (1)<br>19 y.o. (1)<br>22 y.o. (1)<br>28 y.o. (1)<br>29 y.o. (1)<br><br>Middle-age group:<br>36 y.o. (1)<br>37 y.o. (1)<br>38 y.o. (1)<br>39 y.o. (2)	ovarian tissue	Oophorocystectomy patients with non-ovarian indications<br>(no HRT)	transcriptome, spatial transcriptome	single-cell, bulk	transcriptome:<br>9 samples<br>(3/group)<br>92,965 cells<br><br>spatial transcriptome:<br>15 samples<br>(5/group)	scRNA-seq:<br>10X Genomics Chromium<br><br>Spatial:<br>10X Genomics Visium	GEO:<br>GSE255690

		Older age group:<br>47 y.o. (1)<br>48 y.o. (1)<br>49 y.o. (3)							
	(Jones *et al.*, 2024)	18–37 y.o.	ovarian tissue	deceased organ donors<br>(no information on HRT)	transcriptome, spatial transcriptome	single-cell	transcriptome:<br>21,198 cells/3 donors<br><br>spatial transcriptome:<br>257 regional samples/2 donors	scRNA-seq:<br>10X Genomics Chromium<br><br>Spatial:<br>Nanostring GeoMx DSP	scRNA-seq:<br><br>GEO:<br>GSE260685<br><br>Spatial:<br>GEO:<br>GSE260686
	(Lewandowska *et al.*, 2017)	U	FF	IVF patients for male factor<br>(COS, long agonist protocol: OC pills + hGC)	proteome	bulk	12 samples/4 patients	LC-MS/MS	PRIDE:<br>PXD006550
	(Poulsen *et al.*, 2019)	18-35 y.o. (U)	FF	ART patients for male factor, tubal factor, unexplained infertility<br>(COS, FSH + antagonist)	proteome	bulk	25 samples	LC-MS/MS	PRIDE:<br>PXD012561
	(Cornet-Bartolomé *et al.*, 2021)	21-34 y.o.	GV oocytes, IVM MII oocytes	oocyte donors<br>(COS, antagonist)	transcriptome	bulk	GV oocytes<br>(4)<br>IVM MII oocytes<br>(12)	microarray	GEO:<br>GSE87201
	(Poulsen *et al.*, 2020)	18-35 y.o. (50)	GC	ART patients for male factor, tubal factor, unexplained infertility<br>(COS, FSH + antagonist)	transcriptome	bulk	83 samples	microarray	GEO:<br>GSE13386
	(Rodríguez-Nuevo *et al.*, 2022)	19-34 y.o. (2)	ovarian tissue	Surgical patients for non-ovarian gynaecological conditions	proteome	bulk	4 samples	LC-MS/MS	PRIDE:<br>PXD025369

	(Zhang *et al.*, 2020)	Younger group<br><35 y.o. (3)<br>Older Group<br>≥40 y.o. (3)	MII oocytes	patients with infertility unrelated to ovary (COS, agonist)	transcriptome	single-cell	6 oocytes (3/group)	microarray	N/A
	(Jin *et al.*, 2025)	Younger group<br>23 y.o. (1)<br>27 y.o. (1)<br>28 y.o. (1)<br>29 y.o. (1)<br><br>Older group<br>49 y.o. (1)<br>51 y.o. (1)<br>52 y.o. (1)<br>54 y.o. (1)	ovarian tissue (multiple sites at cortex + medulla)	deceased organ donors (7),<br>GRP patients (1)<br>(no information on HRT)	transcriptome, chromatin accessibility	single-cell	snRNA-seq:<br>42,568 nuclei post-QC<br><br>snATAC-seq:<br>41,550 nuclei post-QC	snRNA-seq:<br>10x Genomics protocol CG000393 (Rev A)<br><br>snATAC-seq:<br>10x Genomics protocol CG000212 (Rev B)	GEO: GSE202601
	(Zhang, Luo, *et al.*, 2025)	23 y.o. (1)<br>26 y.o. (2)<br>28 y.o. (3)<br>30 y.o. (1)<br>31 y.o. (1)<br>33 y.o. (2)<br>35 y.o. (1)	GV oocytes, MI oocytes	ICSI patients for male and tubal factors (COS, agonist)	proteome	single-cell	Proteome:<br>8 GV oocytes/5 patients<br>8 MI oocytes/8 patients	LC-MS/MS	N/A
	(Dhori *et al.*, 2024)	24 y.o. (3)<br>28 y.o. (1)<br>32 y.o. (1)<br>33 y.o. (4)<br>34 y.o. (1)<br>35 y.o. (1)<br>38 y.o. (4)<br>40 y.o. (1)<br>U (28)	MGC,CC, oocytes	IVF patients	transcriptome, miRNAome	bulk	39 samples	RNA-seq, small RNA-seq	BioProject:<br><br>RNA-seq:<br>PRJNA200696<br>PRJNA216966<br>PRJNA649934<br><br>small RNA-seq:<br>PRJNA200699<br>PRJNA417973

	(Galatidou *et al.*, 2024)	Younger group:<br>≤ 30 y.o.<br>24.65±3.19<br>18 y.o. (3)<br>20 y.o. (1)<br>21 y.o. (3)<br>23 y.o. (2)<br>24 y.o. (3)<br>25 y.o. (2)<br>26 y.o. (2)<br>28 y.o. (3)<br>29 y.o. (3)<br>30 y.o. (3)<br><br>Older group:<br>≤ 37 y.o.<br>39±1.7.<br>37 y.o. (5)<br>38 y.o. (7)<br>39 y.o. (5)<br>40 y.o. (2)<br>41 y.o. (3)<br>43 y.o. (2)	GV, IVO-MII oocytes	U	proteome	single-cell	GV oocytes:<br>Younger group 18/18 patients, 12/12 patients post-QC<br><br>Older group 18/18 patients, 14/14 patients post-QC<br><br>IVO-MII oocytes:<br>Younger group 18/9 patients, 17/9 patients post-QC<br><br>Older group 14/7 patients, 11/7 patients post-QC	plexDIA (data-independent MS method for single-cell proteomic quantification)	MassIVE: MSV000093870
	(Yao *et al.*, 2025)	≤ 35 y.o (U)	GV oocytes, MII oocytes	ICSI patients (COS long protocol)	transcriptome, epitranscriptome	single-cell	13 oocytes	C2T-seq, scm^{6}A-seq	GSA: HRA007855 HRA007856
	(Orvieto *et al.*, 2025)	Younger group:<br>23.3±6.6<br>6 y.o.(1)<br>25 y.o.(1)<br>29 y.o.(1)<br><br>Older group:<br>39±1<br>38 y.o.(1)<br>39 y.o.(1)<br>40 y.o.(1)	GV oocytes	FP patients (COS, antagonist + agonist)	transcriptome	single-cell	6 oocytes	RNA-seq (NEBnext)	N/A
	(Cadenas *et al.*, 2025)	19-36 y.o.<br>19 y.o. (1)	CC from fresh GV	FP patients	transcriptome	bulk	CC (fresh GV): 8; 4 post-QC	microarray	Dryad repository:

		24 y.o. (1)<br>25 y.o. (1)<br>27 y.o. (1)<br>31 y.o. (1)<br>33 y.o. (2)<br>35 y.o. (1)	oocytes, GV-IVM, MII-IVM				CC (GV-IVM): 16; 7 post-QC<br>CC (GV-MII): 23; 11 post-QC		doi:10.5061/dryad.66t1g1k9d (non-working link)
	(Zhang *et al.*, 2024)	U	GV oocytes	ART patients<br>(COS, antagonist)	transcriptome	bulk	5-10 oocytes/group	RNA-seq	GSA: HRA006194
	(Zhao *et al.*, 2024)	U	GC	U	transcriptome	bulk	67 samples	RNA-seq	GEO: GSE107746
	(Varik *et al.*, 2025)	U (42)	SEV, LEV from FF	ART patients<br>(COS, antagonist and agonist)	miRNAome	bulk	42 samples ⇒ 7 samples/pool ⇒ 6 pooled samples	small RNA-seq	ENA: PRJEB83132
	(Liu *et al.*, 2025)	Young group:<br><28 y.o. (U)<br>26.7±1.25<br><br>Older group:<br>>40 y.o. (U)<br>42±2	exosomes from FF	IVF patients<br>(no information on HRT)	proteome	bulk	14 samples (7/group)	LC-MS/MS	Proteome XChange: PXD041139 (username: password: Znmwyh6q
	(Zhang, Yang, *et al.*, 2025)	Younger group:<br>21-29 y.o. (10)<br><br>Older group:<br>37-43 y.o. (10)	IVM MII	ICSI patients for male and tubal factors<br>(COS, antagonist)	transcriptome	single-cell	20 oocytes (10-group)	scRNA-seq: SMART-seq2	SRA: PRJNA1213145
	(Gokyer *et al.*, 2024)	Adolescents:<br>10-19 y.o. (19)<br>Reproductive age:<br>22-30 y.o. (19)	CC from MII oocytes	FP patients,<br>oocyte donors<br>(COS, antagonist + hCG or agonist)	transcriptome	bulk	38 samples (19/group)	RNA-seq	GEO: GSE265995
	(Mao *et al.*, 2024)	29 y.o. (1)<br>30 y.o. (1)	GCs from GV and MII oocytes	U<br>(no information on HRT)	transcriptome, 3D genome	single-cell, bulk	scRNA-seq:<br>29 samples<br><br>Hi-C:<br>3 samples	scRNA-seq: SMART-seq2<br><br>Hi-C	NCBI: PRJNA1116765 (only scRNA-seq)
	(Wang *et al.*, 2024)	33.02±3.2 (46)	COC	U<br>(COS, long agonist)	transcriptome, miRNAome	bulk	U	RNA-seq, small RNA-seq	SRA: PRJNA955166 (mRNA)<br>PRJNA954349

									(miRNA)
	(Gu *et al.*, 2024)	Young:<br><35 y.o. (30)<br>28.97±0.53<br>Advanced:<br>≥ 40;<br>44.13±0.58	exosomes from FF	ART patients<br>(COS, agonist+GnRH or FSH+antagonist)	metabolome	bulk	U	GC-TOFMS	N/A
	(Caniçais *et al.*, 2025)	20 y.o. (1)<br>22 y.o. (1)<br>23 y.o. (1)<br>24 y.o. (1)<br>25 y.o. (2)<br>29 y.o. (1)<br>30 y.o. (2)<br>33 y.o. (1)	GV, MI, MII oocytes	oocyte donors<br>(COS, FSH + GnRH)	transcriptome	single-cell	GV oocytes (6)<br>MI oocytes (6)<br>MII oocytes (7)	RNA-seq	N/A
	(Rogers *et al.*, 2025)	Young:<br><32 y.o.<br>29 y.o. (1)<br>30 y.o. (3)<br>31 y.o. (5)<br><br>Aged:<br>>38 y.o.<br>39 y.o. (2)<br>40 y.o. (1)<br>41 y.o. (2)<br>42 y.o. (1)<br>43 y.o. (2)<br>45 y.o. (1)	GC	U<br>(no information on HRT)	transcriptome, miRNAome	bulk	transcriptome:<br>GC (6)<br>miRNAome:<br>GC (6)	RNA-seq, small RNA-seq	GEO: GSE270748 (mRNA)<br>GSE270749 (miRNA)
	(Liu *et al.*, 2024)	25-38 y.o. (U)	GV, IVO MII, IVM MII oocytes	ICSI patients for tubal factors<br>(COS)	transcriptome	single-cell	GV (9)<br>IVO MII (4)<br>IVM MII (4)	PAIso-seq	GSA: HRA003115
	(Arbeithuber *et al.*, 2025)	<30 y.o. (8)<br>30-34 y.o. (6)<br>34-42 y.o. (8)	GV, MI, TI, MII oocytes	ICSI patients for male and tubal factors<br>(COS, FSH+hCG)	MT genome	single-cell	GV (4)<br>MI (8)<br>TI (6)<br>MII (62)	targeted mtDNA capture sequencing	SRA: PRJNA1181658
	(Boucret *et al.*, 2017)	25-42 y.o. (35)	53 COC	IVF patients, ICSI patients<br>(COS, agonist or antagonist, FSH or FSH+LH)	MT genome	bulk	53 COC	targeted mtDNA capture sequencing	N/A

Postmenopausal	(Yi *et al.*, 2018)	Pre-menopausal:<br>46 y.o. (1)<br>48 y.o. (1)<br>51 y.o. (1)<br>Post-menopausal:<br>52 y.o. (1)<br>59 y.o. (2)	ovarian tissue	patients with benign ovarian cysts<br>(no steroid hormone drugs ≥ 6 months before the surgery)	proteome, acetyl-proteome	bulk	6 samples	LC-MS/MS	N/A
	(Lengyel *et al.*, 2022)	Post-menopausal:<br>58 y.o. (1)<br>62 y.o. (2)<br>65 y.o. (1)<br>70 y.o. (1)<br>72 y.o. (1)	ovarian tissue	U<br>(no information on HRT)	transcriptome, chromatin accessibility	single-cell	scRNA-seq:<br>26,134 cells/6 donors after QC<br><br>scATAC-seq:<br>18,315 cells/3 donors after QC	scRNA-seq:<br>10X Genomics Chromium<br><br>Drop-seq<br><br>scATAC-seq:<br>10X Genomics Chromium Next GEM	EGA:<br>EGAS00001006780<br>(restricted access)
	(Pei *et al.*, 2024)	32-56 y.o. (U)	ovarian tissue	patients with oophorectomy for benign lesions<br>(no information on HRT)	spatial transcriptome	bulk	10 samples	Spatial:<br>10X Genomics Visium	N/A
	(Ouni *et al.*, 2022)	Reproductive age:<br>26±5 y.o.<br><br>Post-menopausal:<br>59±8 y.o.	ovarian tissue	surgery for benign gynaecological conditions not affecting the ovaries	proteome	bulk	10 samples	LC-MS/MS	PRIDE:<br><br>username:<br>reviewer_pxd025940@ebi.ac.uk;<br>Password:<br>0K9w3nwz
	(Devrukhkar *et al.*, 2025)	50-70 y.o.,<br>(61.3 ± 6.2)<br>U	cortex and medulla explants	patients with salpingo-oophorectomies and/or total laparoscopic hysterectomies<br>(no information on HRT)	transcriptome, proteome	single-cell, bulk	transcriptome:<br>6 day explants:<br>24,702 (cortex treated)<br>11,332 (cortex control)<br>22,852 (medulla treated)	transcriptome:<br>10X Genomics Chromium<br><br>LC-MS/MS	transcriptome:<br><br>SenNet:<br>SNT559.LLTN.426<br>proteome:<br><br>MassIVE:<br>MSV000095932

							12,808 (medulla control)<br>10 day explants:<br>7,207 (cortex treated)<br>7,980 (cortex control)<br>3,774 (medulla treated)<br>5,324 (medulla control)<br>proteome: U		
	(Jain *et al.*, 2024)	50.5±13.6 (157)	ovarian tissue	organ donors (no information on HRT)	methylome	bulk	157 samples	Infinium MethylationEPIC array	Raw data:<br>AnVIL platform: AnVIL_GTEx_V9<br><br>Normalized data:<br>GEO: GSE213478

FGC: fetal germ cells; WG: weeks of gestation; PCW: post-conception weeks; dd: developmental days; WPF: weeks post fertilization; CI: cervical insufficiency; CG: classic galactosemia; PE: pre-eclampsia; COS: controlled ovarian stimualtion; FF: follicular fluid; GC: granulosa cells; MGC: mural granulosa cells; CC: cumulus cells; COC: cumulus oophorus complexes; GRP: gender reassignment patients; C-sec: Caesarean section; FP: fertility preservation; ART: assistive reproductive technology; PGT: preimplantation genetic testing; OC: oral contraceptive; WGS: whole genome sequencing; WGBS: whole genome bisulfite sequencing; UMI: unique molecular identifier; QC: quality control; TI: telophase I; GEO: Gene Expression Omnibus; EGA: European Genome-phenome Archive; ENA: European Nucleotide Archive; NCBI: National Center for Biotechnology Information; GSA: Genome Sequence Archive; SRA: Sequence Read Archive; PRIDE: Proteomics Identification Database; MassIVE: Mass Spectrometry Interactive Virtual Environment; IF: immunofluorescence; siRNA: small interfering RNA; IVO MII: *in vivo* matured MII oocytes; IVM MII: *in vitro* matured MII oocytes; C2T-seq: Cap to Tail sequencing; scm^{6}A-seq: single-cell m^{6}A sequencing; SEV: small extracellular vesicles; LEV: large extracellular vesicles; Hi-C: high-throughput sequencing with chromosome conformation capture; GC-TOFMS: gas chromatography–time of flight mass spectrometry; MT: mitochondrial; PAIso-seq: poly(A) inclusive RNA isoform sequencing; SenNet: The Cellular Senescence Network; U indicates that the parameter is unreported; N/A indicates when data is not available.

References:

Al-Edani T, Assou S, Ferrières A, Bringer Deutsch S, Gala A, Lecellier C-H, Aït-Ahmed O, Hamamah S. Female aging alters expression of human cumulus cells genes that are essential for oocyte quality. *Biomed Res Int* 2014;**2014**:964614.

Andrei D, Nagy RA, van Montfoort A, Tietge U, Terpstra M, Kok K, van den Berg A, Hoek A, Kluiver J, Donker R. Differential miRNA Expression Profiles in Cumulus and Mural Granulosa Cells from Human Pre-ovulatory Follicles. *Microrna* 2019;**8**:61–67.

Arbeithuber B, Anthony K, Higgins B, Oppelt P, Shebl O, Tiemann-Boege I, Chiaromonte F, Ebner T, Makova KD. Allele frequency selection and no age-related increase in human oocyte mitochondrial mutations. *Sci Adv* 2025;**11**:eadw4954.

Assou S, Al-edani T, Haouzi D, Philippe N, Lecellier C-H, Piquemal D, Commes T, Aït-Ahmed O, Dechaud H, Hamamah S. MicroRNAs: new candidates for the regulation of the human cumulus-oocyte complex. *Hum Reprod* 2013;**28**:3038–3049.

Barone S, Sarogni P, Valli R, Pallotta MM, Silvia G, Frattini A, Khan AW, Rapalini E, Parri C, Musio A. Chromosome Missegregation in Single Human Oocytes Is Related to the Age and Gene Expression Profile. *Int J Mol Sci* 2020;**21**:1934.

Barragán M, Pons J, Ferrer-Vaquer A, Cornet-Bartolomé D, Schweitzer A, Hubbard J, Auer H, Rodolosse A, Vassena R. The transcriptome of human oocytes is related to age and ovarian reserve. *MHR: Basic science of reproductive medicine* 2017;**23**:535–548.

Battaglia R, Vento ME, Borzì P, Ragusa M, Barbagallo D, Arena D, Purrello M, Di Pietro C. Non-coding RNAs in the Ovarian Follicle. *Front Genet* 2017;**8**:57.

Battaglia R, Vento ME, Ragusa M, Barbagallo D, La Ferlita A, Di Emidio G, Borzí P, Artini PG, Scollo P, Tatone C, *et al.* MicroRNAs Are Stored in Human MII Oocyte and Their Expression Profile Changes in Reproductive Aging. *Biol Reprod* 2016;**95**:131.

Bothun AM, Gao Y, Takai Y, Ishihara O, Seki H, Karger B, Tilly JL, Woods DC. Quantitative Proteomic Profiling of the Human Ovary from Early to Mid-Gestation Reveals Protein Expression Dynamics of Oogenesis and Folliculogenesis. *Stem Cells and Development* 2018;**27**:723–735.

Boucret L, Bris C, Seegers V, Goudenège D, Desquiret-Dumas V, Domin-Bernhard M, Ferré-L'Hotellier V, Bouet PE, Descamps P, Reynier P, *et al.* Deep sequencing shows that oocytes are not prone to accumulate mtDNA heteroplasmic mutations during ovarian ageing. *Hum Reprod* 2017;**32**:2101–2109.

Burnik Papler T, Vrtacnik Bokal E, Maver A, Kopitar AN, Lovrečić L. Transcriptomic Analysis and Meta-Analysis of Human Granulosa and Cumulus Cells. Feltus FA (ed). *PLoS ONE* 2015;**10**:e0136473.

Cadenas J, Adrados CS, Kristensen SG, Macklon KT, Greve VH, Fedder J, Mamsen LS, Andersen CY. Transcriptional Changes in Cumulus Cells During In Vitro Maturation of Human Oocytes. *Mol Reprod Dev* 2025;**92**:e70035.

Cadenas J, Poulsen LC, Nikiforov D, Grøndahl ML, Kumar A, Bahnu K, Englund ALM, Malm J, Marko-Varga G, Pla I, *et al.* Regulation of human oocyte maturation *in vivo* during the final maturation of follicles. *Human Reproduction* 2023;**38**:686–700.

Caniçais C, Sobral D, Vasconcelos S, Cunha M, Pinto A, Mesquita Guimarães J, Santos F, Barros A, Dória S, Marques CJ. Transcriptomic analysis and epigenetic regulators in human oocytes at different stages of oocyte meiotic maturation. *Dev Biol* 2025;**519**:55–64.

Caponnetto A, Battaglia R, Ferrara C, Vento ME, Borzì P, Paradiso M, Scollo P, Purrello M, Longobardi S, D'Hooghe T, *et al.* Down-regulation of long non-coding RNAs in reproductive aging and analysis of the lncRNA-miRNA-mRNA networks in human cumulus cells. *J Assist Reprod Genet* 2022;**39**:919–931.

Chen L, Ding B, Wu L, Qiu J, Li Q, Ye Z, Yang J. Transcriptome Analysis Reveals the Mechanism of Natural Ovarian Ageing. *Front Endocrinol* 2022;**13**:918212.

Chen P, Li W, Liu X, Wang Y, Mai H, Huang R. Circular RNA expression profiles of ovarian granulosa cells in advanced-age women explain new mechanisms of ovarian aging. *Epigenomics* 2022;**14**:1029–1038.

Chitiashvili T, Dror I, Kim R, Hsu F-M, Chaudhari R, Pandolfi E, Chen D, Liebscher S, Schenke-Layland K, Plath K, *et al.* Female human primordial germ cells display X-chromosome dosage compensation despite the absence of X-inactivation. *Nat Cell Biol* 2020;**22**:1436–1446.

Cornet-Bartolomé D, Barragán M, Zambelli F, Ferrer-Vaquer A, Tiscornia G, Balcells S, Rodriguez A, Grinberg D, Vassena R. Human oocyte meiotic maturation is associated with a specific profile of alternatively spliced transcript isoforms. *Mol Reprod Dev* 2021;**88**:605–617.

Del Valle I, Buonocore F, Duncan AJ, Lin L, Barenco M, Parnaik R, Shah S, Hubank M, Gerrelli D, Achermann JC. A genomic atlas of human adrenal and gonad development. *Wellcome Open Res* 2017;**2**:25.

Devrukhkar PR, Watson MA, Soygur B, Wu F, Bons J, Anvari H, Atazhanova T, Martin N, Tran T, Schneider K, *et al.* A Comprehensive Multiomics Signature of Doxorubicin-Induced Cellular Senescence in the Postmenopausal Human Ovary. *Aging Cell* 2025:e70111.

Dhori X, Gioiosa S, Gonfloni S. An integrated analysis of multiple datasets reveals novel gene signatures in human granulosa cells. *Sci Data* 2024;**11**:972.

Diedrichs F, Mlody B, Matz P, Fuchs H, Chavez L, Drews K, Adjaye J. Comparative molecular portraits of human unfertilized oocytes and primordial germ cells at 10 weeks of gestation. *Int J Dev Biol* 2012;**56**:789–797.

Diez-Fraile A, Lammens T, Tilleman K, Witkowski W, Verhasselt B, De Sutter P, Benoit Y, Espeel M, D'Herde K. Age-associated differential microRNA levels in human follicular fluid reveal pathways potentially determining fertility and success of in vitro fertilization. *Hum Fertil (Camb)* 2014;**17**:90–98.

Dong J-P, Dai Z-H, Jiang Z-X, He Y, Wang L, Liao Q-Y, Sun N-X, Wang Y-N, Sun S-H, Lin W, *et al.* CD24: a marker of granulosa cell subpopulation and a mediator of ovulation. *Cell Death Dis* 2019;**10**:791.

Ernst EH, Franks S, Hardy K, Villesen P, Lykke-Hartmann K. Granulosa cells from human primordial and primary follicles show differential global gene expression profiles. *Hum Reprod* 2018;**33**:666–679.

Ernst EH, Grøndahl ML, Grund S, Hardy K, Heuck A, Sunde L, Franks S, Andersen CY, Villesen P, Lykke-Hartmann K. Dormancy and activation of human oocytes from primordial and primary follicles: molecular clues to oocyte regulation. *Human Reproduction* 2017;**32**:1684–1700.

Fan X, Bialecka M, Moustakas I, Lam E, Torrens-Juaneda V, Borggreven NV, Trouw L, Louwe LA, Pilgram GSK, Mei H, *et al.* Single-cell reconstruction of follicular remodeling in the human adult ovary. *Nat Commun* 2019;**10**:3164.

Fan X, Moustakas I, Bialecka M, Del Valle JS, Overeem AW, Louwe LA, Pilgram GSK, Van Der Westerlaken LAJ, Mei H, Chuva De Sousa Lopes SM. Single-Cell Transcriptomics Analysis of Human Small Antral Follicles. *IJMS* 2021;**22**:11955.

Fowler PA, Flannigan S, Mathers A, Gillanders K, Lea RG, Wood MJ, Maheshwari A, Bhattacharya S, Collie-Duguid ESR, Baker PJ, *et al.* Gene expression analysis of human fetal ovarian primordial follicle formation. *J Clin Endocrinol Metab* 2009;**94**:1427–1435.

Galatidou S, Petelski AA, Pujol A, Lattes K, Latorraca LB, Fair T, Popovic M, Vassena R, Slavov N, Barragán M. Single-cell proteomics reveals decreased abundance of proteostasis and meiosis proteins in advanced maternal age oocytes. *Mol Hum Reprod* 2024;**30**:gaae023.

Garcia-Alonso L, Lorenzi V, Mazzeo CI, Alves-Lopes JP, Roberts K, Sancho-Serra C, Engelbert J, Marečková M, Gruhn WH, Botting RA, *et al.* Single-cell roadmap of human gonadal development. *Nature* 2022;**607**:540–547.

Ghevaria H, SenGupta S, Naja R, Odia R, Exeter H, Serhal P, Gonzalez XV, Sun X, Delhanty J. Next Generation Sequencing Detects Premeiotic Errors in Human Oocytes. *IJMS* 2022;**23**:665.

Gkountela S, Zhang KX, Shafiq TA, Liao W-W, Hargan-Calvopiña J, Chen P-Y, Clark AT. DNA Demethylation Dynamics in the Human Prenatal Germline. *Cell* 2015;**161**:1425–1436.

Gokyer D, Akinboro S, Zhou LT, Kleinhans A, Laronda MM, Duncan FE, Riley JK, Goldman KN, Babayev E. The oocyte microenvironment is altered in adolescents compared to oocyte donors. *Hum Reprod Open* 2024;**2024**:hoae047.

Grøndahl ML, Yding Andersen C, Bogstad J, Nielsen FC, Meinertz H, Borup R. Gene expression profiles of single human mature oocytes in relation to age. *Human Reproduction* 2010;**25**:957–968.

Gu Y, Zhang X, Wang R, Wei Y, Peng H, Wang K, Li H, Ji Y. Metabolomic profiling of exosomes reveals age-related changes in ovarian follicular fluid. *Eur J Med Res* 2024;**29**:4.

Guahmich NL, Man L, Wang J, Arazi L, Kallinos E, Topper-Kroog A, Grullon G, Zhang K, Stewart J, Schatz-Siemers N, *et al.* Human theca arises from ovarian stroma and is comprised of three discrete subtypes. *Commun Biol* 2023;**6**:7.

Guo F, Yan L, Guo H, Li L, Hu B, Zhao Y, Yong J, Hu Y, Wang X, Wei Y, *et al.* The Transcriptome and DNA Methylome Landscapes of Human Primordial Germ Cells. *Cell* 2015;**161**:1437–1452.

Guo Y, Cai L, Liu X, Ma L, Zhang H, Wang B, Qi Y, Liu J, Diao F, Sha J, *et al.* Single-Cell Quantitative Proteomic Analysis of Human Oocyte Maturation Revealed High Heterogeneity in In Vitro-Matured Oocytes. *Mol Cell Proteomics* 2022;**21**:100267.

Hou Y, Fan W, Yan L, Li R, Lian Y, Huang J, Li J, Xu L, Tang F, Xie XS, *et al.* Genome Analyses of Single Human Oocytes. *Cell* 2013;**155**:1492–1506.

Hu W, Zeng H, Shi Y, Zhou C, Huang J, Jia L, Xu S, Feng X, Zeng Y, Xiong T, *et al.* Single-cell transcriptome and translatome dual-omics reveals potential mechanisms of human oocyte maturation. *Nat Commun* 2022;**13**:5114.

Huang J, Chen P, Jia L, Li T, Yang X, Liang Q, Zeng Y, Liu J, Wu T, Hu W, *et al.* Multi-Omics Analysis Reveals Translational Landscapes and Regulations in Mouse and Human Oocyte Aging. *Adv Sci (Weinh)* 2023;**10**:e2301538.

Hurwitz JM, Jindal S, Greenseid K, Berger D, Brooks A, Santoro N, Pal L. Reproductive aging is associated with altered gene expression in human luteinized granulosa cells. *Reprod Sci* 2010;**17**:56–67.

Jain N, Li JL, Tong L, Jasmine F, Kibriya MG, Demanelis K, Oliva M, Chen LS, Pierce BL. DNA methylation correlates of chronological age in diverse human tissue types. *Epigenetics & Chromatin* 2024;**17**:25.

Jiang Z-X, Wang Y-N, Li Z-Y, Dai Z-H, He Y, Chu K, Gu J-Y, Ji Y-X, Sun N-X, Yang F, *et al.* The m6A mRNA demethylase FTO in granulosa cells retards FOS-dependent ovarian aging. *Cell Death Dis* 2021;**12**:744.

Jin C, Wang X, Yang J, Kim S, Hudgins AD, Gamliel A, Pei M, Contreras D, Devos M, Guo Q, *et al.* Molecular and genetic insights into human ovarian aging from single-nuclei multi-omics analyses. *Nat Aging* 2025;**5**:275–290.

Jones ASK, Hannum DF, Machlin JH, Tan A, Ma Q, Ulrich ND, Shen Y-C, Ciarelli M, Padmanabhan V, Marsh EE, *et al.* Cellular atlas of the human ovary using morphologically guided spatial transcriptomics and single-cell sequencing. *Sci Adv* 2024;**10**:eadm7506.

Kavarthapu R, Lou H, Pham T, Do H, Soliman ME, Badger T, Balasubramanian R, Huyhn V, De La Luz Sierra M, Yano Maher JC, *et al.* Single-nucleus and spatial transcriptomics of paediatric ovary: Molecular insights into the dysregulated signalling pathways underlying premature ovarian insufficiency in classic galactosemia. *Clin Transl Med* 2024;**14**:e70043.

Kristensen SG, Ebbesen P, Andersen CY. Transcriptional profiling of five isolated size-matched stages of human preantral follicles. *Molecular and Cellular Endocrinology* 2015;**401**:189–201.

Lecluze E, Rolland AD, Filis P, Evrard B, Leverrier-Penna S, Maamar MB, Coiffec I, Lavoué V, Fowler PA, Mazaud-Guittot S, *et al.* Dynamics of the transcriptional landscape during human fetal testis and ovary development. *Human Reproduction* 2020;**35**:1099–1119.

Lengyel E, Li Y, Weigert M, Zhu L, Eckart H, Javellana M, Ackroyd S, Xiao J, Olalekan S, Glass D, *et al.* A molecular atlas of the human postmenopausal fallopian tube and ovary from single-cell RNA and ATAC sequencing. *Cell Reports* 2022;**41**:111838.

Lewandowska AE, Macur K, Czaplewska P, Liss J, Łukaszuk K, Ołdziej S. Qualitative and Quantitative Analysis of Proteome and Peptidome of Human Follicular Fluid Using Multiple Samples from Single Donor with LC-MS and SWATH Methodology. *J Proteome Res* 2017;**16**:3053–3067.

Li J, Lu M, Zhang P, Hou E, Li T, Liu X, Xu X, Wang Z, Fan Y, Zhen X, *et al.* Aberrant spliceosome expression and altered alternative splicing events correlate with maturation deficiency in human oocytes. *Cell Cycle* 2020;**19**:2182–2194.

Li L, Dong J, Yan L, Yong J, Liu X, Hu Y, Fan X, Wu X, Guo H, Wang X, *et al.* Single-Cell RNA-Seq Analysis Maps Development of Human Germline Cells and Gonadal Niche Interactions. *Cell Stem Cell* 2017;**20**:858-873.e4.

Liu Q, Wei F, Wang J, Liu H, Zhang H, Liu M, Liu K, Ye Z. Molecular mechanisms regulating natural menopause in the female ovary: a study based on transcriptomic data. *Front Endocrinol* 2023;**14**:1004245.

Liu Y, Tao W, Wu S, Zhang Y, Nie H, Hou Z, Zhang J, Yang Z, Chen Z-J, Wang J, *et al.* Maternal mRNA deadenylation is defective in in vitro matured mouse and human oocytes. *Nat Commun* 2024;**15**:5550.

Liu Z, Zhou Q, Zan J, Tian J, Zhang Y, Wu F, Zhao H, Peng Q, Liu S, Chen Q, *et al.* Proteomic Analysis of Human Follicular Fluid-Derived Exosomes Reveals That Insufficient Folliculogenesis in Aging Women is Associated With Infertility. *Mol Cell Proteomics* 2025;**24**:100930.

Llonch S, Barragán M, Nieto P, Mallol A, Elosua-Bayes M, Lorden P, Ruiz S, Zambelli F, Heyn H, Vassena R, *et al.* Single human oocyte transcriptome analysis reveals distinct maturation stage-dependent pathways impacted by age. *Aging Cell* 2021;**20**:e13360.

Mamsen LS, Ernst EH, Borup R, Larsen A, Olesen RH, Ernst E, Anderson RA, Kristensen SG, Andersen CY. Temporal expression pattern of genes during the period of sex differentiation in human embryonic gonads. *Sci Rep* 2017;**7**:15961.

Mao R, Cai Z, Wang T, Li Y, Tian S, Li D, Li P. Comparative study of the three-dimensional genomes of granulosa cells in germinal vesicle and metaphase II follicles. *Front Genet* 2024;**15**:1480153.

Markholt S, Grøndahl ML, Ernst EH, Andersen CY, Ernst E, Lykke-Hartmann K. Global gene analysis of oocytes from early stages in human folliculogenesis shows high expression of novel genes in reproduction. *MHR: Basic science of reproductive medicine* 2012;**18**:96–110.

McGlacken-Byrne SM, Del Valle I, Xenakis T, Simcock IC, Suntharalingham JP, Buonocore F, Crespo B, Moreno N, Liptrot D, Niola P, *et al.* Mapping the anatomical and transcriptional landscape of early human fetal ovary development. *Sci Rep* 2025;**15**:15814.

Méar L, Sutantiwanichkul T, Östman J, Damdimopoulou P, Lindskog C. Spatial Proteomics for Further Exploration of Missing Proteins: A Case Study of the Ovary. *J Proteome Res* 2023;**22**:1071–1079.

Molinari E, Bar H, Pyle AM, Patrizio P. Transcriptome analysis of human cumulus cells reveals hypoxia as the main determinant of follicular senescence. *Mol Hum Reprod* 2016;**22**:866–876.

Ntostis P, Iles D, Kokkali G, Vaxevanoglou T, Kanavakis E, Pantou A, Huntriss J, Pantos K, Picton HM. The impact of maternal age on gene expression during the GV to MII transition in euploid human oocytes. *Hum Reprod* 2021;**37**:80–92.

Olsen KW, Castillo-Fernandez J, Zedeler A, Freiesleben NC, Bungum M, Chan AC, Cardona A, Perry JRB, Skouby SO, Borup R, *et al.* A distinctive epigenetic ageing profile in human granulosa cells. *Human Reproduction* 2020;**35**:1332–1345.

Orvieto R, Nayshool O, Cohen L, Yung Y, Aizer A, Saar EG, Dominissini D. Impact of aging on gene expression in human oocytes: a comparative analysis of young and older patients. *Reprod Biol Endocrinol* 2025;**23**:111.

Ouandaogo ZG, Frydman N, Hesters L, Assou S, Haouzi D, Dechaud H, Frydman R, Hamamah S. Differences in transcriptomic profiles of human cumulus cells isolated from oocytes at GV, MI and MII stages after in vivo and in vitro oocyte maturation. *Human Reproduction* 2012;**27**:2438–2447.

Ouni E, Nedbal V, Da Pian M, Cao H, Haas KT, Peaucelle A, Van Kerk O, Herinckx G, Marbaix E, Dolmans M-M, *et al.* Proteome-wide and matrisome-specific atlas of the human ovary computes fertility biomarker candidates and open the way for precision oncofertility. *Matrix Biol* 2022;**109**:91–120.

Ouni E, Vertommen D, Chiti MC, Dolmans M-M, Amorim CA. A Draft Map of the Human Ovarian Proteome for Tissue Engineering and Clinical Applications. *Molecular & Cellular Proteomics* 2019;**18**:S159–S173.

Pei W, Cao X, Zhang P, Fu L, Zhou P, Liu Y, Fan Y, Xu F, Cao C, Yu Y. Spatiotemporal Transcriptome Analysis Reveals Activation of the AP1 Pathway in the Ovarian Microenvironment during the Transition from Premenopause to Postmenopause. *Aging Dis* 2024;**15**:445–448.

Pietroforte S, Barragan Monasterio M, Ferrer-Vaquer A, Irimia M, Ibáñez E, Popovic M, Vassena R, Zambelli F. Specific processing of meiosis-related transcript is linked to final maturation in human oocytes. *Molecular Human Reproduction* 2023;**29**:gaad021.

Pla I, Sanchez A, Pors SE, Pawlowski K, Appelqvist R, Sahlin KB, Poulsen LLC, Marko-Varga G, Andersen CY, Malm J. Proteome of fluid from human ovarian small antral follicles reveals insights in folliculogenesis and oocyte maturation. *Human Reproduction* 2021;**36**:756–770.

Poulsen LC, Bøtkjær JA, Østrup O, Petersen KB, Andersen CY, Grøndahl ML, Englund ALM. Two waves of transcriptomic changes in periovulatory human granulosa cells. *Human Reproduction* 2020;**35**:1230–1245.

Poulsen LLC, Englund ALM, Wissing MLM, Yding Andersen C, Borup R, Grøndahl ML. Human granulosa cells function as innate immune cells executing an inflammatory reaction during ovulation: a microarray analysis. *Molecular and Cellular Endocrinology* 2019;**486**:34–46.

Reyes JM, Silva E, Chitwood JL, Schoolcraft WB, Krisher RL, Ross PJ. Differing molecular response of young and advanced maternal age human oocytes to IVM. *Hum Reprod* 2017;**32**:2199–2208.

Rodríguez-Nuevo A, Torres-Sanchez A, Duran JM, De Guirior C, Martínez-Zamora MA, Böke E. Oocytes maintain ROS-free mitochondrial metabolism by suppressing complex I. *Nature* 2022;**607**:756–761.

Rogers HM, Gad A, Cork GK, Menjivar NG, Schoolcraft WB, Tesfaye D, Yuan Y. Age-related integrative transcriptomic profiling of human granulosa cells reveals mRNA-microRNA regulatory network associated with key ovulation dynamics†. *Biol Reprod* 2025;**112**:916–931.

Rooda I, Hassan J, Hao J, Wagner M, Moussaud-Lamodière E, Jääger K, Otala M, Knuus K, Lindskog C, Papaikonomou K, *et al.* In-depth analysis of transcriptomes in ovarian cortical follicles from children and adults reveals interfollicular heterogeneity. *Nat Commun* 2024;**15**:6989.

Ruth KS, Day FR, Hussain J, Martínez-Marchal A, Aiken CE, Azad A, Thompson DJ, Knoblochova L, Abe H, Tarry-Adkins JL, *et al.* Genetic insights into biological mechanisms governing human ovarian ageing. *Nature* 2021;**596**:393–397.

Shi H, Pan M, Sheng Y, Jia E, Wang Y, Dong J, Tu J, Bai Y, Cai L, Ge Q. Extracellular cell-free RNA profile in human large follicles and small follicles. *Front Cell Dev Biol* 2022;**10**:940336.

Smits MAJ, Janssens GE, Goddijn M, Hamer G, Houtkooper RH, Mastenbroek S. Longevity pathways are associated with human ovarian ageing. *Hum Reprod Open* 2021;**2021**:hoab020.

Smits MAJ, Schomakers BV, van Weeghel M, Wever EJM, Wüst RCI, Dijk F, Janssens GE, Goddijn M, Mastenbroek S, Houtkooper RH, *et al.* Human ovarian aging is characterized by oxidative damage and mitochondrial dysfunction. *Hum Reprod* 2023;**38**:2208–2220.

Taelman J, Czukiewska SM, Moustakas I, Chang YW, Hillenius S, van der Helm T, van der Meeren LE, Mei H, Fan X, Chuva de Sousa Lopes SM. Characterization of the human fetal gonad and reproductive tract by single-cell transcriptomics. *Dev Cell* 2024;**59**:529-544.e5.

Takeuchi H, Yamamoto M, Fukui M, Inoue A, Maezawa T, Nishioka M, Kondo E, Ikeda T, Matsumoto K, Miyamoto K. Single-cell profiling of transcriptomic changes during *in vitro* maturation of human oocytes. *Reprod Medicine & Biology* 2022;**21**:e12464.

Tong X-H, Xu B, Zhang Y-W, Liu Y-S, Ma C-H. Research resources: comparative microRNA profiles in human corona radiata cells and cumulus oophorus cells detected by next-generation small RNA sequencing. *PLoS One* 2014;**9**:e106706.

Varik I, Saretok KJ, Rosenberg K, Quintero I, Puhka M, Volkova N, Trošin A, Guazzi P, Velthut-Meikas A. Small and Large Extracellular Vesicles From Human Preovulatory Follicular Fluid Display Distinct ncRNA Cargo Profiles and Differential Effects on KGN Granulosa Cells. *J Extracell Vesicles* 2025;**14**:e70119.

Velthut-Meikas A, Simm J, Tuuri T, Tapanainen JS, Metsis M, Salumets A. Research resource: small RNA-seq of human granulosa cells reveals miRNAs in FSHR and aromatase genes. *Mol Endocrinol* 2013;**27**:1128–1141.

Virant-Klun I, Leicht S, Hughes C, Krijgsveld J. Identification of Maturation-Specific Proteins by Single-Cell Proteomics of Human Oocytes. *Molecular & Cellular Proteomics* 2016;**15**:2616–2627.

Wagner M, Yoshihara M, Douagi I, Damdimopoulos A, Panula S, Petropoulos S, Lu H, Pettersson K, Palm K, Katayama S, *et al.* Single-cell analysis of human ovarian cortex identifies distinct cell populations but no oogonial stem cells. *Nat Commun* 2020;**11**:1147.

Wang C, Zhao X, Wu Z, Huang G, Lin R, Chen H, Xu K, Sun K, Zhou H, Shu J. Identification of Differentially Expressed mRNAs and miRNAs and Related Regulatory Networks in Cumulus Oophorus Complexes Associated with Fertilization. *Reprod Sci* 2024;**31**:1408–1419.

Wang R, Liu X, Li L, Yang M, Yong J, Zhai F, Wen L, Yan L, Qiao J, Tang F. Dissecting Human Gonadal Cell Lineage Specification and Sex Determination Using A Single-cell RNA-seq Approach. *Genomics, Proteomics & Bioinformatics* 2022;**20**:223–245.

Williams Z, Morozov P, Mihailovic A, Lin C, Puvvula PK, Juranek S, Rosenwaks Z, Tuschl T. Discovery and Characterization of piRNAs in the Human Fetal Ovary. *Cell Rep* 2015;**13**:854–863.

Wu H, Zhu R, Zheng B, Liao G, Wang F, Ding J, Li H, Li M. Single-Cell Sequencing Reveals an Intrinsic Heterogeneity of the Preovulatory Follicular Microenvironment. *Biomolecules* 2022;**12**:231.

Wu M, Tang W, Chen Y, Xue L, Dai J, Li Y, Zhu X, Wu C, Xiong J, Zhang J, *et al.* Spatiotemporal transcriptomic changes of human ovarian aging and the regulatory role of FOXP1. *Nat Aging* 2024;**4**:527–545.

Wu Y-W, Li S, Zheng W, Li Y-C, Chen L, Zhou Y, Deng Z-Q, Lin G, Fan H-Y, Sha Q-Q. Dynamic mRNA degradome analyses indicate a role of histone H3K4 trimethylation in association with meiosis-coupled mRNA decay in oocyte aging. *Nat Commun* 2022;**13**:3191.

Wyse BA, Fuchs Weizman N, Kadish S, Balakier H, Sangaralingam M, Librach CL. Transcriptomics of cumulus cells – a window into oocyte maturation in humans. *J Ovarian Res* 2020;**13**:93.

Xu B, Zhang Y-W, Zheng S-X, Tong X-H, Liu Y-S. Expression Profile of microRNAs and Their Targeted Pathways in Human Ovaries Detected by Next-Generation Small RNA Sequencing. *DNA Cell Biol* 2016;**35**:226–234.

Xu Y-W, Wang B, Ding C-H, Li T, Gu F, Zhou C. Differentially expressed micoRNAs in human oocytes. *J Assist Reprod Genet* 2011;**28**:559–566.

Yan R, Gu C, You D, Huang Z, Qian J, Yang Q, Cheng X, Zhang L, Wang H, Wang P, *et al.* Decoding dynamic epigenetic landscapes in human oocytes using single-cell multi-omics sequencing. *Cell Stem Cell* 2021;**28**:1641-1656.e7.

Yang Q, Li R, Lyu Q, Hou L, Liu Z, Sun Q, Liu M, Shi H, Xu B, Yin M, *et al.* Single-cell CAS-seq reveals a class of short PIWI-interacting RNAs in human oocytes. *Nat Commun* 2019;**10**:3389.

Yao H, Zhang D, Jin H, Guo Y, Liu Y, Wang S, Li T, Yuan S, Lu G, Sun Y. RNA multi-omics in single cells reveal rhythmical RNA reshaping during human and mouse oocyte maturation. *BMC Biol* 2025;**23**:147.

Ye M, Yang Z-Y, Zhang Y, Xing Y-X, Xie Q-G, Zhou J-H, Wang L, Xie W, Kee K, Chian R-C. Single-cell multiomic analysis of in vivo and in vitro matured human oocytes. *Human Reproduction* 2020;**35**:886–900.

Yerushalmi GM, Salmon-Divon M, Yung Y, Maman E, Kedem A, Ophir L, Elemento O, Coticchio G, Dal Canto M, Mignini Renzinu M, *et al.* Characterization of the human cumulus cell transcriptome during final follicular maturation and ovulation. *Mol Hum Reprod* 2014;**20**:719–735.

Yi J, Hu H, Shi P, Shi S, Zhao J, Xu L, Yang W, Li B, Zhu J, Zou S. Differential analysis of quantitative proteome and acetyl-proteome profiling between premenopausal and postmenopausal ovarian tissues. *Clin Proteomics* 2018;**15**:36.

Yoo SW, Savchev S, Sergott L, Rezai T, Lopez MF, Von Wald T, Eaton JL, Reindollar R, Usheva A. A Large Network of Interconnected Signaling Pathways in Human Ovarian Follicles is Supported by the Gene Expression Activity of the Granulosa Cells. *Reprod Sci* 2011;**18**:476–484.

Yu B, Dong X, Gravina S, Kartal Ö, Schimmel T, Cohen J, Tortoriello D, Zody R, Hawkins RD, Vijg J. Genome-wide, Single-Cell DNA Methylomics Reveals Increased Non-CpG Methylation during Human Oocyte Maturation. *Stem Cell Reports* 2017;**9**:397–407.

Yu B, Doni Jayavelu N, Battle SL, Mar JC, Schimmel T, Cohen J, Hawkins RD. Single-cell analysis of transcriptome and DNA methylome in human oocyte maturation. El-Maarri O (ed). *PLoS ONE* 2020;**15**:e0241698.

Yu B, Russanova VR, Gravina S, Hartley S, Mullikin JC, Ignezweski A, Graham J, Segars JH, DeCherney AH, Howard BH. DNA methylome and transcriptome sequencing in human ovarian granulosa cells links age-related changes in gene expression to gene body methylation and 3′-end GC density. *Oncotarget* 2015;**6**:3627–3643.

Yu Y, Zhu M-J, Wei C-F, Yang J, Song J-Y, Dong L, Xiang S, Zhang L, Qiu Y, Lian F. Age-related differential gene expression in granulosa cells and its effects on fertility using high-throughput transcriptomics. *Syst Biol Reprod Med* 2022;**68**:190–202.

Yuan L, Yin P, Yan H, Zhong X, Ren C, Li K, Chin Heng B, Zhang W, Tong G. Single-cell transcriptome analysis of human oocyte ageing. *J Cellular Molecular Medi* 2021;**25**:6289–6303.

Zhang M, Lu Y, Chen Y, Zhang Y, Xiong B. Insufficiency of melatonin in follicular fluid is a reversible cause for advanced maternal age-related aneuploidy in oocytes. *Redox Biol* 2020;**28**:101327.

Zhang X, Yang J, Yang W, Cui N, Duan T, Li S, Cao J, Bush SJ, Tong G. Discordant effects of maternal age on the human MII oocyte transcriptome. *Mol Hum Reprod* 2025:gaaf038.

Zhang Y-R, Yin Y, Guo S-M, Wang Y-F, Zhao G-N, Ji D-M, Zhou L-Q. The landscape of transcriptional profiles in human oocytes with different chromatin configurations. *J Ovarian Res* 2024;**17**:99.

Zhang Z, Luo L, Fan S, Bai S, Xu B. Single-cell proteomics analysis of human oocytes during GV-to-MI transition. *Hum Reprod* 2025;**40**:1332–1343.

Zhao H, Li T, Zhao Y, Tan T, Liu C, Liu Y, Chang L, Huang N, Li C, Fan Y, *et al.* Single-Cell Transcriptomics of Human Oocytes: Environment-Driven Metabolic Competition and Compensatory Mechanisms During Oocyte Maturation. *Antioxidants & Redox Signaling* 2019;**30**:542–559.

Zhao X, Zhao F, Yan L, Wu J, Fang Y, Wang C, Xin Z, Yang X. Long non-coding ribonucleic acid SNHG18 induced human granulosa cell apoptosis via disruption of glycolysis in ovarian aging. *J Ovarian Res* 2024;**17**:185.

Supplementary Table S3. Summary of the key findings from human ovarian omic studies across the female lifespan

	Ref	Omic(s) profiled	Resoluti on	Total number of samples/ cells	Key findings		
	(Diedrichs *et al.*,2012)				**Profiled samples characteristics**	**Link to maturation**	**Link to development/aging**
		transcriptome	bulk	500 FGC	FGC are enriched in olfactory transduction, steroid hormone biosynthesis, androgen and estrogen metabolism, sensory perception.<br>Meiosis-related genes (*SYCP2, SYCP3, TEX15, STAG3, DMC1)* are detected.		Olfactory receptor-encoding genes might be involved in the FGC development.
	(Williams *et al.,* 2015)	miRNAome	bulk	13 samples	150 piRNA genes detected (2.5% of the total reads), concentrated on chromosomes 9 and 15 (34%).<br>piRNA:miRNA read counts = 1:20. *PIRN71* is the most expressed piRNA gene.		Low abundance of piRNAs could be due to meiotic arrest and the waiting period for post-meiotic differentiation following hormonal stimulation.
	(Del Valle *et al.,* 2017)	transcriptome	bulk	10 samples	Genes enriched in germ cell development, type I interferon signaling.		
	(Wang *et al.,* 2022)	transcriptome	single cell	26,000 cells	6-7W: mitotic FGCs dominate. 13W: RA-responsive, meiotic and oogenesis FGCs emerge (1.3%). By 16W: the FGCs increase to 13.4%.<br>22 W: >50% FGCs in meiosis, rest RA-responsive and oogenetic. Once GC lineage is determined, FGCs in begin meiosis upon exposure to RA. *KRT19+* cells are potentially GREL cells that give rise to SE and GC. Also show potential to develop into TC.		

					FOXL2+ cells mostly closely resemble the adult GC transcriptome.		
	(Li *et al.,* 2017)	transcriptome	single-cell	1,099 cells<br><br>FGC<br>(n = 732)<br><br>Somatic cells<br>(n = 367)	4 types of FGCs are present:<br>mitotic (*POU5F1+/NANOG+*, 5W-26W, population replenishment)<br>RA-signaling (*STRA8+*, from 11W) meiotic prophase (*SYCP1/3+, SPO11+*, PRDM9+, from 14W)<br>oogenesis stage (*ZP3+, OOSP2+,* from 18W).<br>Somatic cells include GC (expressing *WT1* and *FOXL2*), EN at 5W (*PECAM1*, *CDH5*), erythrocytes, macrophages, and early T cells.<br>3 GC subtypes exist:<br>Early (7-10W)<br>Mural (10W-20W)<br>Late (20W-26W)<br>*BMP2* shows high expression in mural and late GCs.		
	(Bothun *et al.,* 2018)	proteome	bulk	5 samples	DNA mismatch repair was the predominant pathway throughout development.		LXR/RXR pathway elevated in the 2nd trimester.
	(Fowler *et al.,* 2009)	transcriptome	bulk	24 samples			*ZAR1*, *ZPBP2, LHX8*, *FIGLA* expression increases at 18-20W.
	(Lecluze *et al.,* 2020)	transcriptome	bulk	24 samples			6 PCW: *NRXN3*, *CNTN1, SET, NPY* in somatic cells; *KIT, POU5F1, NANOG, LIN28A* overexpressed in ovary. 7 PCW: *RPSO1, AMHR2, NANOG* (*RSPO1* in somatic cells)7-9 PCW: *POU5F1, DPPA2/4, LIN28A/B* 12 PCW: *FOXL2, DAZL, PIWIL2*. 13-14 PCW: *MET, DLL4, XIST, VEGFA*. 17 PCW: *MEI1, MEIOB, SPO11, SYCP1–3, FIGLA, NOBOX, SOHLH1*.

	(Mamsen *et al.,* 2017)	transcriptome	bulk	19 samples	Expression of steroidogenic genes was low, irrespective of age.		*FOXL2* expression detected around 6 PCW, exactly at the initiation of ovarian sex differentiation.
	(Taelman *et al.,* 2024)	transcriptome	single-cell	21,049 cells<br><br>1st trimester: 10,284 cells<br>2nd trimester 10,765 cells	Gonadal stromal cells express *PDGFRA, GATA2*		Gonadal niche interstitial cells (6-7 PCW) develop into<br>pre-GC in 2 waves, with distinct molecular signatures between 6-10 PCW and 10-18 PCW.
	(Guo *et al.,* 2015)	transcriptome, methylome	single-cell	scRNA-seq<br><br>FGC<br>(n = 84)<br>Somatic cells<br>(n = 38)<br><br>Methylome<br><br>FGC<br>(n = 4)<br>Somatic cells<br>(n = 1)			4 PCW:<br>cytoskeleton organization genes (*ACTC1, IFITM);* X chromosome reactivated, active demethylation.<br>8 PCW:<br>negative regulation of gene expression (*UNG, LMO4, PLK1)*<br>10 PCW:<br>response to oxidative stress (*SUMG1, DMRT1, NDUFA3);* lowest DNA methylation (~6%)<br>11PCW:<br>start of global remethylation (~9%)<br>17 PCW:<br>meiotic entry (*SYCP1, SYCE3)*;<br>heterogeneous transcriptional profile.
	(Chitiashvili *et al.,* 2022)	transcriptome	single-cell	25,000 cells/5 donors		Upregulation of X-linked gene expression<br>during meiosis may support<br>mature oocyte formation**.**	7-8 PCW:<br>FGC express *XACT,* somatic cells do not.<br>9-10 PCW:<br>FGC begin differentiating into meiotic germ cells, repressing *NANOG.*<br>X/A ratio increases as FGC enter meiosis.<br>Declines after prophase I without *XIST*, marking oocyte-specific X-chromosome regulation.
	(Hourmand *et al.,* 2009)	transcriptome	bulk	17 samples			Meiosis-related *SPO11, SYCP3, STAC3, TEX11, TEX14* elevate after 12 PCW and remain elevated.
	(Gkountela *et al.,* 2015)	transcriptome	bulk	RNA-seq:<br><br>9 samples			9.5 PCW:<br>FGCs become AGCs, expressing *TEX14, FIGLA, ZP3* (follicle formation) and

				Methylome:<br><br>4 samples			*NOBOX, SOHLH1/2, LHX8* (maintenance)<br>PGC and AGC show minimal CpG methylation (16.7% at 16.1 PCW), with AGCs showing reduced methylation in gene bodies and regulatory regions, without affecting gene expression.
	(Garcia-Alonso *et al.,* 2022)	transcriptome<br>spatial transciptome<br>chromatin accessibility	single-cell	scRNA-seq (cells/individual)<br>153,350/28<br>1st trimester:<br>81,885/16<br>2nd trimester:<br>71,465/12<br><br>scATAC-seq (cells/individual)<br>58,684/8<br>1st trimester:<br>17,004/3<br>2nd trimester:<br>41,680/4<br><br>Multiome (cells/individual)<br>33, 062/5<br>1st trimester:<br>12651/1<br>2nd trimester:<br>20,411/4<br><br>Spatial transcriptome<br><br>4 samples (Week 11, 14, 17, 19)			PGCs (*NANOS3+, POU5F1+, NANOG+*) decrease from 100% at 6-7 PCW to 12% at 18-21 PCW. FGCs (*NANOG-, DDX4+, DAZL+*) develop into oogonia by 11 PCW (50% of germ cells until 21 PCW). Pre-oocytes (*FIGLA+*) appear at 14 PCW, followed by primary oocytes (*FIGLA+/GDF9+*) at 16 PCW. PGCs interact with OSE and preGC-IIa cells in outer cortex, supported by KITLG and ECM proteins.<br>Early somatic cells (6 PCW) express *GATA4, LHX9.* Gonadal interstitial cells appear at 6 PCW, declining by 9 PCW. ESGCs (6-8 PCW) express *LGR5+, FOXL2+,* and activate RSPO1/WNT4-beta-catenin pathway.<br>PreGC development occurs in 2 waves:<br>1st wave at<br>8 PCW, later restricted to medulla,<br>2nd wave at<br>mid-8 PCW (2 subtypes)<br>17 PCW: follicular GC emerge expressing NOTCH pathway genes.<br>At 17 PCW, primordial follicles form from primary oocytes and GC, using *HES1+, HEY1/2+* signaling and cell adhesion.
	(McGlacken-Byrne *et al.,* 2025)	transcriptome	bulk, single-cell	1 trimester:<br>15 samples<br>10,291 cells<br>2nd trimester:	7.5-8 PCW: neuroactive genes (e.g., *NLRP7, NPY, TAC1)* are highly expressed.		*FOXL2* role in a major ovary developmental pathway confirmed.

				4 samples	9-10 PCW: *CRYM* is expressed in pre-GC clusters, *CYP19A1* in pre-GC and oogonia. Increase in Homeobox family TFs (e.g., *NOBOX, LHX2, IRX3)*, decrease in zf-C2H2 TFs (e.g., *ZNF727, ZNF729)*.<br>11-12 PCW: meiosis starts.<br>15-16 PCW: meiosis peaks. *MAEL* and *TERB* highly expressed in later germ cell clusters.		Meiosis-related genetic networks highly expressed, particularly enriched for DNA repair genes.<br><br>Neuroendocrine programs exist in the developing fetal ovary, primarily expressed in somatic cell populations and potentially relation to ovarian pubertal activation.<br><br>Sympathetic innervation might be important for ovarian development.
	(Ouni *et al.,* 2022)	proteome	bulk	15 samples			<u>Matrisome:</u> Prepubertal ovaries show 13 upregulated proteins for elastic fiber assembly (collagen VI, FBLN5, POSTN) and ECM remodeling (TXN). Basement membrane proteins (COL4, NID1, ANXA7) exceed adult levels. Anti-apoptotic (HRNR) and pro-apoptotic (OGN, TGFBI) proteins are elevated, regulating follicular atresia. Collagens show high hydroxylation. FBLN5, POSTN, and TNXB drive elastic remodeling for ovary development.<br><br><u>Intracellular proteome:</u><br>182 upregulated proteins are enriched in mitochondrial damage (PARP1, MIF, VDAC1, SLC25A4 for cytochrome C release; PPID, PHB2 for autophagy; UTRN, RALA, CS for fission), immune chemotaxis (S100A8/A9, PTGDR for neutrophils; PPIB, MIF for macrophages), and RNA clearing (DDX1, RPS3, RPL30, PTBP1, RPS14 for splicing; SRSF1/2, GPX1 for mRNA catabolism).
	(Rooda *et al.,* 2024)	transcriptome, miRNAome	mini-bulk	Transcriptome: 109 follicles<br><br>miRNAome:	Follicles separated into 2 distinct groups (unaffected by age of the donor):	Largest transcriptomic changes:	<u>Prepubertal miRNAome:</u><br>Enriched for cytokine-related pathways and PI3K, HIF1, Jak-STAT, FoxO

				113 follicles	Group 1 (64%): all primordial + some growing follicles. Oocyte markers (*DDX4, DAZL, FIGLA)* upregulated. Oocyte present. Meiosis-, cell cycle-, steroidogenesis-related processes upregulated.<br>Group 2 (36%): only growing follicles. GC markers (*AMH, FOXL2*) upregulated. Oocyte present. ECM-, communication, development-related processes upregulated. Stronger communication signals, compared to Group 1. Top signals: *MK, SEMA-3*. Might have supporting role for Group 1 follicles, playing role in local paracrine regulations.<br><br>*HIF3A* expression is higher in child follicles at RNA and protein levels, suggesting varying oxygen sensitivity that may impact fertility preservation.<br><br>Chemotherapy-treated patients (especially higher doses) showed interferon signaling pathway enrichment and *IFI44L* upregulation, confirmed by immunostaining.	primordial-to-primary transition<br><br>Largest miRNAome changes:<br><br>primordial vs intermediate; primordial vs primary (prepubertal)<br><br>primordial vs secondary (adult)	signaling. Overall greater level of predicted miRNA regulatory involvement.<br><br>Adult miRNAome:<br>Enriched for cell cycle-, steroid-, fatty acid metabolism-related pathways.<br><br>Child-adult differences included *ACTA2, SPARCL1* (early TC markers). Lack of differences in GC-derived growth factors suggests surrounding stroma as the source of variation.
	(Kavarthapu *et al.,* 2024)	transcriptome, spatial transcriptome	single-cell	snRNA-seq 81,603 nuclei/10 patients<br><br>Spatial transcriptome:<br>6 samples/6 patients	Most cells are stromal cells. Least abundant cell types = oocytes and immune cells.<br><br>Stromal cell subclusters: SC1 (*FGF10, ANO4, CADM2, ITGA8*): fibroblast-like; SC2 (*COL1A1, COL3A, GLI2, COL5A1*): ECM-producing; SC3 (*CYP17A1,*		

					LHCGR, CYP11A1, HSD17B6): steroidogenic; SC4 (*ENPEP, ADGRG4, FBLN1, DPT*): perifollicular.		
	(Hu *et al.,* 2022)	transcriptome, translatome	mini-bulk, single-cell	10-GV oocytes mini-bulks (n = 20); single GV oocytes (n = 3); 10-MII oocytes mini-bulks (n = 20); single GV oocytes (n = 3); single IVM oocytes (n = 14)		Gene transcription levels generally decreased or remained stable from GV to MII stages, with regulation occurring sequentially from cytoplasm to nucleus.<br><br>*OOSP2* is co-expressed with *BMP15* and *HF100*, suggesting its role in oocyte maturation, with human recombinant OOSP2 accelerating *in vitro* maturation.	The translatome provides a more accurate reflection of changes along the developmental pathway.<br><br>GV and MII oocytes show greater differences in their translational profiles, compared to their transcriptional ones.
	(Yu *et al.,* 2022)	transcriptome	bulk	10 samples (5/group)			Upregulated DEGs in the older group are enriched in the immune system and downregulated in signal transduction.
	(Li *et al.,* 2020)	transcriptome	single-cell	6 oocytes (3/group)		Mitochondrial protein-encoding genes (incl. *COX6B1, COX7C, UQCC2, C14orf2*) downregulated in MD oocytes;<br><br>Pre-mRNA splicing is important for oocyte maturation.	
	(Battaglia *et al.,* 2016)	miRNAome	bulk	12 samples (6/group; 2/patient)			Older reproductive age group:<br>3 miRNAs (let-7b-5p, miR-19a-3p, miR-519d-3p) strongly downregulated.<br>9 miRNAs (let-7e-5p, miR-29a-3p, miR126-3p, miR-136-5p, miR-192-5p,

							miR-203a-3p, miR-371-3p, miR-484, and miR-494-3p) upregulated.
	(Ernst *et al.,* 2018)	transcriptome	bulk	12 samples (4/patient)	GC-specific primordial-to-primary follicle transition genes: *AR, YYI, ZFX, FOXL, CLGN, SLC35G1, FOG2*<br>Primary follicles-specific genes: *FOG2, KITLG, ZFX*	Primordial-to-primary follicle transition: STAT3 pathway upregulated.<br>WNT/Ca2+ pathway downregulated.	
	(Xu *et al.,* 2021)	microRNAome	bulk	3 samples	5 most abundant miRNA:<br><br>hsa-let-7a-5p, hsa-let-7c-5p, hsa-miR-140-3p, hsa-let-7e-5p, hsa-miR-199a-3p		The let-7 family of miRNAs showed the highest levels of miRNA modification and editing.
	(Assou *et al.,* 2013)	microRNAome	bulk	MII oocytes (n = 10)<br>CC (n = 10)	3 most abundant miRNA in MII oocytes:<br>hsa-miR184, hsa-miR10A, has-miR100<br><br>3 most abundant miRNA in CC: hsa-let-7b, hsa-let-7c, hsa-miR21		Abundant miRNA in CC are assosiated with regulation of ECM formation and apoptosis.
	(Al-Edani *et al.,* 2014)	transcriptome	bulk	28 samples			Underexpressed in older CC:<br>genome integrity-related genes (*MSRB3, CHAF1A, POLH*)<br>Overexpressed in older CC:<br>glucose metabolism-related genes (*ALG13, GLT8D3*)
	(Tong *et al.,* 2014)	miRNAome	bulk	U	let-7 miRNA family is the most abundant		
	(Velthut-Meikas *et al.,* 2013)	transcriptome, miRNAome	bulk	Transcriptome:<br><br>MGC samples (n = 3)<br>CC samples (n = 3)<br><br>miRNAome:<br>MGC samples (n = 6)<br>CC samples (n = 6)	Mature miRNAs: MGC (n=936), CGC (n=883). *hsa-miR-21-5p* most abundant in both cell types. *hsa-let-7f-5p* among top 3 in both. Most abundant miRNAs highly similar between types.<br>Top KEGG pathways enriched for miRNA targets: MGC (ECM-receptor interaction, TGF-b signaling, endocytosis); CGC (glycosaminoglycan		

					biosynthesis-heparan sulphate, lysine degradation, TGF-b signaling). ESR-1 is predicted as miRNA target in CGC.		
	(Reys *et al.,* 2017)	transcriptome	single-cell	GV oocytes (n = 10; 1/patient)<br><br>IVM MII oocytes (n = 10; 1/patient)			Potential markers of oocyte quality (*CDK1, NLRP5, PRDX1*) were decreased in older MII oocytes relative to younger ones.
	(Yan *et al.,* 2021)	transcriptome, methylome, chromatin accessibility	single-cell	DNA<br><br>Growing oocytes (n = 86)<br>Fully grown oocytes (n = 81)<br>MI oocytes (n = 155)<br>MII oocytes (n = 90)<br>GC (n = 93)<br>Stroma cells (n = 374)<br>Immune cells (n = 20)<br><br>RNA<br><br>MII oocytes (n = 42)	Immune cells have the highest methylation level (0.8), followed by GC and stroma (~0.75).<br><br>Cell-type-specific TFs, related to NDRs:<br><br>GC: *NR5A2*<br>Stromal cells: *NFE2L2, FOSL1, ELF3, EHF*<br>Immune cells: *SPI1, MAFB*	Genome-wide GCH methylation level near the TSSs peaks in growing oocytes and drops in mature ones. Early growth stage shows increased accessibility of *de novo* methylated gene promoters. NDRs decrease as oocytes mature.	
	(Xu *et al.,* 2011)	miRNAome	bulk	GV oocytes (n = 60)<br><br>MII oocytes (n = 60)		Upregulated hsa-miR-193a-5p, hsa-miR-297, hsa-miR-625, hsa-miR-602<br>Downregulated:	

						hsa-miR-888*, hsa-miR-212, hsa-miR-662, hsa-miR-299-5p	
	(Yerushalmi *et al.,* 2014)	transcriptome	bulk	Expanded CC from MII oocytes (n = 9/3) Compact CC from GV oocytes (n = 7/2)	Most abundant genes in compact CCs: *CYP19A1, SERPINE2, VCAN, MT-CYB, MT-ND4* Most abundant genes in expanded CCs: *DHCR24, MT-CYB, MT-ND4, FN1, TIMP1*		
	(Guahmich *et al.,* 2023)	transcriptome, chromatin accessibility	single-cell	scRNA-seq<br><br>48,147 cells post-QC<br><br>scATAC-seq<br><br>5,024 cells post-QC	6 major cell populations at the antral follicle stage: EN, SMC, GC, OE, hematopoietic cells, stroma/TC (49.2% of total cell number) The interplay between Hh signaling and TCF21/NR5A1 in the specification of different TC.		Reciprocal role of TCF21 and NR5A1 at the initiation and terminus of TC differentiation.
	(Andrei *et al.,* 2019)	miRNAome	bulk	6 samples (2/individual)	Majority of miRNAs are shared between CC and mural GC. The highest expression levels: miR-21-5p, let-7a-5p, let-7f-5p.		
	(Jiang *et al.,* 2021)	transcriptome, mRNA methylome	bulk	U			m6A levels increase in aged ovaries GCs.<br>Expression of demethylase FTO is downregulated in the older reproductive age group (protein expression likewise).<br>FTO regulates FOS expression via m6A-related<br>pathway.
	(Dong *et al.,* 2019)	transcriptome	single-cell	~700 cells (exact number=U)			CD24 activates EGFR-ERK1/2 pathway and increases<br>expression of prostaglandin synthases and transporters, involved in ovulation.
	(Wu *et al.,* 2022)	transcriptome, mRNA degradome	single-cell	GV oocytes (n = 12; 3/group)<br><br>MII oocytes (n = 12; 3/group)			MII oocyte mRNA levels decline after age 35. CXXC1-maintained H3K4me3, associated with the mRNA decay competence, sets up a timer for oocyte aging.

	(Hurwitz et al., 2010)	transcriptome	bulk	6 samples (3/group)			Consistent upregulation of *GSTT1, INSL3* and upregulation of *IL-1b, IL-1R2, IL-6R* in the GC from older reproductive age patients.
	(Caponnetto *et al.,* 2022)	transcriptome	bulk	38 samples			lncRNA downregulated in the advanced reproductive age women.
	(Smits *et al.,* 2021)	transcriptome	bulk	38 samples			Anti-longevity genes (expression increases with age): *SHC1, PROP1, EIF5A2, EIF3H, EIF4E* Anti-longevity genes (expression decreases with age): *XRCC5, RICTOR,* cell cycle checkpoint genes
	(Huang *et al.,* 2023)	transcriptome, translatome, mRNA methylome	single-cell	6 oocytes (3/group)	Correlation between transcription and translation: -0.0413 (Pearson's)		Translatomics shows clearer separation between young and aged oocytes. Downregulated genes in aged human oocytes are mainly low TE genes.TE of DNA demethylation genes are significantly decreased in aged oocytes.
	(Ruth *et al.,* 2021)	transcriptome	single-cell	22 oocytes			*POLG* and *TP63* mostly expressed during follicular stages for DNA damage-induced apoptosis.
	(Chen *et al.,* 2022)	transcriptome	bulk	9 samples			Downregulation of hsa-circ-0031584 might be associated with ovarian aging.
	(Guo *et al.,* 2022)	proteome	single-cell	GV oocytes (n = 12/6) MII oocytes (n = 12/8) IVM MII oocytes (n = 12/2)	Correlation between RNA and protein expression = 0.196.	mRNA and protein changes are inconsistent during maturation.	
	(Battaglia *et al.,* 2017)	miRNAome	bulk	MII oocyte samples (n = 6) FF samples (n = 6)	let-7b-5p, miR-15b-5p, miR-24-3p are downregulated in MII oocytes compared to FF.		

	(Papler *et al.,* 2015)	transcriptome	bulk	6 samples	GC are enriched in immune and inflammatory responses.<br>CC are enriched in protein binding and cell adhesion.		
	(Diez-Fraile *et al.,* 2014)	transcriptome	bulk	6 samples	4 age-related miRNAs in FF: hsa-mir-21–5p (only in young), hsa-mir-134 (increased in old), hsa-mir-190b, hsa-mir-99b-3p (old group only).		Alteration in the expression of genes regulated by mir-190b could contribute to abnormal glucose metabolism in the follicles of older women.
	(Fan *et al.,* 2021)	transcriptome	single-cell	Oocytes after QC<br>(n = 9)<br><br>Somatic cells<br>(n = 113)	3 antral follicle types, identified by GC transcriptome profiling. Atretic follicles GCs highly express myeloid-leukocyte genes (*FCER1G, CD53, AIF1, CX3CL1*) and potentially convey signals for immune cells filtration.		
	(Wagner *et al.,* 2020)	transcriptome	single-cell	25,131 cells (24,329 cells post-QC)	83% of cells = stromal cells (shared expression of mesodermal lineage markers (e.g., *PDGFRA, DCN*) and ECM proteins (e.g., *COL1A1, COL6A1*), followed by PE (~10%), EN(~5%), GC(~1.2%), immune cells (~0.4%), oocytes (~0.2%).<br>*DDX4+* cluster identified; most of its cells = PE cells (top 25 genes include perivascular markers *MCAM, TAGLN, ACTA2*), do not express germ cell markers (except for *IFITM3*, also expressed in ovarian cortex)		
	(Barone *et al.,* 2020)	genome, transcriptome	DNA: single cell<br><br>RNA:<br><br>bulk	RNA:<br><br>40 samples<br><br>DNA:<br>U			Older reproductive age group: *REEP4* (cell division) downregulation, reduced expression of choromosome segregation genes.18% estimated aneuploidy rate.

	(Fan *et al.,* 2019)	transcriptome	single-cell	56,206 cells/31 samples<br><br>(20,676 cells post-QC)	5 major cell types:<br>GC, EN, SMC, stroma and TC, immune cells.<br>TC from small and bigger antral follicles do not overlap (smaller still have progenitor theca cells, later they branch off to interna and externa theca cells).<br>GC of bigger antral follicles form 2 clusters.<br>GC of atretic follicles show lower levels of *CJA1* and *CDH2*.	Innate immune response during follicular remodeling	
	(Ghevaria *et al.,* 2022)	genome	single-cell	GV oocytes<br>(n = 73)<br>MI oocytes<br>(n = 35)<br>MI oocytes<br>(n = 4)<br>MII oocytes+1st polar body<br>(n = 28)	21.4% showed PM errors.<br>PM aneuploidy greatly increases the risk of an aneuploid mature oocyte.		
	(Yu *et al.,* 2015)	transcriptome, methylome	bulk	RNA:<br>12 samples<br><br>Methylome:<br>8 samples			A genome-wide age-related drift toward more extreme levels of methylation in GC.
	(Chen *et al.,* 2022)	transcriptome	bulk	180 samples			CD4+ cells increase with age; B-cells do not change significantly at ages 20-49 but rise significantly after ages 50-59.
	(Grøndahl, *et al.,* 2010)	transcriptome	bulk	15 samples (10/younger group;<br>5/older group)	Most of MII oocyte genes enriched in cellular metabolic processes.		
	(Ouni *et al.,* 2019)	proteome	bulk	6 samples	Top 10 proteins across lifespan include collagens (most abundant), phosphate dehydrogenase, fibrillin, annexin.<br>Dominant collagen type is collagen VI.		Folliculogenesis stage-specific expression of serpins seems to participate in the growth and atresia of follicles, as it may control ECM remodeling.

	(Zhang *et al.,* 2018)	transcriptome	GC:<br><br>mini-bulk<br><br>Oocytes:<br><br>single-cell	Oocytes<br>(n = 83)<br><br>GC<br>(92 samples;<br>10 cells/follicle/<br>mini-bulk)	Stage-specific TF expression in oocytes:<br><br>Primary: *GTF2I, CSDE1, SOHLH2, SMARCE1, TUB, HBP1, SOX30, HIF1A*<br>Secondary: *KLF2, YBX2, FOXO6, SOX13, ETV5, TEAD2, OTX2*<br>Antral: *PINX1, PBX1, MTF1, SOX15, UBTF, SOX13, POU2F1*<br>Preovulatory: *ATF2, EOMES*<br><br>Stage-specific TF expression in granulosa cells:<br>Primoridal → primary: *CREB1, NFKB1, MEF2A, PIAS1, FOSL2, KLF13, PRDM4*<br>Primary → secondary: *KSCAN3, LMX1B, FOXK1, SIX4*<br>Antral: *MBD1, FIZ1, GABPA, TGIF2, PIAS3, E4F1, IRF3*<br>Preovulatory: *MEIS3, PRDM15, VTN.*		Oocytes showed distinct clustering patterns.<br><br>GC exhibited some overlap across different stages of follicular development.<br><br>GC separate into 5 groups according to the follicular stage.
	(Shi *et al.,* 2022)	transcriptome	bulk	U	Most FF mRNAs were complete fragments.<br>FF cfRNAs from all tissue-specific genes detected.		
	(Wu *et al.,* 2022)	transcriptome	single-cell	15,865 cells<br>(14,592<br>post-QC)	9 cluster of GC in the preovulatory follicles.<br>Macrophages are dominant cell type among immune cells.		
	(Méar *et al.,* 2022)	transciptome, proteome	single-cell,<br>bulk	Transciptome<br><br>42,509 integrated cells<br><br>Proteome:<br>U	*FAM110D* and *TRIM73* showed highest expression in GC, with *FAM110D* also being expressed in EN.		

	(Liu *et al.*, 2023)	transcriptome	bulk, single-cell	112 bulk samples<br><br>*N* of single cells U	Main genes, involved in follicular cell differentiation:<br>*RBP1, TMSB10, SERPINE2, TMSB4X, IFI27.*		Immunoglobin-related genes (*IGLV-51, IGHG1, IGHV4-34, IGLV9-48, IGHV7-4-1*) are significantly elevated in 50-59 age group. Among enriched pathways are antigen processing and presentation and regulation of autophagy.
	(Olsen *et al.*, 2020)	methylome	bulk	63 samples<br>(59 post-QC)			Epimutations increase exponentially with advancing age. Age-associated DMRs found in *VTRNA21,AMH,PGF,GHSR,GHR* (genes involved in folliculogenesis)
	(Ye *et al.*, 2020)	transcriptome, methylome, chromosome ploidy	single-cell	MII oocytes<br>(n = 8)<br><br>IVM oocytes<br>(n = 7)	The average CNV = 1.96; high heterogeneity in the pattern of CNV in the oocytes.	*In vivo* matured oocytes carried more mCHG and mCHH sites across the genes.	
	(Kristensen*et al.,* 2015)	transcriptome	bulk	109 follicles/17 pools/8 patients	Most expressed genes across all stages = *ZP2*.		
	(Yu et al., 2020)	transciptome, methylome	bulk	Methylome:<br>GV oocytes<br>(n = 12)<br>MI oocytes<br>(n = 10)<br>MII oocytes<br>(n = 10)<br><br>scRNA-seq:<br>GV oocytes<br>(n = 7)<br>MI oocytes<br>(n = 7)<br>MII oocytes<br>(n = 7)	GV and MI oocytes are almost indistinguishable<br>(no DEGs between them)<br><br>A weak positive correlation between gene body methylation and gene expression for all cytosine contexts.	DMRs were mostly hypermethylated in mature stages compared to immature ones, with hypermethylated DMRs in MII oocytes correlating with reduced gene expression in the MII/MI	
	(Ernst *et al.*, 2017)	transcriptome	single-cell	6 oocytes<br>(3/group)			Primordial follicle oocytes show ERK/MAPK signaling enrichment, while primary follicle oocytes show HIPPO

							Signalling and ERK5 signaling enrichment.
	(Yoo *et al.,* 2011)	transcriptome, proteome	bulk	U	Secretion of GPX and Sepp by GC is identified.		
	(Zhao *et al.,* 2019)	transcriptome	single-cell	6 oocytes (3/group)		Calcium signaling pathway in IVM oocytes was potentially dysfunctional. IVM and *in vivo* MII oocytes show distinct expression patterns of calcium signaling genes, validated by protein analysis.	
	(Barragán, *et al.,* 2017)	transciptome	bulk	36 samples			ENSG00000221162, hsa-mir-220b, ENSG00000239174, hsa-mir-4262, hsa-mir-1260a increased in older group. piRNAs and lncRNAs mostly increased in young group.
	(Yu *et al.,* 2017)	methylome	single-cell	GV oocytes (n = 10/7 patients)<br><br>MI oocytes (n = 10/4 patients)<br><br>MI oocytes (n = 10/4 patients)		CpG methylation stays stable from GV to MII stage, while CHG and CHH methylation increases with oocyte maturity. CpG methylation initiates in early oocyte growth, with gradual non-CpG methylation following. DMRs decline from 4,377 to 2,826 between GV-MI and MI-MII.	
	(Wyse *et al.,* 2020)	transcriptome	bulk	CC from GV oocytes samples (n = 11)		ECM remodeling differed between maturity cohorts.	

				CC from MII oocytes samples (n = 11)		*PDE3A* upregulation in MII-CC may affect oocyte cytoplasmic maturation.	
	(Takeuchi *et al.,* 2022)	transcriptome	single-cell	IVM-normal:<br>GV oocytes (n = 4)<br>MI oocytes (n = 3)<br>MII oocytes (n = 3)<br><br>IVM-delayed:<br>GV oocytes (n = 3)<br>MI oocytes (n = 3)<br>MII oocytes (n = 3)	MII-upregulated genes showed enrichment in microtubule and cytoskeleton processes.<br><br>*APOB* and *TNFRSF10C* were identified as potential markers for high quality MII oocytes.	The number of expressed genes decreased during oocyte maturation.<br><br>Tubulin genes (*TUBA1A, TUBA3E, TUBA3D*) increased during IVM.	
	(Llonch *et al.,* 2021)	transcriptome	single-cell	GV oocytes (n = 40; 35 post-QC)<br>IVM MI oocytes (n = 32; 31 post-QC)	Oocyte maturation stage emerged as the main driver of transcriptomic variability.<br><br>GV oocytes do not separate based on age.	*BNC1* and *SON* are potential regulators in the IVM MII oocytes.	
	(Hou *et al.,* 2013)	genome	single cell	183 cells	Oocytes are prone to chromosome segregation errors, with numerical abnormalities predominating and most frequently affecting chromosomes 16 and 22.		
	(Ouandaogo *et al.,* 2011)	transcriptome	bulk	24 samples	Top 3 DEGS in CC of GV oocytes:<br>*CBX3, ANKRD57, RAll4*<br>Top 3 DEGS in CC of MI oocytes:<br>*YTHDF2, EEF1A1, SLC38A2*<br>Top 3 DEGS in CC of MII oocytes:<br>*C14orf4, CHCHD1, C5orf15*		

	(Molinari *et al.,* 2016)	transcriptome	bulk	20 samples (10/group)			Hypoxia-related genes increase with age (linked to CC senescence), *ADAMTS14* decreases (ECM turnover), *NOS* increases (inflammation)
	(Markholt et al., 2012)	transcriptome	bulk	3 samples	Most abundantly expressed genes in follicles: *TMEFF2, DC48, OPHN1*		
	(Pietroforte *et al.,* 2023)	transcriptome	single-cell	GV oocytes (n = 10)<br>FTM GV oocytes (n = 10)<br>MII oocytes (n = 10)<br>IVM MII oocytes (n = 10)		Pre-mRNA processing of specific transcripts is linked to successfully completing meiosis I and transitioning through MII.	
	(Yuan *et al.,* 2021)	transcriptome	single-cell	12 oocytes (6/group)			Most of aging-related genes were enriched in ubiquitination and DNA damage response. *UBE2C* might be important for oocyte aging.
	(Virant-Klun *et al.,* 2016)	proteome	single-cell	Immature oocytes (n = 100)<br><br>MII oocytes (n = 100)	Key GV stage protein: TDRKH (for piRNA biogenesis in germ cells)<br><br>Key MII stage- protein: Wee2 (controls meiotic arrest in GV oocytes and metaphase II exit).	GV and MII oocytes are largely resting cells with proteome, focused on homeostasisand secretory interactions.	
	(Pla *et al.,* 2021)	proteome	bulk	36 samples		FF proteomes, surrounding mature and immature oocytes, cluster separately. IGF1 and MDR are upregulated in the FF of MII-capable oocytes.	
	(Cadenas *et al.,* 2023)	transcriptome, proteome	bulk	U		*AREG* and *EREG* strongly upregulate during the	

						first 17h of follicle maturation induction, BTC shows minimal increase. Only AREG detected in the proteomic data.<br>FF-MAS accumulates early in follicle maturation, likely promoting meiosis.	
	(Yang *et al.,* 2019)	miRNAome	single-cell	U	piRNA characteristics: high sequence diversity, 1U/10A sequence preference, and 10-nt 5-end complementary overlap<br><br>A unique category of small RNAs: ~20nt long, makes up > 70% of total small RNAs in oocytes, but is absent in CC and cells from other tissues.<br>Some of highly expressed os-piRNA clusters tend to partially overlap with maternally imprinted genes like *KCNK9*.		
	(Ntosis *et al.,* 2021)	transcriptome	single-cell	GV oocytes (n = 10; 5/group)<br>MII oocytes (n = 11; 5/youngergroup; 6/older group)			Lower number transcripts are significantly differentially expressed during younger GV/MII transition compared to older. Could point to more dysregulated expression in older MII oocytes.
	(Smits *et al.,* 2023)	metabolome, lipidome	bulk	Oocytes (n = 150; 10 oocytes/ group in triplicate)			DNA oxidation shows no age-related increase, while lipid peroxidation and protein oxidation increase in oocytes with age, indicating elevated oxidative stress in older oocytes.
	(Jia *et al.,* 2023)	transcriptome	bulk	U			DEGs in advanced maternal age group showed enrichment in redox processes, mitochondrion function, metal ion and ATP binding, ferroptosis.

	(Wu *et al.*, 2024)	transcriptome, spatial transcriptome	single-cell, bulk	scRNA-seq<br><br>9 samples (3/group)<br><br>92,965 cells<br><br>spatial transcriptome<br><br>15 samples (5/group)	TC & stromal cells are major cell population, divided in 5 subtypes.<br><br>GC divide in 3 subtypes, with subtype 3 prevalent in the older age group.<br><br>Immune cells were mainly distributed in the medullary interstitium.		Lipofuscin accumulation increased with ovarian aging. *CDKN1A* expression was elevated across all cell types in older ovaries, especially in GC, TC & stromal cells.<br><br>*FOXP1, SOX4, FOS* may be key regulators in ovarian aging (based on the single-cell regulatory network inference and clustering analysis). FOXP1 decreased in GC and TC & stroma. Its knockdown in COV434 GC cell line upregulated proteins, associated with cellular senescence and DNA damage. *FOXP1* could inhibit *CDKN1A* transcription through promoter binding.
	(Jones *et al.*, 2024)	transcriptome, spatial transcriptome	single-cell	scRNA-seq:<br><br>21,198 cells/3<br><br>spatial transcriptome 257 regional samples/2 donors	No clear subtypes of stromal cells.<br><br>Expression gradients exist in the ovary: Wnt signaling genes (*DDIT3, DKK1, LEF1, TLR3, ZFP91*) increase outward from the oocyte, while PKA signaling genes (*AKAP12, MIF, RDX, SPHKAP*) increase inward, affecting FSH-induced maturation. A transcriptional gradient exists in the ovarian cortex surface, with increasing expression of hormone signaling genes (*NR4A1, CEBPD, STAR), IGFBP* signaling (*IGFBP2-7*), and ECM remodeling genes (*VIM, COL1A2, TIMPs, MMPs*) from surface inward, varying between donors.		
	(Lewandowska *et al.*, 2017)	proteome	bulk	12 samples/4 patients	Most detected proteins in both fractions localize in the		

					extracellular region, with some in cell parts or organelles; Identified proteins primarily function in binding and catalysis.		
	(Poulsen *et al.,* 2019)	proteome	bulk	25 samples	102/400 identified proteins are immunoglobins.<br>Protein composition changes most drastically immediately after ovulation induction.<br>Immune, inflammatory, ECM-related processed were most enriched.		
	(Rodriguez-Nuevo *et al.,* 2022)	proteome	bulk	4 samples	Early oocytes show low or no mitochondrial ECT activity.<br>54 mitochondrial proteins found in human oocytes comprise 40% of all known mitochondrial proteins, with UPRmt-related proteins showing upregulation.<br>Complex I subunits of OXPHOS machinery are mostly undetectable or present at low levels.		
	(Zhang *et al.,* 2020)	transcriptome	single-cell	6 oocytes (3/group)			Top age-related DEGs: *SYCP2, KIF20B,LRRC16A* (chromosome function-related)
	(Jin *et al.,* 2024)	transcriptome, chromatin accessibility	single-cell	snRNA-seq: 42,568 nuclei/8 patients<br>snATAC-sq: 41,550 nuclei/8 patients	8 major cell types: stromal, EN, GC, SMC, IN, LE, OE cells, TC<br><br>Amount of EN, LE, GC and TC is higher in younger patients.<br><br>Amount of OE cells is higher in aged patients.		Aging-associated DEGs: *RICTOR, IGF1R, MAP3K5, APOE*, enriched in nutrient sensing, cellular senescence, proteostasis, cellular communication and mitochondrial function.<br><br>Aged ovaries exhibit higher senescence markers *CDKN1A (p21)* and *CDKN2A (p16).* Stromal cells with elevated *CDKN1A* show upregulated hypoxia response and HIF-1 signaling. Hypoxia may trigger ovarian cellular senescence via HIF-1 signaling upregulation.

							The mTOR pathway is an ovary-specific aging-prevalent pathway, central to ovarian aging.<br><br>Collagen and FN1 pathway scored high in most cell types in young ovaries but only in epithelial cells in aged ovaries. Aged ovaries show increased cell adhesion pathways across most cell types, alongside the PDGF pathway involved in fibroinflammatory processes.<br><br>All ovarian cell types contribute to ovarian aging.<br><br>CEBPD is potential key regulator of aging-associated gene expression across ovarian cell types.
	(Zhang *et al.,* 2025)	proteome	single-cell	16 oocytes	The average number of proteins in GV oocytes: 1,457<br><br>The average number of proteins in MI oocytes: 1,485	149 DEPs between GV and MI oocytes:<br><br>79 upregulated in MI oocytes (e.g., ATP5MG, COX5B, COX6B1, COX5A, TAGLN2, TIMM8A). Enriched for oxidative phosphorylation (energy consumption during maturation) and thermogenesis. 70 downregulated in MI oocytes (e.g., OOSP2, ATG7, NSF1). Enriched for mitochondria-related processes and phagocytosis.	

	(Dhori *et al.,* 2024)	transcriptome, miRNAome	bulk	transciptome:<br>MGC (3 samples/1 dataset)<br>CC (12 samples/3 datasets)<br>oocyte (6 samples/1 dataset)<br><br>miRNAome:<br>MGC (18 samples/2 datasets)<br>CC (18 samples/2 datasets)	Core set of 236 DEGs, shared between datasets.<br><br>DEMs are coherent in MGC and CC. *miR-129-5p* is the most connected in the miRNA-mRNA network.<br><br>AS events: IR and MES are strong in GC. In pre-ovulatory follicles, MGC vs CC, stimulated vs unstimulated CCs, DAS affect genes involved in “RA receptor binding”, “nuclear hormone receptor binding”, “ubiquitin-mediated proteolysis”. In CC at GV vs CC at MII oocyte DAS affect genes, enriched in “ovarian follicle development”, “response to FSH”, “cadherin binding”. DAS-affected TFs: GATA4, GATA6, NR5A1, NR5A2.	AS might be important in regulating the genes orchestrating maturation and ovulation.<br><br>DAS-affected TFs play role in follicle maturation.	
	(Galatidou *et al.,* 2024)	proteome	single-cell	GV oocytes: (26/2 groups post-QC)<br><br>IVO-MII oocytes: (31/2 groups post-QC)	Young oocytes: 1,368 proteins identified in GV and MII. MII oocytes showed lower abundance of YBOX2, PS14, RS16 (translation/ribosomal functions) and higher abundance of 28 proteins including AURKA, WEE2, BUB1B (cell-cycle regulation/microtubule organization).<br><br>Reproductively aged oocytes: 1,451 proteins identified in GV and MII. MII showed 23 less abundant proteins (YBOX2, RSSA, ZAR1, CAPR2) and 17	Proteasome complex levels decrease from GV to MII stage.	Aging causes subtle proteomic changes that likely contribute to decreased developmental competence in older oocytes.<br><br>No significant differences in protein abundance were detected between Young and Reproductively Aged Groups at the GV stage. The top 3 proteins correlated with age in the GV group were PRS8, 1433E, and HSP7C. In the MII group, IGHG1, C1TM, and CRYM showed the strongest age correlation. Proteins whose abundance decreased with age included key meiosis factors (such as 1433E), proteostasis regulators (UCHL1, HSP7C, CSN3), and co-chaperones. Mitochondria-related

					more abundant ones (ACTL8, PCNA, MP2K1, TBB8B, WEE2).		proteins (ATP5L, MIC60) increased with age.<br><br>Proteasome abundance negatively correlates with aging. Levels of CDK1 (crucial component MPF) decline.<br><br>With age, MII oocytes show decreased TRiC complex abundance, increased tubulin-beta isoforms (TBB5 & TBB8B), and altered telomerase RNA localization.
	(Yao *et al.,* 2025)	transciptome, epitranscriptome	single-cell	13 oocytes	Reduced TE in 3' poly(A) minus transcripts for both GV and MII samples.<br>Morphologically similar oocytes show transcriptional differences.<br>GV oocytes exhibit diverse RNA characteristics.		
	(Orvieto *et al.,* 2025)	transcriptome	single-cell	6 oocytes			Systematic transcriptomic changes with aging.<br><br>Top 3 genes, upregulated in older group: *LINC02087, POMZP3, LINC02749*<br>Top 3 genes, downregulated in older group: *CLEC3A, ARPP21, CITED2*
	(Cadenas *et al.,* 2025)	transcriptome	bulk	22 CC samples post-QC	8,227 DEGs found across groups; major differences between fresh GV and MII-IVM.	Maturation-linked DEGs: *DHCR24, SCN9A, EDIL3, ANOS1, TFPI, RGS5 (↑);*<br>*VIT, CRHBP, PLAT, FAM110C, AMH* (↓)<br><br>EGF-VEGFR2, MAPK, Wnt pathways mostly up; PI3K-AKT down.	

						FSHR expression ↓ from GV to MII-IVM; *LHCGR* ↑<br><br>IGF2 signaling in CCs may enhance oocyte maturation via upregulation of *IGF2R*, *IGFBP2*, *IGFBP4*, *IRS1* and downregulation of *IRS2* in IGF pathway.	
	(Zhang *et al.,* 2024)	transcriptome	single-cell	NSN oocytes (U)<br>SN oocytes (U)	As oocytes matures, chromatin configuration goes from NSN to SN.<br>NSN and SN oocytes are transcriptionally different.	NSN oocytes:<br>*SAT1,* CDK kinases expression is high (high metabolic activity).<br>SN oocytes:<br>Most of genes in SN oocytes ↓. UBE2C ↑. Expression of LINEs is reduced, SINEs are activated. Decreased expression level of *LDHB* along maturation.	
	(Zhao *et al.,* 2024)	transcriptome	bulk	67 GC samples		*NEAT1* increases until secondary stage; *ZFAS1* decreases during follicle development.	*SNHG18* (+ AMH, - female age) downregulated in aging GC. *ENO1* decreased in ovarian aging and correlates with *SNHG18* expression.PKC signaling inhibits apoptosis; downregulation may promote GC apoptosis by disrupting ENO1-mediated glycolysis.

	(Varik *et al.,* 2025)	miRNAome	bulk	6 pooled samples of SEV, LEV from FF/42 patients	SEVs more abundant than LEVs.<br>SEVs: enriched in miRNAs (linked to GC proliferation, differentiation, Ca2+ signaling). Upregulate genes for ECM remodelling and PTK2 signaling (crucial for follicular expansion and oocyte-GC communication); downregulate TGF-β signaling (supporting GC luteinisation via *STAR* expression).<br>LEVs: enriched in piRNAs, may be involved in preserving genomic stability and cellular homeostasis.		
	(Liu *et al.,* 2025)	proteome	bulk	14 samples (7/group)	Most DEPs associated with EVs.	Exosomes promote follicle maturation post-ovulation. ENO1, HSP90B1, and FETUB may facilitate this through exosome delivery to ovary.	Metabolism and immune system proteins significantly lower in aging group. PI3K/AKT pathway most regulated by DEPs. Deficiency in expression of ENO1 (downstream target of PI3K/AKT signaling pathway) in aging group.
	(Zhang *et al.,* 2025)	transcriptome	single-cell	20 oocytes (10/group)	Older oocytes exhibited greater transcriptomic heterogeneity. In general, oocytes age heterogeneously.		1005 upregulated genes (enriched for NER, telomeres, meiotic spindle) and 201 downregulated genes (mitochondria, oxidoreductase complex, filopodial assembly) in older cohort.<br>Age correlations: positive (DERL3, KANK1, RAD51); negative (GLOD4, PSMA1).<br>Poor concordance between single oocyte transcriptomic studies (11% disagreement).
	(Gokyer *et al.,* 2024)	transcriptome	bulk	38 CC samples (19/group)	Cell division/cycle pathway genes were downregulated in adolescents vs. oocyte donors. Adolescents showed enriched pathways for cellular communication, intracellular		Adolescent FFs are more pro-inflammatory, with IL-15 shared with women of advanced reproductive age.

					transport, and organelle organization. This suggests suboptimal oocyte cytoplasmic maturation in adolescents.<br><br>EGF signaling genes (*TMEFF1, ZGPAT, MAPK8IP3, PBK*) showed altered expression in adolescent CCs.		
	(Mao *et al.,* 2024)	transcriptome, 3D genome	single-cell, bulk	Transcriptome: 29 GC from GV and MII oocytes<br><br>3D genome: GC from GV (1), GC from MII (2)		~49% contacts >20kb. GV to MII transition affects 17% of genome; enhances hormone metabolic pathways. *CORIN* linked to oocyte maturation. GV has more TADs than MII, though most remain stable.<br>6 genes show dynamic compartment, TAD and expression changes: *AGPAT4*, *CCN4*, *TCF21*, *SOX4*, *MBD2*, *CDH2*. *KIF26B* also plays an important role in oocyte maturation.	
	(Wang *et al.,* 2024)	transcriptome, miRNAome	bulk	U	miR-877-3p (top 10 hub DEM) targets *GDNF, SIX2, KCJ10, SLC6A7, GDF5*; potentially involved in fertilization. miR-6875-3p upregulation post-fertilization coincides with *GDNF* and *PIK3R2* downregulation.	Calcium pathway activation associates with oocyte maturation and fertilization.	

	(Gu *et al.,* 2024)	metabolome	bulk	U	Identified metabolites: mainly AAs, carbohydrates, organic acids, fatty acids, lipids and nucleotides)		Advanced age group showed higher oleic acids, alpha-lactose, D-maltose, beta-alanine levels (suggesting enhanced glycolysis and decreased fatty acid metabolism) and lower malonic acid. Key affected pathways: pyrimidine, taurine/hypotaurine metabolism, and branched-chain amino acid biosynthesis. Metabolite trends with age: taurine, lactose, glutathione, maltose, oleic acid, melatonin↑; amino adipic acid, alpha-ketoisovaleric acid, uric acid↓. Elevated glutathione in FF exosomes of suggests increased antioxidant production. Increased inosine cytidine taurine in advanced age⇒ possibly related to stress response proteins.
	(Rogers *et al.,* 2025)	transcriptome, miRNAome	bulk	12 GC samples	DEMs target ECM modeling and angiogenesis genes.		Young GC: ↑oxidative stress response genes (*BLC2, HMOX1, BLC2A1*), ↑MT-ND3, MT-ND6, MT-CYB.<br>Aged GC: ↓mitochondrial gene expression; ↓SIGIRR, ↑miR-483-3p (SIGIRR target), ANGPT2.
	(Canicais *et al.,* 2025)	transcriptome	single-cell	19 oocytes	Top genes: GV (*TUBB8, GDF9, MT-ND4*); MI (*TUBB8, H3-3B, TUBB8P7*); MII (*TUBB8, H3-3B, DPPA3*).	DEGs: none between MI-MII; 42 between GV-MI; 1,165 between GV-MII. *TUBB8* was most expressed at all stages with *MT-ND4, MT-CO1, WEE2, OOSP2 and GDF9.*	
	(Liu *et al.,* 2024)	transcriptome	single-cell	17 oocytes		mRNA abundance decreases during oocyte maturation. Maternal mRNA decay is impaired in IVM MII oocytes with higher transcriptional	

						levels compared to IVO MII. *BMP15* is upregulated in IVM MII. Defective deadenylation in IVM MII is linked to impaired cytoplasmic polyadenylation of CCR4-NOT deadenylase and BTG4 adapter mRNAs.	
	(Arbeithuber *et al.,* 2025)	genome	single-cell	80 oocytes	Oocytes showed ~17-24-fold lower mutation frequency than blood/saliva across all ages.<br>No mutation frequency difference between CpG/non-CpG sites in oocytes (unlike other tissues).<br>Higher mutation frequency in D-loop vs. coding region.<br>Fewer disease-associated mutations in oocytes suggests selection against them.		No mutation frequency increase in oocytes from older women.<br>mtDNA mutations may increase with age or mutated oocytes might be eliminated by follicular atresia
	(Boucret et al., 2017)	genome	bulk	53 COC	17 oocyte-specific and 57 CC-specific MT variants identified<br>Fewer function-bearing mitochondrial variants in oocytes vs CC	Variants consistent across maturation stages	No mtDNA mutation accumulation with ovarian aging
	(Jain *et al.,* 2024)	methylome	bulk	157 samples/157 patients			134,986 ovarian age-associated CpG sites (96,210 hypermethylated, 38,776 hypomethylated). Hypermethylated sites not enriched in CpG islands. Hypomethylated sites enriched in enhancers and active transcription regions. Ovary showed most KEGG pathway enrichment across all analyzed tissues, primarily in hormone production/secretion pathways (estrogen, prolactin, aldosterone,

							GnRh).Hypermethylation is more common with increasing age.
	(Yi *et al.*, 2018)	proteome, acetyl-proteome	bulk	6 samples			Postmenopausal upregulated proteins are enriched in extracellular space, downregulated proteins in ECM, immune response, cell adhesion.
	(Lengyel *et al.*, 2021)	transcriptome, chromatin accessbility	single-cell	scRNA-seq<br><br>26,134 cells/6 donors post-QC<br><br>scATAC-seq:<br><br>18,315 cells/3 donors post-QC	7 major cell types: stromal, GC, SMC, PE, EN, LE, immune<br><br>Stroma and GC are predominant cell types, with significant PE is presence. Cell composition remains consistent across donors despite minor variations. Strong expression of several hormone receptors (*AR, PGR, ESR1, PRLR in* SMC; *RARA* in immune and EN).		Immune cells are transcriptionally active, hence organ is not immunologically inert. Most cells of the postmenopausal ovary are ST, but number of EN is also substantial, consistent with the very rich blood supply to the organ. No follicles but organ is not quiescent.
	(Pei *et al.*, 2024)	spatial transcriptome	bulk	10 samples	Expression levels of SMC decreased in premenopausal tissue, suggesting enhanced angiogenesis.<br>The total number and overall strength of intercellular interactions of cell types at spatial levels were reduced, especially between stromal, GC and immune cells.		Gene expression profiles along pseudotime showed enrichment in hormone and stress responses, as well as the AP1 pathway.
	(Ouni *et al.*, 2022)	proteome	bulk	10 samples			Matrisome:<br><br>Proteins involved in ECM production and fibrosis (ANXA5, F2, ITIH4, SERPINA1) are upregulated, with several acting as senescence markers. Elevated CLEC3B expression suggests ongoing inflammation despite ovulation absence. The collagen in menopausal ovaries shows minimal hydroxylation, suggesting

							unstable assembly. ANXA5 leads to p53 upregulation.<br><br>Intracellular proteome:<br><br>Five proteins (PRF1, IAPP, PKM, DBI, GAPDH, SLPI) regulate DNA fragmentation and ROS production, while FLNA, YWHAQ, CSRP1, AFM, and GSTM2 provide cytoprotective functions. TSPO and DAP overexpression inhibits autophagy. The tissue shares 37 proteins with reproductive age ovaries (including ADH5, PEBP1, TALDO1), involved in anti-oxidative responses. AGEs at TBCK, SLITRK4 and HSPA8 trigger inflammatory pathways, supporting the inflammaging hypothesis.
	(Devrukhar *et al.,* 2025)	transcriptome, proteome	single-cell, bulk	transcriptome: doxorubicin-treated:<br>cortex<br>31,909 cells<br>medulla<br>26,626 cells<br>control:<br>cortex<br>19,312 cells<br>medulla<br>18,132 cells			Higher senescence scores in cortex than in medulla at days 6 and 10.<br>Aging (modelled with doxorubicin) altered genes in the cortex, enriched in inflammation-, fibrosis- and immune-related pathways.<br>Compartment specific response to senescence induction:<br>Cortex: activated UPR and immunogenic cell death pathways, which were inactivated in medulla.<br>2 stromal subtypes: 1 (lipid metabolism, cell-matrix adhesion), 2 (matrix fibroblasts).<br>Highest senescence scores: cortex epithelial and stromal-1 cells (top pathway: "integrin signaling"); medulla stromal-1 cells (top pathway: "EIF2 signaling").<br>ECM factors upregulated.<br>Protein alterations: cortex (164 groups: 135↑, 29↓) including DCN, LUM, POSTN; medulla (217 groups: 184↑, 33↓)

							including HSPG2, HSPA9. 120 proteins overlapped.

FGC: fetal germ cells; PGC: primordial germ cells; AGC: advanced germ cells; piRNA: piwi-interacting RNA; PCW: post-conception weeks; W: week; RA: retinoic acid; LXR/RXR: liver X receptor-retinoid X receptor; GREL: gonadal ridge epithelial-like; SE: surface epithelium; OE: ovarian epithelium; GC: granulosa cells; preGC: pre-granulosa cells; TC: theca cells; EN: endothelial cells; SMC: smooth muscle cells; PE: perivascular cells; LE: lymphatic endothelium; FF: follicular fluid; MGC: mural granulosa cells; CC: cumulus cells; COC: cumulus oophorus complexes; X/A ratio: ratio of X-linked to autosomal gene expression; DEG: differentially expressed gene; NDR: nucleosome depleted region; TSS: transcription start site; miRNA: microRNA; lncRNA: long noncoding RNA; m6A: N^6-methyladenosine; TE: translational efficiency; ECM: extracellular matrix; PM: pre-meiotic; cfRNA: cell-free RNA; CNV: copy number variation; DMR: differentially methylated region; CHG, CHH: methylation contexts where H = A, C, T; IVM: *in vitro* maturation; PKA: protein kinase A; FSH: follicle-stimulating hormone; ECT: electron transport chain; UPRmt: unfolded protein response; lncRNA: long non-coding RNA; LINE: long interspersed nuclear element; SINE: short interspersed nuclear element; AGEs: advanced glycation end-products; DEP: differentially expressed proteins; AS: alternative splicing; DAS: differentially alternative splicing; FSH: follicle stimulating hormone; DEM: differentially expressed miRNAs; DEP: differentially expressed protein; IR: intron retention; multiple exon skipping: MES; MPF: matutation-promoting factor; NSN: non-surrounded nucleolus; SN: surrounded nucleolus; SEV: small extracellular vesicles; LEV: large extracellular vesicles; NER: nucleotide excision repair; EGF: epidermal growth factor; U indicates that the parameter is unreported.

References:

Al-Edani T, Assou S, Ferrières A, Bringer Deutsch S, Gala A, Lecellier C-H, Aït-Ahmed O, Hamamah S. Female aging alters expression of human cumulus cells genes that are essential for oocyte quality. *Biomed Res Int* 2014;**2014**:964614.

Andrei D, Nagy RA, van Montfoort A, Tietge U, Terpstra M, Kok K, van den Berg A, Hoek A, Kluiver J, Donker R. Differential miRNA Expression Profiles in Cumulus and Mural Granulosa Cells from Human Pre-ovulatory Follicles. *Microrna* 2019;**8**:61–67.

Arbeithuber B, Anthony K, Higgins B, Oppelt P, Shebl O, Tiemann-Boege I, Chiaromonte F, Ebner T, Makova KD. Allele frequency selection and no age-related increase in human oocyte mitochondrial mutations. *Sci Adv* 2025;**11**:eadw4954.

Assou S, Al-edani T, Haouzi D, Philippe N, Lecellier C-H, Piquemal D, Commes T, Aït-Ahmed O, Dechaud H, Hamamah S. MicroRNAs: new candidates for the regulation of the human cumulus-oocyte complex. *Hum Reprod* 2013;**28**:3038–3049.

Barone S, Sarogni P, Valli R, Pallotta MM, Silvia G, Frattini A, Khan AW, Rapalini E, Parri C, Musio A. Chromosome Missegregation in Single Human Oocytes Is Related to the Age and Gene Expression Profile. *Int J Mol Sci* 2020;**21**:1934.

Barragán M, Pons J, Ferrer-Vaquer A, Cornet-Bartolomé D, Schweitzer A, Hubbard J, Auer H, Rodolosse A, Vassena R. The transcriptome of human oocytes is related to age and ovarian reserve. *MHR: Basic science of reproductive medicine* 2017;**23**:535–548.

Battaglia R, Vento ME, Borzì P, Ragusa M, Barbagallo D, Arena D, Purrello M, Di Pietro C. Non-coding RNAs in the Ovarian Follicle. *Front Genet* 2017;**8**:57.

Battaglia R, Vento ME, Ragusa M, Barbagallo D, La Ferlita A, Di Emidio G, Borzí P, Artini PG, Scollo P, Tatone C, *et al.* MicroRNAs Are Stored in Human MII Oocyte and Their Expression Profile Changes in Reproductive Aging. *Biol Reprod* 2016;**95**:131.

Bothun AM, Gao Y, Takai Y, Ishihara O, Seki H, Karger B, Tilly JL, Woods DC. Quantitative Proteomic Profiling of the Human Ovary from Early to Mid-Gestation Reveals Protein Expression Dynamics of Oogenesis and Folliculogenesis. *Stem Cells and Development* 2018;**27**:723–735.

Boucret L, Bris C, Seegers V, Goudenège D, Desquiret-Dumas V, Domin-Bernhard M, Ferré-L'Hotellier V, Bouet PE, Descamps P, Reynier P, *et al.* Deep sequencing shows that oocytes are not prone to accumulate mtDNA heteroplasmic mutations during ovarian ageing. *Hum Reprod* 2017;**32**:2101–2109.

Burnik Papler T, Vrtacnik Bokal E, Maver A, Kopitar AN, Lovrečić L. Transcriptomic Analysis and Meta-Analysis of Human Granulosa and Cumulus Cells. Feltus FA (ed). *PLoS ONE* 2015;**10**:e0136473.

Cadenas J, Adrados CS, Kristensen SG, Macklon KT, Greve VH, Fedder J, Mamsen LS, Andersen CY. Transcriptional Changes in Cumulus Cells During In Vitro Maturation of Human Oocytes. *Mol Reprod Dev* 2025;**92**:e70035.

Cadenas J, Poulsen LC, Nikiforov D, Grøndahl ML, Kumar A, Bahnu K, Englund ALM, Malm J, Marko-Varga G, Pla I, *et al.* Regulation of human oocyte maturation *in vivo* during the final maturation of follicles. *Human Reproduction* 2023;**38**:686–700.

Caniçais C, Sobral D, Vasconcelos S, Cunha M, Pinto A, Mesquita Guimarães J, Santos F, Barros A, Dória S, Marques CJ. Transcriptomic analysis and epigenetic regulators in human oocytes at different stages of oocyte meiotic maturation. *Dev Biol* 2025;**519**:55–64.

Caponnetto A, Battaglia R, Ferrara C, Vento ME, Borzì P, Paradiso M, Scollo P, Purrello M, Longobardi S, D'Hooghe T, *et al.* Down-regulation of long non-coding RNAs in reproductive aging and analysis of the lncRNA-miRNA-mRNA networks in human cumulus cells. *J Assist Reprod Genet* 2022;**39**:919–931.

Chen L, Ding B, Wu L, Qiu J, Li Q, Ye Z, Yang J. Transcriptome Analysis Reveals the Mechanism of Natural Ovarian Ageing. *Front Endocrinol* 2022;**13**:918212.

Chen P, Li W, Liu X, Wang Y, Mai H, Huang R. Circular RNA expression profiles of ovarian granulosa cells in advanced-age women explain new mechanisms of ovarian aging. *Epigenomics* 2022;**14**:1029–1038.

Chitiashvili T, Dror I, Kim R, Hsu F-M, Chaudhari R, Pandolfi E, Chen D, Liebscher S, Schenke-Layland K, Plath K, *et al.* Female human primordial germ cells display X-chromosome dosage compensation despite the absence of X-inactivation. *Nat Cell Biol* 2020;**22**:1436–1446.

Cornet-Bartolomé D, Barragán M, Zambelli F, Ferrer-Vaquer A, Tiscornia G, Balcells S, Rodriguez A, Grinberg D, Vassena R. Human oocyte meiotic maturation is associated with a specific profile of alternatively spliced transcript isoforms. *Mol Reprod Dev* 2021;**88**:605–617.

Del Valle I, Buonocore F, Duncan AJ, Lin L, Barenco M, Parnaik R, Shah S, Hubank M, Gerrelli D, Achermann JC. A genomic atlas of human adrenal and gonad development. *Wellcome Open Res* 2017;**2**:25.

Devrukhkar PR, Watson MA, Soygur B, Wu F, Bons J, Anvari H, Atazhanova T, Martin N, Tran T, Schneider K, *et al.* A Comprehensive Multiomics Signature of Doxorubicin-Induced Cellular Senescence in the Postmenopausal Human Ovary. *Aging Cell* 2025:e70111.

Dhori X, Gioiosa S, Gonfloni S. An integrated analysis of multiple datasets reveals novel gene signatures in human granulosa cells. *Sci Data* 2024;**11**:972.

Diedrichs F, Mlody B, Matz P, Fuchs H, Chavez L, Drews K, Adjaye J. Comparative molecular portraits of human unfertilized oocytes and primordial germ cells at 10 weeks of gestation. *Int J Dev Biol* 2012;**56**:789–797.

Diez-Fraile A, Lammens T, Tilleman K, Witkowski W, Verhasselt B, De Sutter P, Benoit Y, Espeel M, D'Herde K. Age-associated differential microRNA levels in human follicular fluid reveal pathways potentially determining fertility and success of in vitro fertilization. *Hum Fertil (Camb)* 2014;**17**:90–98.

Dong J-P, Dai Z-H, Jiang Z-X, He Y, Wang L, Liao Q-Y, Sun N-X, Wang Y-N, Sun S-H, Lin W, *et al.* CD24: a marker of granulosa cell subpopulation and a mediator of ovulation. *Cell Death Dis* 2019;**10**:791.

Ernst EH, Franks S, Hardy K, Villesen P, Lykke-Hartmann K. Granulosa cells from human primordial and primary follicles show differential global gene expression profiles. *Hum Reprod* 2018;**33**:666–679.

Ernst EH, Grøndahl ML, Grund S, Hardy K, Heuck A, Sunde L, Franks S, Andersen CY, Villesen P, Lykke-Hartmann K. Dormancy and activation of human oocytes from primordial and primary follicles: molecular clues to oocyte regulation. *Human Reproduction* 2017;**32**:1684–1700.

Fan X, Bialecka M, Moustakas I, Lam E, Torrens-Juaneda V, Borggreven NV, Trouw L, Louwe LA, Pilgram GSK, Mei H, *et al.* Single-cell reconstruction of follicular remodeling in the human adult ovary. *Nat Commun* 2019;**10**:3164.

Fan X, Moustakas I, Bialecka M, Del Valle JS, Overeem AW, Louwe LA, Pilgram GSK, Van Der Westerlaken LAJ, Mei H, Chuva De Sousa Lopes SM. Single-Cell Transcriptomics Analysis of Human Small Antral Follicles. *IJMS* 2021;**22**:11955.

Fowler PA, Flannigan S, Mathers A, Gillanders K, Lea RG, Wood MJ, Maheshwari A, Bhattacharya S, Collie-Duguid ESR, Baker PJ, *et al.* Gene expression analysis of human fetal ovarian primordial follicle formation. *J Clin Endocrinol Metab* 2009;**94**:1427–1435.

Galatidou S, Petelski AA, Pujol A, Lattes K, Latorraca LB, Fair T, Popovic M, Vassena R, Slavov N, Barragán M. Single-cell proteomics reveals decreased abundance of proteostasis and meiosis proteins in advanced maternal age oocytes. *Mol Hum Reprod* 2024;**30**:gaae023.

Garcia-Alonso L, Lorenzi V, Mazzeo CI, Alves-Lopes JP, Roberts K, Sancho-Serra C, Engelbert J, Marečková M, Gruhn WH, Botting RA, *et al.* Single-cell roadmap of human gonadal development. *Nature* 2022;**607**:540–547.

Ghevaria H, SenGupta S, Naja R, Odia R, Exeter H, Serhal P, Gonzalez XV, Sun X, Delhanty J. Next Generation Sequencing Detects Premeiotic Errors in Human Oocytes. *IJMS* 2022;**23**:665.

Gkountela S, Zhang KX, Shafiq TA, Liao W-W, Hargan-Calvopiña J, Chen P-Y, Clark AT. DNA Demethylation Dynamics in the Human Prenatal Germline. *Cell* 2015;**161**:1425–1436.

Gokyer D, Akinboro S, Zhou LT, Kleinhans A, Laronda MM, Duncan FE, Riley JK, Goldman KN, Babayev E. The oocyte microenvironment is altered in adolescents compared to oocyte donors. *Hum Reprod Open* 2024;**2024**:hoae047.

Grøndahl ML, Yding Andersen C, Bogstad J, Nielsen FC, Meinertz H, Borup R. Gene expression profiles of single human mature oocytes in relation to age. *Human Reproduction* 2010;**25**:957–968.

Gu Y, Zhang X, Wang R, Wei Y, Peng H, Wang K, Li H, Ji Y. Metabolomic profiling of exosomes reveals age-related changes in ovarian follicular fluid. *Eur J Med Res* 2024;**29**:4.

Guahmich NL, Man L, Wang J, Arazi L, Kallinos E, Topper-Kroog A, Grullon G, Zhang K, Stewart J, Schatz-Siemers N, *et al.* Human theca arises from ovarian stroma and is comprised of three discrete subtypes. *Commun Biol* 2023;**6**:7.

Guo F, Yan L, Guo H, Li L, Hu B, Zhao Y, Yong J, Hu Y, Wang X, Wei Y, *et al.* The Transcriptome and DNA Methylome Landscapes of Human Primordial Germ Cells. *Cell* 2015;**161**:1437–1452.

Guo Y, Cai L, Liu X, Ma L, Zhang H, Wang B, Qi Y, Liu J, Diao F, Sha J, *et al.* Single-Cell Quantitative Proteomic Analysis of Human Oocyte Maturation Revealed High Heterogeneity in In Vitro-Matured Oocytes. *Mol Cell Proteomics* 2022;**21**:100267.

Hou Y, Fan W, Yan L, Li R, Lian Y, Huang J, Li J, Xu L, Tang F, Xie XS, *et al.* Genome Analyses of Single Human Oocytes. *Cell* 2013;**155**:1492–1506.

Hu W, Zeng H, Shi Y, Zhou C, Huang J, Jia L, Xu S, Feng X, Zeng Y, Xiong T, *et al.* Single-cell transcriptome and translatome dual-omics reveals potential mechanisms of human oocyte maturation. *Nat Commun* 2022;**13**:5114.

Huang J, Chen P, Jia L, Li T, Yang X, Liang Q, Zeng Y, Liu J, Wu T, Hu W, *et al.* Multi-Omics Analysis Reveals Translational Landscapes and Regulations in Mouse and Human Oocyte Aging. *Adv Sci (Weinh)* 2023;**10**:e2301538.

Hurwitz JM, Jindal S, Greenseid K, Berger D, Brooks A, Santoro N, Pal L. Reproductive aging is associated with altered gene expression in human luteinized granulosa cells. *Reprod Sci* 2010;**17**:56–67.

Jain N, Li JL, Tong L, Jasmine F, Kibriya MG, Demanelis K, Oliva M, Chen LS, Pierce BL. DNA methylation correlates of chronological age in diverse human tissue types. *Epigenetics & Chromatin* 2024;**17**:25.

Jiang Z-X, Wang Y-N, Li Z-Y, Dai Z-H, He Y, Chu K, Gu J-Y, Ji Y-X, Sun N-X, Yang F, *et al.* The m6A mRNA demethylase FTO in granulosa cells retards FOS-dependent ovarian aging. *Cell Death Dis* 2021;**12**:744.

Jin C, Wang X, Yang J, Kim S, Hudgins AD, Gamliel A, Pei M, Contreras D, Devos M, Guo Q, *et al.* Molecular and genetic insights into human ovarian aging from single-nuclei multi-omics analyses. *Nat Aging* 2025;**5**:275–290.

Jones ASK, Hannum DF, Machlin JH, Tan A, Ma Q, Ulrich ND, Shen Y-C, Ciarelli M, Padmanabhan V, Marsh EE, *et al.* Cellular atlas of the human ovary using morphologically guided spatial transcriptomics and single-cell sequencing. *Sci Adv* 2024;**10**:eadm7506.

Kavarthapu R, Lou H, Pham T, Do H, Soliman ME, Badger T, Balasubramanian R, Huyhn V, De La Luz Sierra M, Yano Maher JC, *et al.* Single-nucleus and spatial transcriptomics of paediatric ovary: Molecular insights into the dysregulated signalling pathways underlying premature ovarian insufficiency in classic galactosemia. *Clin Transl Med* 2024;**14**:e70043.

Kristensen SG, Ebbesen P, Andersen CY. Transcriptional profiling of five isolated size-matched stages of human preantral follicles. *Molecular and Cellular Endocrinology* 2015;**401**:189–201.

Lecluze E, Rolland AD, Filis P, Evrard B, Leverrier-Penna S, Maamar MB, Coiffec I, Lavoué V, Fowler PA, Mazaud-Guittot S, *et al.* Dynamics of the transcriptional landscape during human fetal testis and ovary development. *Human Reproduction* 2020;**35**:1099–1119.

Lengyel E, Li Y, Weigert M, Zhu L, Eckart H, Javellana M, Ackroyd S, Xiao J, Olalekan S, Glass D, *et al.* A molecular atlas of the human postmenopausal fallopian tube and ovary from single-cell RNA and ATAC sequencing. *Cell Reports* 2022;**41**:111838.

Lewandowska AE, Macur K, Czaplewska P, Liss J, Łukaszuk K, Ołdziej S. Qualitative and Quantitative Analysis of Proteome and Peptidome of Human Follicular Fluid Using Multiple Samples from Single Donor with LC-MS and SWATH Methodology. *J Proteome Res* 2017;**16**:3053–3067.

Li J, Lu M, Zhang P, Hou E, Li T, Liu X, Xu X, Wang Z, Fan Y, Zhen X, *et al.* Aberrant spliceosome expression and altered alternative splicing events correlate with maturation deficiency in human oocytes. *Cell Cycle* 2020;**19**:2182–2194.

Li L, Dong J, Yan L, Yong J, Liu X, Hu Y, Fan X, Wu X, Guo H, Wang X, *et al.* Single-Cell RNA-Seq Analysis Maps Development of Human Germline Cells and Gonadal Niche Interactions. *Cell Stem Cell* 2017;**20**:858-873.e4.

Liu Q, Wei F, Wang J, Liu H, Zhang H, Liu M, Liu K, Ye Z. Molecular mechanisms regulating natural menopause in the female ovary: a study based on transcriptomic data. *Front Endocrinol* 2023;**14**:1004245.

Liu Y, Tao W, Wu S, Zhang Y, Nie H, Hou Z, Zhang J, Yang Z, Chen Z-J, Wang J, *et al.* Maternal mRNA deadenylation is defective in in vitro matured mouse and human oocytes. *Nat Commun* 2024;**15**:5550.

Liu Z, Zhou Q, Zan J, Tian J, Zhang Y, Wu F, Zhao H, Peng Q, Liu S, Chen Q, *et al.* Proteomic Analysis of Human Follicular Fluid-Derived Exosomes Reveals That Insufficient Folliculogenesis in Aging Women is Associated With Infertility. *Mol Cell Proteomics* 2025;**24**:100930.

Llonch S, Barragán M, Nieto P, Mallol A, Elosua-Bayes M, Lorden P, Ruiz S, Zambelli F, Heyn H, Vassena R, *et al.* Single human oocyte transcriptome analysis reveals distinct maturation stage-dependent pathways impacted by age. *Aging Cell* 2021;**20**:e13360.

Mamsen LS, Ernst EH, Borup R, Larsen A, Olesen RH, Ernst E, Anderson RA, Kristensen SG, Andersen CY. Temporal expression pattern of genes during the period of sex differentiation in human embryonic gonads. *Sci Rep* 2017;**7**:15961.

Mao R, Cai Z, Wang T, Li Y, Tian S, Li D, Li P. Comparative study of the three-dimensional genomes of granulosa cells in germinal vesicle and metaphase II follicles. *Front Genet* 2024;**15**:1480153.

Markholt S, Grøndahl ML, Ernst EH, Andersen CY, Ernst E, Lykke-Hartmann K. Global gene analysis of oocytes from early stages in human folliculogenesis shows high expression of novel genes in reproduction. *MHR: Basic science of reproductive medicine* 2012;**18**:96–110.

McGlacken-Byrne SM, Del Valle I, Xenakis T, Simcock IC, Suntharalingham JP, Buonocore F, Crespo B, Moreno N, Liptrot D, Niola P, *et al.* Mapping the anatomical and transcriptional landscape of early human fetal ovary development. *Sci Rep* 2025;**15**:15814.

Méar L, Sutantiwanichkul T, Östman J, Damdimopoulou P, Lindskog C. Spatial Proteomics for Further Exploration of Missing Proteins: A Case Study of the Ovary. *J Proteome Res* 2023;**22**:1071–1079.

Molinari E, Bar H, Pyle AM, Patrizio P. Transcriptome analysis of human cumulus cells reveals hypoxia as the main determinant of follicular senescence. *Mol Hum Reprod* 2016;**22**:866–876.

Ntostis P, Iles D, Kokkali G, Vaxevanoglou T, Kanavakis E, Pantou A, Huntriss J, Pantos K, Picton HM. The impact of maternal age on gene expression during the GV to MII transition in euploid human oocytes. *Hum Reprod* 2021;**37**:80–92.

Olsen KW, Castillo-Fernandez J, Zedeler A, Freiesleben NC, Bungum M, Chan AC, Cardona A, Perry JRB, Skouby SO, Borup R, *et al.* A distinctive epigenetic ageing profile in human granulosa cells. *Human Reproduction* 2020;**35**:1332–1345.

Orvieto R, Nayshool O, Cohen L, Yung Y, Aizer A, Saar EG, Dominissini D. Impact of aging on gene expression in human oocytes: a comparative analysis of young and older patients. *Reprod Biol Endocrinol* 2025;**23**:111.

Ouandaogo ZG, Frydman N, Hesters L, Assou S, Haouzi D, Dechaud H, Frydman R, Hamamah S. Differences in transcriptomic profiles of human cumulus cells isolated from oocytes at GV, MI and MII stages after in vivo and in vitro oocyte maturation. *Human Reproduction* 2012;**27**:2438–2447.

Ouni E, Nedbal V, Da Pian M, Cao H, Haas KT, Peaucelle A, Van Kerk O, Herinckx G, Marbaix E, Dolmans M-M, *et al.* Proteome-wide and matrisome-specific atlas of the human ovary computes fertility biomarker candidates and open the way for precision oncofertility. *Matrix Biol* 2022;**109**:91–120.

Ouni E, Vertommen D, Chiti MC, Dolmans M-M, Amorim CA. A Draft Map of the Human Ovarian Proteome for Tissue Engineering and Clinical Applications. *Molecular & Cellular Proteomics* 2019;**18**:S159–S173.

Pei W, Cao X, Zhang P, Fu L, Zhou P, Liu Y, Fan Y, Xu F, Cao C, Yu Y. Spatiotemporal Transcriptome Analysis Reveals Activation of the AP1 Pathway in the Ovarian Microenvironment during the Transition from Premenopause to Postmenopause. *Aging Dis* 2024;**15**:445–448.

Pietroforte S, Barragan Monasterio M, Ferrer-Vaquer A, Irimia M, Ibáñez E, Popovic M, Vassena R, Zambelli F. Specific processing of meiosis-related transcript is linked to final maturation in human oocytes. *Molecular Human Reproduction* 2023;**29**:gaad021.

Pla I, Sanchez A, Pors SE, Pawlowski K, Appelqvist R, Sahlin KB, Poulsen LLC, Marko-Varga G, Andersen CY, Malm J. Proteome of fluid from human ovarian small antral follicles reveals insights in folliculogenesis and oocyte maturation. *Human Reproduction* 2021;**36**:756–770.

Poulsen LC, Bøtkjær JA, Østrup O, Petersen KB, Andersen CY, Grøndahl ML, Englund ALM. Two waves of transcriptomic changes in periovulatory human granulosa cells. *Human Reproduction* 2020;**35**:1230–1245.

Poulsen LLC, Englund ALM, Wissing MLM, Yding Andersen C, Borup R, Grøndahl ML. Human granulosa cells function as innate immune cells executing an inflammatory reaction during ovulation: a microarray analysis. *Molecular and Cellular Endocrinology* 2019;**486**:34–46.

Reyes JM, Silva E, Chitwood JL, Schoolcraft WB, Krisher RL, Ross PJ. Differing molecular response of young and advanced maternal age human oocytes to IVM. *Hum Reprod* 2017;**32**:2199–2208.

Rodríguez-Nuevo A, Torres-Sanchez A, Duran JM, De Guirior C, Martínez-Zamora MA, Böke E. Oocytes maintain ROS-free mitochondrial metabolism by suppressing complex I. *Nature* 2022;**607**:756–761.

Rogers HM, Gad A, Cork GK, Menjivar NG, Schoolcraft WB, Tesfaye D, Yuan Y. Age-related integrative transcriptomic profiling of human granulosa cells reveals mRNA-microRNA regulatory network associated with key ovulation dynamics†. *Biol Reprod* 2025;**112**:916–931.

Rooda I, Hassan J, Hao J, Wagner M, Moussaud-Lamodière E, Jääger K, Otala M, Knuus K, Lindskog C, Papaikonomou K, *et al.* In-depth analysis of transcriptomes in ovarian cortical follicles from children and adults reveals interfollicular heterogeneity. *Nat Commun* 2024;**15**:6989.

Ruth KS, Day FR, Hussain J, Martínez-Marchal A, Aiken CE, Azad A, Thompson DJ, Knoblochova L, Abe H, Tarry-Adkins JL, *et al.* Genetic insights into biological mechanisms governing human ovarian ageing. *Nature* 2021;**596**:393–397.

Shi H, Pan M, Sheng Y, Jia E, Wang Y, Dong J, Tu J, Bai Y, Cai L, Ge Q. Extracellular cell-free RNA profile in human large follicles and small follicles. *Front Cell Dev Biol* 2022;**10**:940336.

Smits MAJ, Janssens GE, Goddijn M, Hamer G, Houtkooper RH, Mastenbroek S. Longevity pathways are associated with human ovarian ageing. *Hum Reprod Open* 2021;**2021**:hoab020.

Smits MAJ, Schomakers BV, van Weeghel M, Wever EJM, Wüst RCI, Dijk F, Janssens GE, Goddijn M, Mastenbroek S, Houtkooper RH, *et al.* Human ovarian aging is characterized by oxidative damage and mitochondrial dysfunction. *Hum Reprod* 2023;**38**:2208–2220.

Taelman J, Czukiewska SM, Moustakas I, Chang YW, Hillenius S, van der Helm T, van der Meeren LE, Mei H, Fan X, Chuva de Sousa Lopes SM. Characterization of the human fetal gonad and reproductive tract by single-cell transcriptomics. *Dev Cell* 2024;**59**:529-544.e5.

Takeuchi H, Yamamoto M, Fukui M, Inoue A, Maezawa T, Nishioka M, Kondo E, Ikeda T, Matsumoto K, Miyamoto K. Single-cell profiling of transcriptomic changes during *in vitro* maturation of human oocytes. *Reprod Medicine & Biology* 2022;**21**:e12464.

Tong X-H, Xu B, Zhang Y-W, Liu Y-S, Ma C-H. Research resources: comparative microRNA profiles in human corona radiata cells and cumulus oophorus cells detected by next-generation small RNA sequencing. *PLoS One* 2014;**9**:e106706.

Varik I, Saretok KJ, Rosenberg K, Quintero I, Puhka M, Volkova N, Trošin A, Guazzi P, Velthut-Meikas A. Small and Large Extracellular Vesicles From Human Preovulatory Follicular Fluid Display Distinct ncRNA Cargo Profiles and Differential Effects on KGN Granulosa Cells. *J Extracell Vesicles* 2025;**14**:e70119.

Velthut-Meikas A, Simm J, Tuuri T, Tapanainen JS, Metsis M, Salumets A. Research resource: small RNA-seq of human granulosa cells reveals miRNAs in FSHR and aromatase genes. *Mol Endocrinol* 2013;**27**:1128–1141.

Virant-Klun I, Leicht S, Hughes C, Krijgsveld J. Identification of Maturation-Specific Proteins by Single-Cell Proteomics of Human Oocytes. *Molecular & Cellular Proteomics* 2016;**15**:2616–2627.

Wagner M, Yoshihara M, Douagi I, Damdimopoulos A, Panula S, Petropoulos S, Lu H, Pettersson K, Palm K, Katayama S, *et al.* Single-cell analysis of human ovarian cortex identifies distinct cell populations but no oogonial stem cells. *Nat Commun* 2020;**11**:1147.

Wang C, Zhao X, Wu Z, Huang G, Lin R, Chen H, Xu K, Sun K, Zhou H, Shu J. Identification of Differentially Expressed mRNAs and miRNAs and Related Regulatory Networks in Cumulus Oophorus Complexes Associated with Fertilization. *Reprod Sci* 2024;**31**:1408–1419.

Wang R, Liu X, Li L, Yang M, Yong J, Zhai F, Wen L, Yan L, Qiao J, Tang F. Dissecting Human Gonadal Cell Lineage Specification and Sex Determination Using A Single-cell RNA-seq Approach. *Genomics, Proteomics & Bioinformatics* 2022;**20**:223–245.

Williams Z, Morozov P, Mihailovic A, Lin C, Puvvula PK, Juranek S, Rosenwaks Z, Tuschl T. Discovery and Characterization of piRNAs in the Human Fetal Ovary. *Cell Rep* 2015;**13**:854–863.

Wu H, Zhu R, Zheng B, Liao G, Wang F, Ding J, Li H, Li M. Single-Cell Sequencing Reveals an Intrinsic Heterogeneity of the Preovulatory Follicular Microenvironment. *Biomolecules* 2022;**12**:231.

Wu M, Tang W, Chen Y, Xue L, Dai J, Li Y, Zhu X, Wu C, Xiong J, Zhang J, *et al.* Spatiotemporal transcriptomic changes of human ovarian aging and the regulatory role of FOXP1. *Nat Aging* 2024;**4**:527–545.

Wu Y-W, Li S, Zheng W, Li Y-C, Chen L, Zhou Y, Deng Z-Q, Lin G, Fan H-Y, Sha Q-Q. Dynamic mRNA degradome analyses indicate a role of histone H3K4 trimethylation in association with meiosis-coupled mRNA decay in oocyte aging. *Nat Commun* 2022;**13**:3191.

Wyse BA, Fuchs Weizman N, Kadish S, Balakier H, Sangaralingam M, Librach CL. Transcriptomics of cumulus cells – a window into oocyte maturation in humans. *J Ovarian Res* 2020;**13**:93.

Xu B, Zhang Y-W, Zheng S-X, Tong X-H, Liu Y-S. Expression Profile of microRNAs and Their Targeted Pathways in Human Ovaries Detected by Next-Generation Small RNA Sequencing. *DNA Cell Biol* 2016;**35**:226–234.

Xu Y-W, Wang B, Ding C-H, Li T, Gu F, Zhou C. Differentially expressed micoRNAs in human oocytes. *J Assist Reprod Genet* 2011;**28**:559–566.

Yan R, Gu C, You D, Huang Z, Qian J, Yang Q, Cheng X, Zhang L, Wang H, Wang P, *et al.* Decoding dynamic epigenetic landscapes in human oocytes using single-cell multi-omics sequencing. *Cell Stem Cell* 2021;**28**:1641-1656.e7.

Yang Q, Li R, Lyu Q, Hou L, Liu Z, Sun Q, Liu M, Shi H, Xu B, Yin M, *et al.* Single-cell CAS-seq reveals a class of short PIWI-interacting RNAs in human oocytes. *Nat Commun* 2019;**10**:3389.

Yao H, Zhang D, Jin H, Guo Y, Liu Y, Wang S, Li T, Yuan S, Lu G, Sun Y. RNA multi-omics in single cells reveal rhythmical RNA reshaping during human and mouse oocyte maturation. *BMC Biol* 2025;**23**:147.

Ye M, Yang Z-Y, Zhang Y, Xing Y-X, Xie Q-G, Zhou J-H, Wang L, Xie W, Kee K, Chian R-C. Single-cell multiomic analysis of in vivo and in vitro matured human oocytes. *Human Reproduction* 2020;**35**:886–900.

Yerushalmi GM, Salmon-Divon M, Yung Y, Maman E, Kedem A, Ophir L, Elemento O, Coticchio G, Dal Canto M, Mignini Renzinu M, *et al.* Characterization of the human cumulus cell transcriptome during final follicular maturation and ovulation. *Mol Hum Reprod* 2014;**20**:719–735.

Yi J, Hu H, Shi P, Shi S, Zhao J, Xu L, Yang W, Li B, Zhu J, Zou S. Differential analysis of quantitative proteome and acetyl-proteome profiling between premenopausal and postmenopausal ovarian tissues. *Clin Proteomics* 2018;**15**:36.

Yoo SW, Savchev S, Sergott L, Rezai T, Lopez MF, Von Wald T, Eaton JL, Reindollar R, Usheva A. A Large Network of Interconnected Signaling Pathways in Human Ovarian Follicles is Supported by the Gene Expression Activity of the Granulosa Cells. *Reprod Sci* 2011;**18**:476–484.

Yu B, Dong X, Gravina S, Kartal Ö, Schimmel T, Cohen J, Tortoriello D, Zody R, Hawkins RD, Vijg J. Genome-wide, Single-Cell DNA Methylomics Reveals Increased Non-CpG Methylation during Human Oocyte Maturation. *Stem Cell Reports* 2017;**9**:397–407.

Yu B, Doni Jayavelu N, Battle SL, Mar JC, Schimmel T, Cohen J, Hawkins RD. Single-cell analysis of transcriptome and DNA methylome in human oocyte maturation. El-Maarri O (ed). *PLoS ONE* 2020;**15**:e0241698.

Yu B, Russanova VR, Gravina S, Hartley S, Mullikin JC, Ignezweski A, Graham J, Segars JH, DeCherney AH, Howard BH. DNA methylome and transcriptome sequencing in human ovarian granulosa cells links age-related changes in gene expression to gene body methylation and 3′-end GC density. *Oncotarget* 2015;**6**:3627–3643.

Yu Y, Zhu M-J, Wei C-F, Yang J, Song J-Y, Dong L, Xiang S, Zhang L, Qiu Y, Lian F. Age-related differential gene expression in granulosa cells and its effects on fertility using high-throughput transcriptomics. *Syst Biol Reprod Med* 2022;**68**:190–202.

Yuan L, Yin P, Yan H, Zhong X, Ren C, Li K, Chin Heng B, Zhang W, Tong G. Single-cell transcriptome analysis of human oocyte ageing. *J Cellular Molecular Medi* 2021;**25**:6289–6303.

Zhang M, Lu Y, Chen Y, Zhang Y, Xiong B. Insufficiency of melatonin in follicular fluid is a reversible cause for advanced maternal age-related aneuploidy in oocytes. *Redox Biol* 2020;**28**:101327.

Zhang X, Yang J, Yang W, Cui N, Duan T, Li S, Cao J, Bush SJ, Tong G. Discordant effects of maternal age on the human MII oocyte transcriptome. *Mol Hum Reprod* 2025:gaaf038.

Zhang Y-R, Yin Y, Guo S-M, Wang Y-F, Zhao G-N, Ji D-M, Zhou L-Q. The landscape of transcriptional profiles in human oocytes with different chromatin configurations. *J Ovarian Res* 2024;**17**:99.

Zhang Z, Luo L, Fan S, Bai S, Xu B. Single-cell proteomics analysis of human oocytes during GV-to-MI transition. *Hum Reprod* 2025;**40**:1332–1343.

Zhao H, Li T, Zhao Y, Tan T, Liu C, Liu Y, Chang L, Huang N, Li C, Fan Y, *et al.* Single-Cell Transcriptomics of Human Oocytes: Environment-Driven Metabolic Competition and Compensatory Mechanisms During Oocyte Maturation. *Antioxidants & Redox Signaling* 2019;**30**:542–559.

Zhao X, Zhao F, Yan L, Wu J, Fang Y, Wang C, Xin Z, Yang X. Long non-coding ribonucleic acid SNHG18 induced human granulosa cell apoptosis via disruption of glycolysis in ovarian aging. *J Ovarian Res* 2024;**17**:185.
